# Characterization of a putative orexin receptor in *Ciona intestinalis* sheds light on the evolution of the orexin/hypocretin system in chordates

**DOI:** 10.1038/s41598-024-56508-1

**Published:** 2024-04-02

**Authors:** Maiju K. Rinne, Lauri Urvas, Ilona Mandrika, Dāvids Fridmanis, Darren M. Riddy, Christopher J. Langmead, Jyrki P. Kukkonen, Henri Xhaard

**Affiliations:** 1 Drug Research Program, Division of Pharmaceutical Chemistry and Technology, Faculty of Pharmacy, University of Helsinki, POB 56, 00014 Helsinki, Finland; 2 Biochemistry and Cell Biology, Department of Veterinary Biosciences, Faculty of Veterinary Medicine, University of Helsinki, POB 66, 00014 Helsinki, Finland; 3 Department of Pharmacology, Medicum, University of Helsinki, POB 63, 00014 Helsinki, Finland; 4 Laboratoire d’Innovation Thérapeutique, Faculté de Pharmacie, Université de Strasbourg, Illkirch-Graffenstaden, France; 5 Latvian Biomedical Research and Study Centre, Riga, Latvia; 6 Drug Discovery Biology, Monash Institute of Pharmaceutical Sciences, Monash University, Parkville, Australia

**Keywords:** Orexin, Hypocretin, Calcium, Receptor binding, *Ciona intestinalis*, Molecular biology, Structural biology, Cell biology, Cell signalling, Computational biology and bioinformatics, Classification and taxonomy, Computational models, Data mining, Phylogeny, Protein function predictions, Protein structure predictions, Evolution, Molecular evolution

## Abstract

Tunicates are evolutionary model organisms bridging the gap between vertebrates and invertebrates. A genomic sequence in *Ciona intestinalis* (CiOX) shows high similarity to vertebrate orexin receptors and protostome allatotropin receptors (ATR). Here, molecular phylogeny suggested that CiOX is divergent from ATRs and human orexin receptors (hOX_1/2_). However, CiOX appears closer to hOX_1/2_ than to ATR both in terms of sequence percent identity and in its modelled binding cavity, as suggested by molecular modelling. CiOX was heterologously expressed in a recombinant HEK293 cell system. Human orexins weakly but concentration-dependently activated its G_q_ signalling (Ca^2+^ elevation), and the responses were inhibited by the non-selective orexin receptor antagonists TCS 1102 and almorexant, but only weakly by the OX_1_-selective antagonist SB-334867. Furthermore, the 5-/6-carboxytetramethylrhodamine (TAMRA)-labelled human orexin-A was able to bind to CiOX. Database mining was used to predict a potential endogenous *C. intestinalis* orexin peptide (Ci-orexin-A). Ci-orexin-A was able to displace TAMRA-orexin-A, but not to induce any calcium response at the CiOX. Consequently, we suggested that the orexin signalling system is conserved in *Ciona intestinalis*, although the relevant peptide-receptor interaction was not fully elucidated.

## Introduction

The orexin (also called hypocretin) system in mammals is composed of two receptors (OX_1_ and OX_2_) and two peptides (orexin-A and orexin-B). The orexin receptors belong to the large family of G protein-coupled receptors (GPCRs) and research data suggests that they signal through members of all heterotrimeric G protein families^1–3^. Both orexin peptides are formed through proteolytic cleavage of prepro-orexin (PPO) propeptide. Orexin receptors and peptides are highly conserved within mammals, with an amino acid sequence identity above 90% among rodents and primates for OX_1_, OX_2_ and orexin-A, and above 75% for orexin-B.

The orexin system is well-known for regulating the sleep–wake cycle in mammals^4^. Animal models deficient in orexinergic neurons, orexin receptors or orexin peptides show a narcolepsy-like phenotype, while human narcolepsy patients lack functional orexinergic neurons^5^. The mammalian orexin system has been also associated with many other important functions such as appetite, reward seeking, stress, and metabolic regulation, and thus it is an important drug target^5,6^. In contrast, its physiological roles outside mammalians, especially in invertebrates, are poorly understood.

Model species within invertebrate deuterostomes from the (sub)phyla of tunicates (e.g., the marine vase tunicate *Ciona intestinalis*), cephalochordates (e.g., lancelets) and echinoderms (e.g., sea urchin) can be used to bridge the gap between vertebrates and invertebrates^7–9^. Various neuropeptides and neuropeptide receptors have been identified in invertebrates with primitive nervous system, e.g., in tunicates^10,11^, cephalochordates^13,14^, echinoderms^8,12^ and non-bilaterian cnidarians^13^. Among these, orexin-like peptides have been suggested in echinoderms (*Asterias rubens*)^12^, cephalochordates (*Branchiostoma floridae*, *B. belcheri*, *B. japonicum*; amphioxus/lancelet)^14,15^ and hemichordates (*Saccoglossus kowalevskii*; acorn worm)^16^*.* Very few studies have assessed the functionality of these systems; in the amphioxus *B. japonicum* an orexin peptide/receptor system was shown to stimulate lipogenesis, induce expression of leptin and galanin, and be regulated by feeding and temperature^15^. In tunicates, no orexin peptides have been described to date. Nonetheless, the amino acid sequence for a putative orexin receptor (CiOX) has been suggested in *C. intestinalis* by automated annotation^17,18^.

In terms of amino acid sequences, the vertebrate OX_1_ and OX_2_ receptors are more closely related to protostome allatotropin receptors than to any vertebrate GPCRs, suggesting a common origin^16,19–21^. A common evolutionary origin of vertebrate orexin and allatotropin systems is further suggested by the presence of a “cryptic peptide” present in both prepro-allatotropins and hemichordate *S. kowalevskii* putative PPO^14,16^.

In this study, we aimed to compare the vertebrate (hOX_2_), insect ATR (*Manduca sexta* ATR) and the CiOX amino-acid sequences and three-dimensional structures, as well as to identify of the *C. intestinalis* putative orexin peptide. Furthermore, we characterized the function of the CiOX in a mammalian recombinant expression system.

## Results

### Primary and tertiary structure of the putative orexin receptor from *C. intestinalis*

CiOX has been automatically annotated as an orexin receptor in databases, and it is in fact slightly closer to hOX_1/2_ than protostome ATRs in terms of amino acid sequences: in the predicted transmembrane segments (TMs), the sequence identity between CiOX and hOX_1_ or hOX_2_ is 40–42% and between CiOX and ATRs 33–36% (Supplementary Fig. S1). Similar trends are seen when comparing the complete amino acid sequences between CiOX and hOX_1_ or hOX_2_ (32–33%) and between CiOX and ATRs (26–32%) (Supplementary Fig. S2). Beyond the TMs, especially the third intracellular loops of hOX_1/2_ and CiOX differ markedly in length: the loop of hOX_1_ contains 34 amino acids while the loop of CiOX contains 123 amino acids. Interestingly, the putative *C. savignyi* orexin receptor has 38 amino acids in its third intracellular loop.

**Figure 1:**
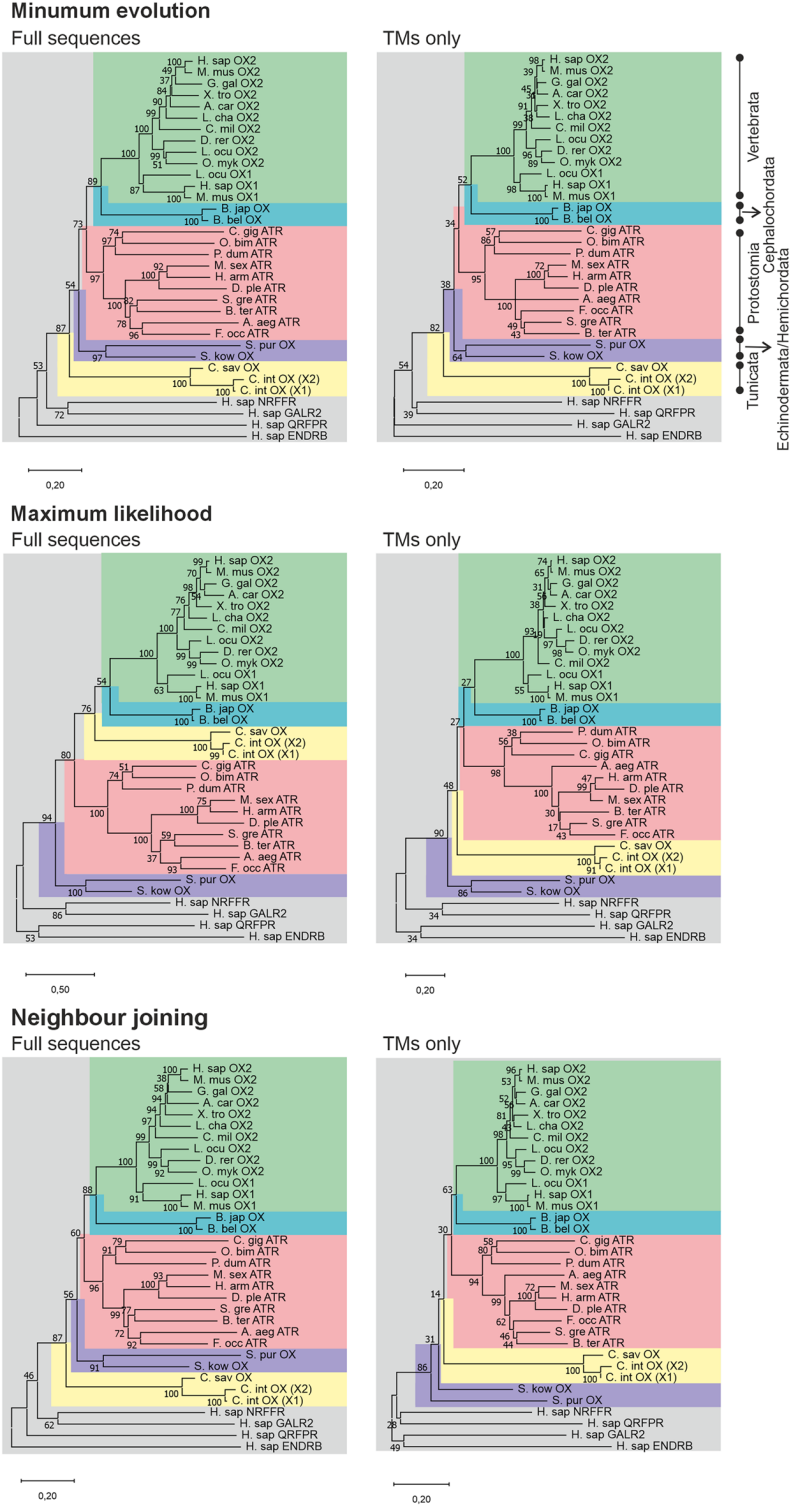
Phylogenetic analysis of putative *Ciona* orexin receptors based on amino acid sequences; for the species codes, see Supplementary Table S1, for the sequences used, see Supplementary Information 1, and for the alignments, see Supplementary Information 2–3. The CiOX gene encodes for two transcripts (X1 and X2), of which the X2 transcript isolated from *C. intestinalis* is used in this study. Trees were constructed based on either full sequences (left) or TMs only (right), with three different phylogenetic construction methods as indicated. Robustness was assessed with 500 × bootstrap method (values in % at the intersections of the branches). Green, vertebrate orexin receptors; turquoise, cephalochordate orexin receptors; pink, protostome ATRs; purple, echinoderms/hemichordates orexin receptors; and yellow tunicate orexin receptors. The tree is rooted (grey) with human sequences of NPFF1 (NPFFR), GAL_2_ (GALR2), QFR (QRFPR), and ET_B_ (ENDRB) receptors.

The hOX_1/2_ are more similar to ATRs than to CiOX in terms of sequence identity (48–54% vs. 40–42%; TMs only) as well as in the phylogenetic analysis (Fig. 1). The sequence alignment used for the phylogenetic analysis appeared of good quality, with gaps located in the regions structurally corresponding to loops and not within the TM regions. We constructed trees both with full sequences and with the TM regions only. The trees constructed with only the TM regions should be the least impacted by the sequence alignment methods; furthermore, TM regions represent the most evolutionary conserved regions of the receptors and thus are most relevant for phylogenetic studies. Altogether, the six trees present a consistent branching for four clades: vertebrate orexin receptors (green), cephalochordate orexin receptors (turquoise), and echinoderm/hemichordate orexin receptors (purple). The branching of the CiOX (yellow) and ATRs (pink) clades is more variable. Nonetheless, in five out of six phylogenetic trees, CiOX is branched away from protostome allatotropin and other chordate orexin receptors.

**Figure 2:**
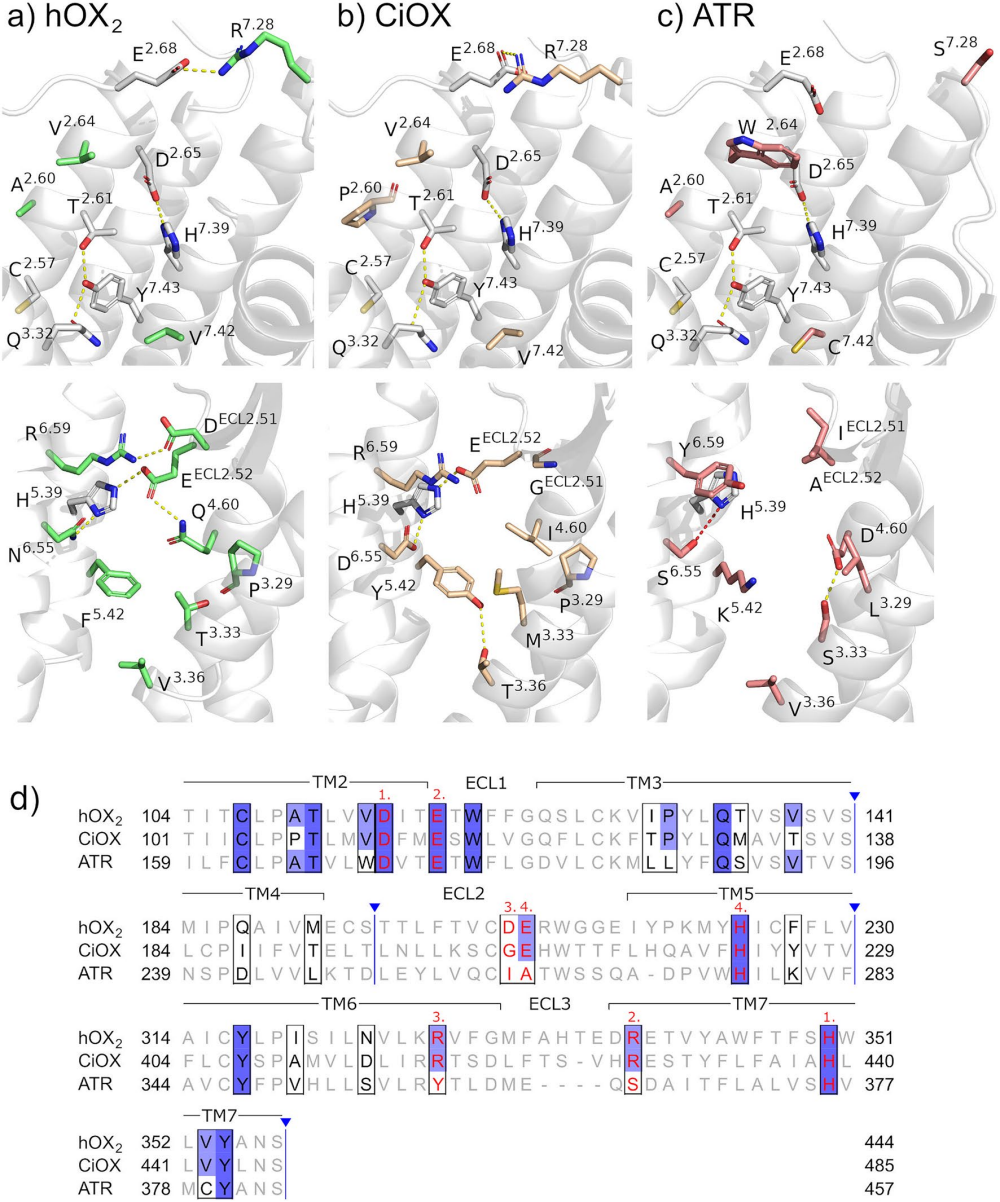
Comparison of the receptor binding sites. (**a**) The determined binding sites of hOX_2_ (PDB code 5WQC (**b**) homology model of CiOX and (**c**) homology model of *M. sexta* ATR are shown from two points of view. The top row presents mainly the TM2 and TM7 and the bottom row mainly the TM3, ECL2, TM5 and TM6. Conserved residues between hOX_2_, CiOX and ATR are uncoloured sticks, otherwise in green (hOX_2_), light brown (CiOX) and coral (ATR). Yellow dashes indicate hydrogen bonds and salt bridges, while red dashes indicate long distance (4.0 Å) between the heavy atoms. Numbering according to the Ballesteros–Weinstein convention^29^. (**d**) aligned protein sequences of hOX_2_, CiOX and ATR. Binding site residues in black boxes, conservation in gradient blue, residues involved in salt bridges in red letters, salt bridge connections in red numbers. Regions of the sequences not shown are indicated by blue vertical lines.

To clarify the issue, we compared the putative ligand binding cavities for hOX_2_, CiOX, and *M. sexta* ATR (Fig. 2). This was achieved through construction of homology models of CiOX and ATR and their comparison to the available X-ray structure of hOX_2_ (Protein Data Bank (PDB) code 5WQC^22^). This early study was conducted using the inactive form of the receptor, when no active structure was available, however amino acids lining the binding cavity are the same in both active and inactive conformations (see Modelling of the peptide–CiOX complexes). Furthermore, the models are robust to the different construction methods: The homology models presented in this manuscript are highly similar in their transmembrane regions to those produced using AlphaFold^23^ (Supplementary Fig. S3). As discussed below, only a minor part of the peptide—extremely conserved across vertebrate orexins—binds deep into this cavity.

The modelled binding site of CiOX resembles more the binding site of hOX_2_ than that of ATR. Out of the 26 residues that are located deep within the receptor binding pocket there are 16 conserved positions between hOX_2_ and CiOX, but only 10 conserved positions between hOX_2_ and ATR. The analysis highlights two hydrogen bond networks and four salt bridges lining the binding cavity (Fig. 2). Of those, both networks and three salt bridges are conserved or conservatively substituted when comparing the hOX_2_ and the CiOX binding sites. In contrast, comparison of hOX_2_ and ATR revealed only one conserved hydrogen bond network and one conserved salt bridge. Two of the salt bridges involve TM2 and TM7: D^2.65^–H^7.39^ is centrally located and present in all three receptors, while E^2.68^–R^7.28^ is present in the extracellular domain of hOX_2_ and CiOX (Fig. 2a,b) but not ATR (Fig. 2c). hOX_2_ also features two additional salt bridges (D^ECL2.51^–R^6.59^ and E^ECL2.52^–H^5.39^) connecting extracellular loop 2 (ECL2) and TMs 5 and 6 (Fig. 2a). The E^ECL2.52^–H^5.39^ salt bridge is conserved between hOX_2_ and CiOX, but not between hOX_2_ and ATR. Furthermore, the binding cavity features side chains (N/D^6.55^ and F/Y^5.42^) conservatively substituted between hOX_2_ and CiOX, which could extend the network towards the intracellular surface of the binding cavity. In hOX_2_, N^6.55^ has been described to form a hydrogen-bond with orexin-B^24^ and suvorexant^25^. The D^ECL2.51^–R^6.59^ salt bridge found in hOX_2_ is not conserved, but it is typical for distantly related GPCRs to be more divergent in their extracellular domains^26^.

Another important network of interacting side chains T^2.61^–Y^7.43^–Q^3.32^ is present in all three receptors. This hydrogen bond network stabilizes the inactive state of hOX_2_. In the active state the network is broken as Q^3.32^ adopts an upward-facing conformation in order to form a hydrogen bond with the agonist orexin-B^24^. The position 3.32 is well known to be involved in receptor activation, in particular in amine GPCRs^27^.

In hOX_2_, position F^5.42^ is packed together with T^3.33^ and V^3.36^ (Fig. 2a). F^5.42^ has been described as essential for orexin-A and small molecule binding^24,25,28^. Position 5.42 is also well-known to be involved in the activation of amine GPCRs^27^. A similar network can be formed in CiOX (Y^5.42^, M^3.33^ and T^3.36^; Y^5.42^–T^3.36^ hydrogen bond is seen in our models) (Fig. 2b) but not in ATR (K^5.42^, S^3.33^, V^3.36^; no stabilizing interactions) (Fig. 2c), indicating that a similar subpocket exists in hOX_2_ and CiOX. Additional potentially disruptive changes, that might affect orexin peptide binding, are also found: In the TM2 of CiOX, P^2.60^ replaces A^2.60^ of ATR and hOX_2_; and in the TM3 of ATR, L^3.29^ replaces P^3.29^ of CiOX and hOX_2_. These changes have the potential to be significant as they may shift the exposed regions of α-helices. Across hOX_2_, CiOX and ATR, the cavities are overall of similar sizes, with few notable differences: T/M^3.33^ (hOX_2_/CiOX) are bulkier residues than S^3.33^ (ATR), while W^2.64^ in ATR is bulkier than the valines found in CiOX and hOX_2_.

We further compared the binding site conservation by constructing a vertebrate orexin receptor consensus sequence and an allatotropin consensus sequence for the binding site residues (Supplementary Fig. S4). The binding site residues of CiOX share 62% sequence identity with the consensus of vertebrate orexin receptor binding site, while identity with the consensus of ATR binding site is only 42%. Thus, at the binding site, CiOX is more similar to the vertebrate orexin receptors than with respect to ATR receptors.

### Discovery of the candidate orexin peptide from *C. intestinalis*

**Figure 3:**
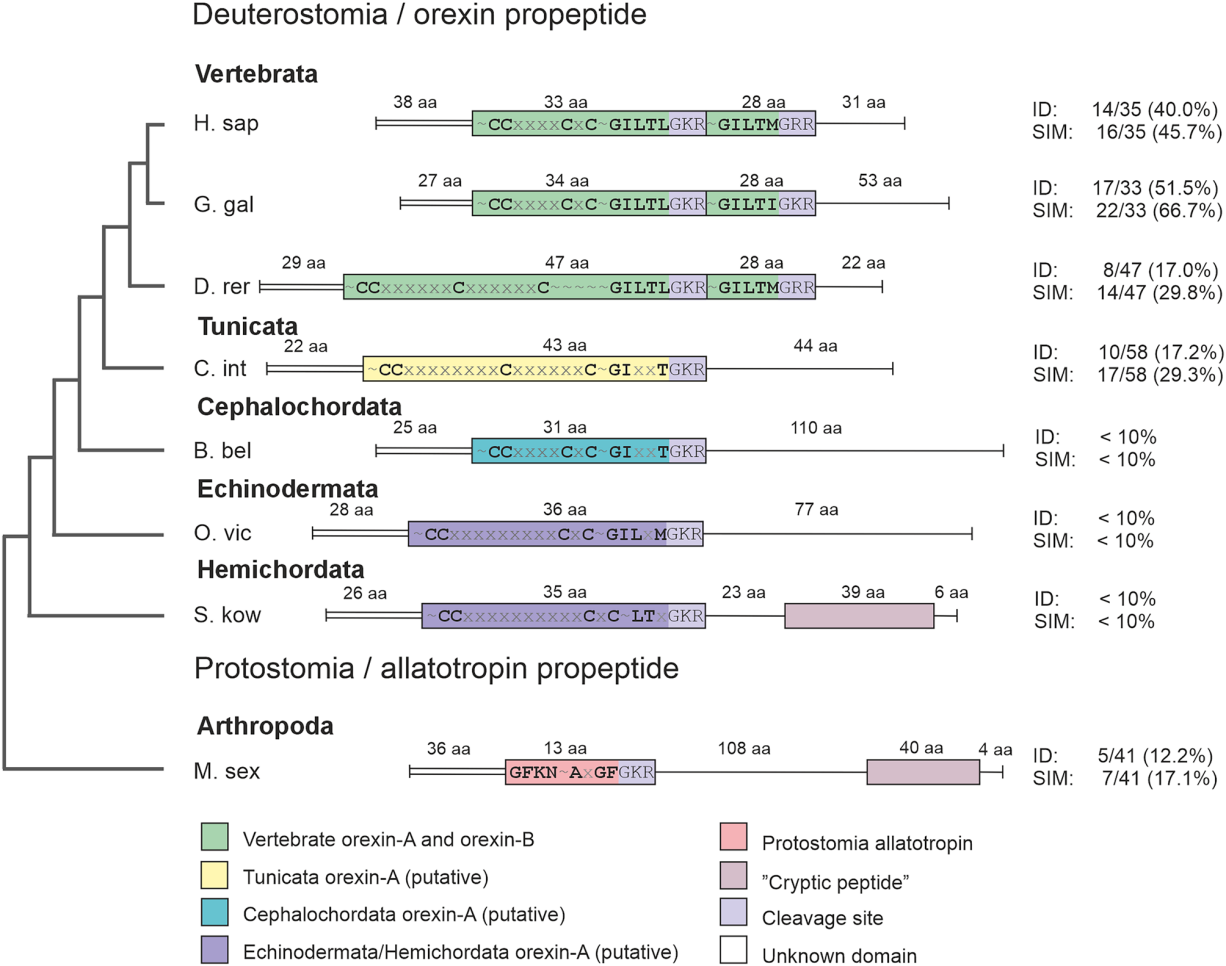
Prepro-orexin peptides from different species. For the species codes (Supplementary Table S1), the sequences used and the alignment (Supplementary Fig. S6), see Supplementary material 1. Left, a schematic presentation of the phylogenetic tree of the taxa shown. Boxes, coding/mature regions of the peptides (both orexin-A and -B in vertebrates and a single peptide in invertebrates and early vertebrates). Black lettering indicates conserved motives, ~ denotes region of length not specified here, x denotes region with a length specified here (number of x:s' = number of aa). Colour coding as in Fig. 1. Parallel horizontal double line, signalling peptide; vertical end of the line (prepro-peptide), stop. Right, percent identity (ID) and similarity (SIM) between orexin-A and orexin-B (vertebrates), orexin-A and the sequence directly downstream that would correspond to orexin-B (tunicates, cephalocordates, echinoderms), orexin-A and “cryptic peptide” (hemichordates), or allatotropin and “cryptic peptide” (protostome) within the individual species calculated by EMBOSS Needle, BLOSUM62 global alignment. Abbreviations: aa, amino acids.

The *C. intestinalis* putative prepro-orexin (CiPPO) is an orphan open reading frame named XM_026835150 (NCBI; reading frame 2). The region encoding Ci-orexin-A shares 22.2% sequence identity and 31.1% sequence similarity with human orexin-A (EMBOSS Needle, default parameters). Comparing vertebrate species, orexin-A and orexin-B peptides share one to another 17–56% identity, and are located in tandem in a single gene, which suggests that they have resulted from an internal gene duplication (Fig. 3, more comprehensive in Supplementary Fig. S5). Ci-orexin-A was named according to the vertebrate orexin-A since this segment of CiPPO also contains four cysteines with potential to form disulphide bridges whereas vertebrate orexin-B contains no cysteines (Supplementary Fig. S6). When comparing the putative orexin-A and the following region (corresponding to orexin-B in vertebrates) outside vertebrates, peptides do not share more identity than random sequences (≤ 10%; Fig. 3). A “cryptic peptide”^14^ has been identified in the genes coding for invertebrate allatotropin and the orexin-like peptide of *S. kowalevskii*, but such is not found in other (putative) orexin peptide sequences (Fig. 3).

**Figure 4:**
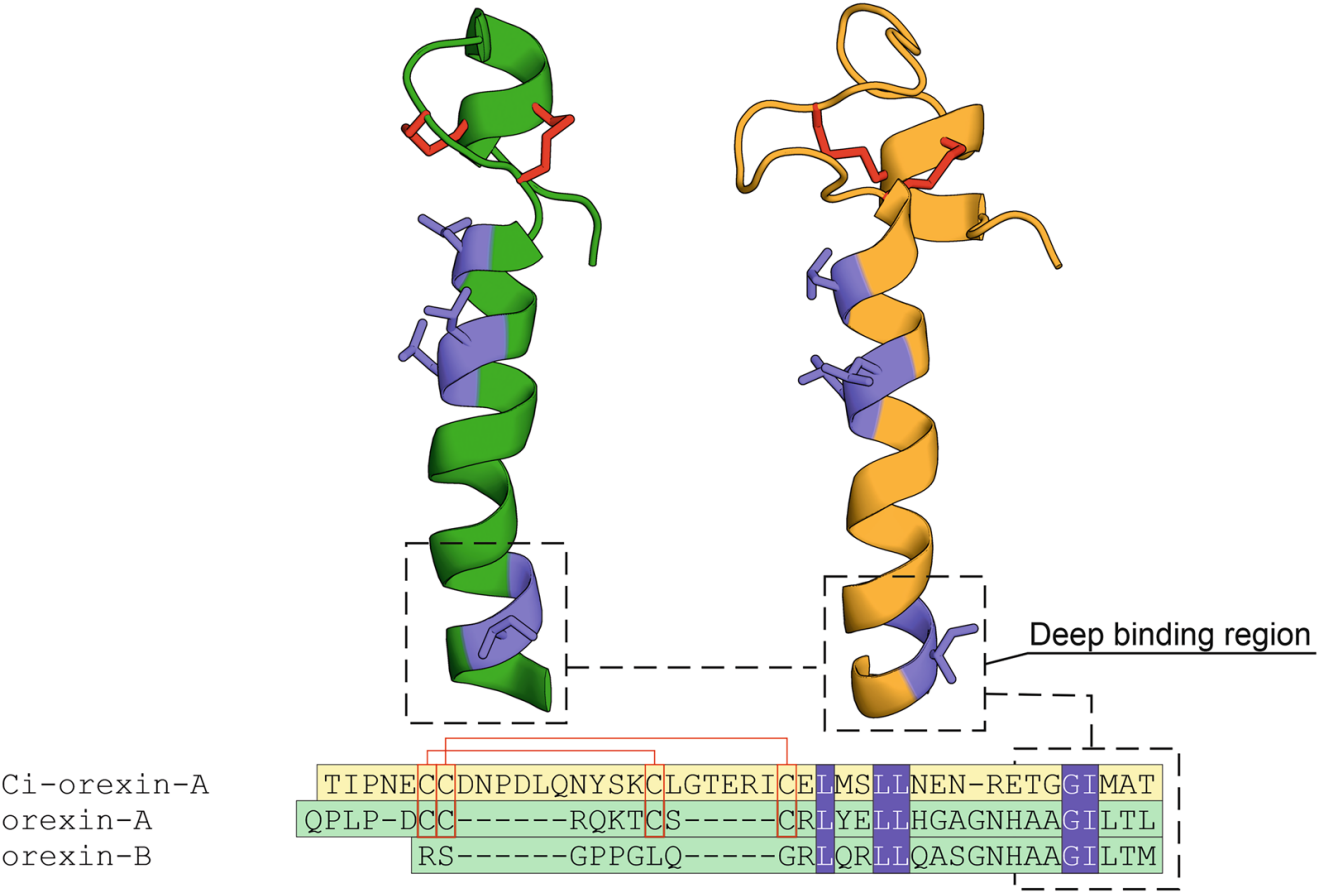
Comparison of human and *C. intestinalis* orexin peptides. Left, an NMR solution structure of human orexin-A (dark green; PDB code: 1WSO); and right, a homology model of Ci-orexin-A (orange). Below, a sequence alignment of human orexin-B, orexin-A and Ci-orexin-A. Disulphide bridges are shown as red sticks in the models and red lines in the sequence alignment. Identical residues are represented as blue sticks and blue boxes. Alignment was manually revised to match cysteines in orexin-A and Ci-orexin-A.

In tetrapods, disulphide bridges are formed between C6–C12 and C7–C14 of orexin-A. In ray-finned fishes, orexin-A peptides also have four cysteines in their N-terminus and thus should be able to form two disulphide bridges (Fig. 3). However, Xu and Volkoff^30^ have suggested, based on molecular modelling, that only the cysteine bridge C7–C14 is formed in ray-finned fishes, while C6 is unbridged. Ci-orexin-A has cysteines in positions 6, 7, 18 and 25. If both disulphide bridges are formed in the same way as suggested for tetrapod orexin-A, a remarkably long loop and a shorter one would be present in the N-terminal domain of Ci-orexin-A, yet these loops would not impact the deep binding region of the peptide (Fig. 4).

### CiOX receptor binds both TAMRA-orexin-A and Ci-orexin-A

Both hOX_1_-eGFP and CiOX-eGFP were well expressed on the surface of the stable clones of Flp-In T-REx 293 cells (green fluorescence in Supplementary Fig. S7 and S8). TAMRA-orexin-A (30 nM) bound to the plasma membranes of OX_1_ cells quickly after the addition (red fluorescence in Supplementary Fig. S7). We then tested the same for CiOX-eGFP-expressing cells: TAMRA-orexin-A bound to the plasma membranes of the CiOX receptor-expressing cells in an equally fast manner as to the OX_1_ receptor-expressing cells and the intensity remained stable for at least 30 min (red fluorescence in Supplementary Fig. S8). In contrast, no green fluorescence (eGFP channel in Supplementary Fig. S9) was observed in wild-type Flp-In T-REx 293 cells and TAMRA-orexin-A did not bind to them (TAMRA channel in Supplementary Fig. S9).

Four Ci-orexin-A variants were obtained through custom synthesis. All four peptides were C-terminally amidated. Two full peptides of 43 amino acids were additionally acetylated or not acetylated in the N-terminus (Ci-orexin-A and Ac-Ci-orexin-A), and two short variants containing 18 C-terminal amino acids, additionally acetylated or not acetylated in the N-terminus (Ci-orexin-A_26–43_ and Ac-Ci-orexin-A_26–43_). No disulphide bridges were introduced during the synthesis process of these peptides but the potential spontaneous formation of disulphide bridges in the peptides was also not assessed. The reduction of disulphide bridges in human orexin-A decreases its potency on hOX_1/2_, but the peptide remains active^31^ (Rinne & Kukkonen, unpublished data). Additionally, there are several possibilities for combinations of different disulphide bridges, thus testing all possible variants would have been extremely costly.

**Figure 5:**
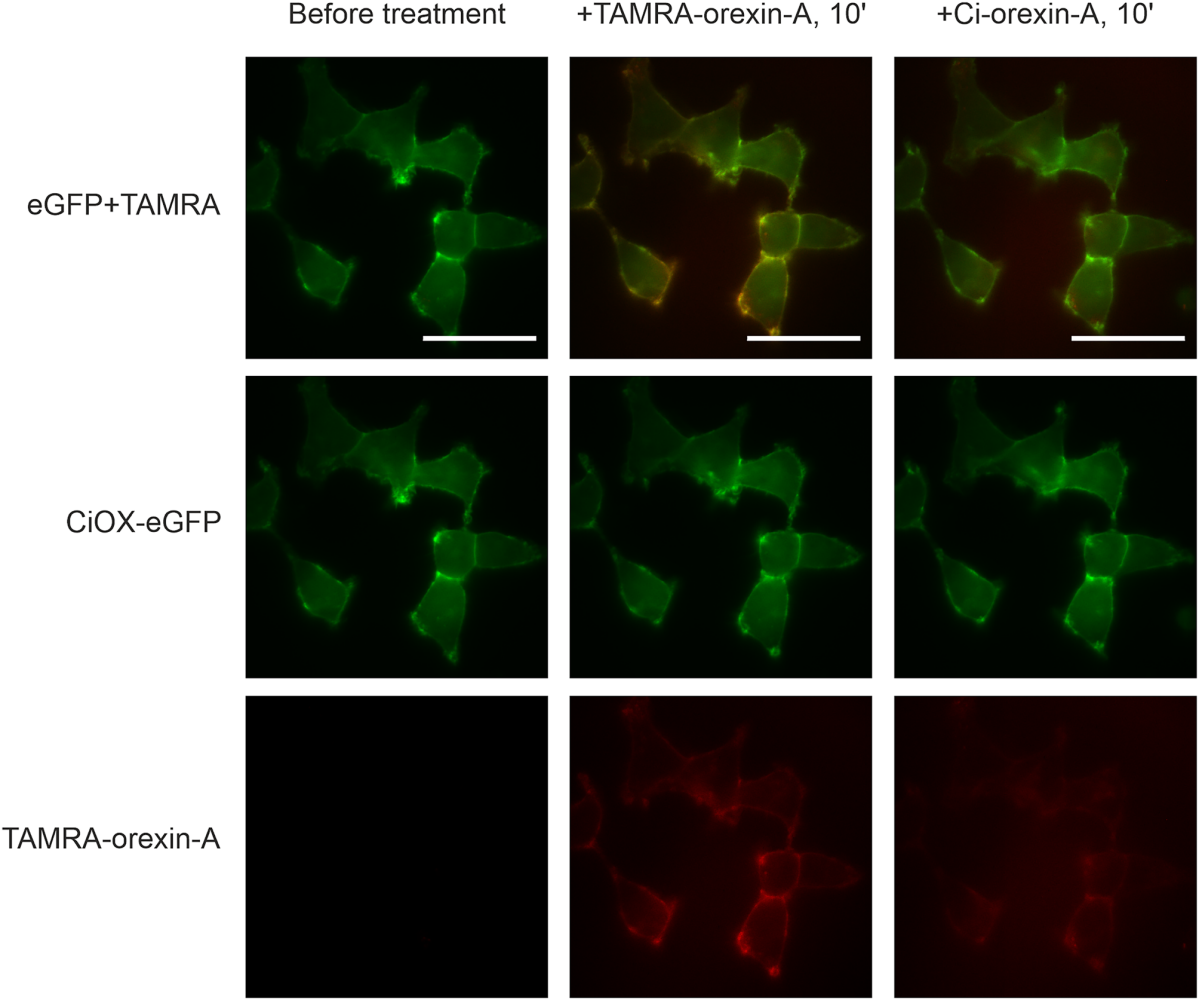
Binding of TAMRA-orexin-A to the plasma membrane and displacement by Ci-orexin-A(*1*) in CiOX-eGFP cells. Left, the cells before additions; middle, the cells after a 10 min incubation with 10 nM TAMRA-orexin-A; right, the cells after a further 10 min incubation with 1 µM Ci-orexin-A(*1*). Scale bar = 50 µm.

We then tested the competition of Ci-orexin-A(*1*) (1 μM) with TAMRA-orexin-A in CiOX-expressing cells. A 30-min preincubation with Ci-orexin-A(*1*) (1 μM) completely blocked TAMRA-orexin-A binding (Supplementary Fig. S10). In the same manner, adding Ci-orexin-A(*1*) (1 µM) after incubation with TAMRA-orexin-A displaced TAMRA-orexin-A (Fig. 5), indicating that Ci-orexin-A is able to bind to CiOX. The other variants of Ci-orexin-A(*2*–*4*) were not tested for CiOX binding.

For the hOX_1_ receptor-expressing cells, we also observed intracellular red fluorescence suggesting internalization of TAMRA-orexin-A probably together with the receptors (Supplementary Fig. S7). In contrast, we did not observe this in CiOX receptor-expressing cells (Supplementary Fig. S8).

### Human orexin peptides but not Ci-orexin-A activate CiOX

**Figure 6:**
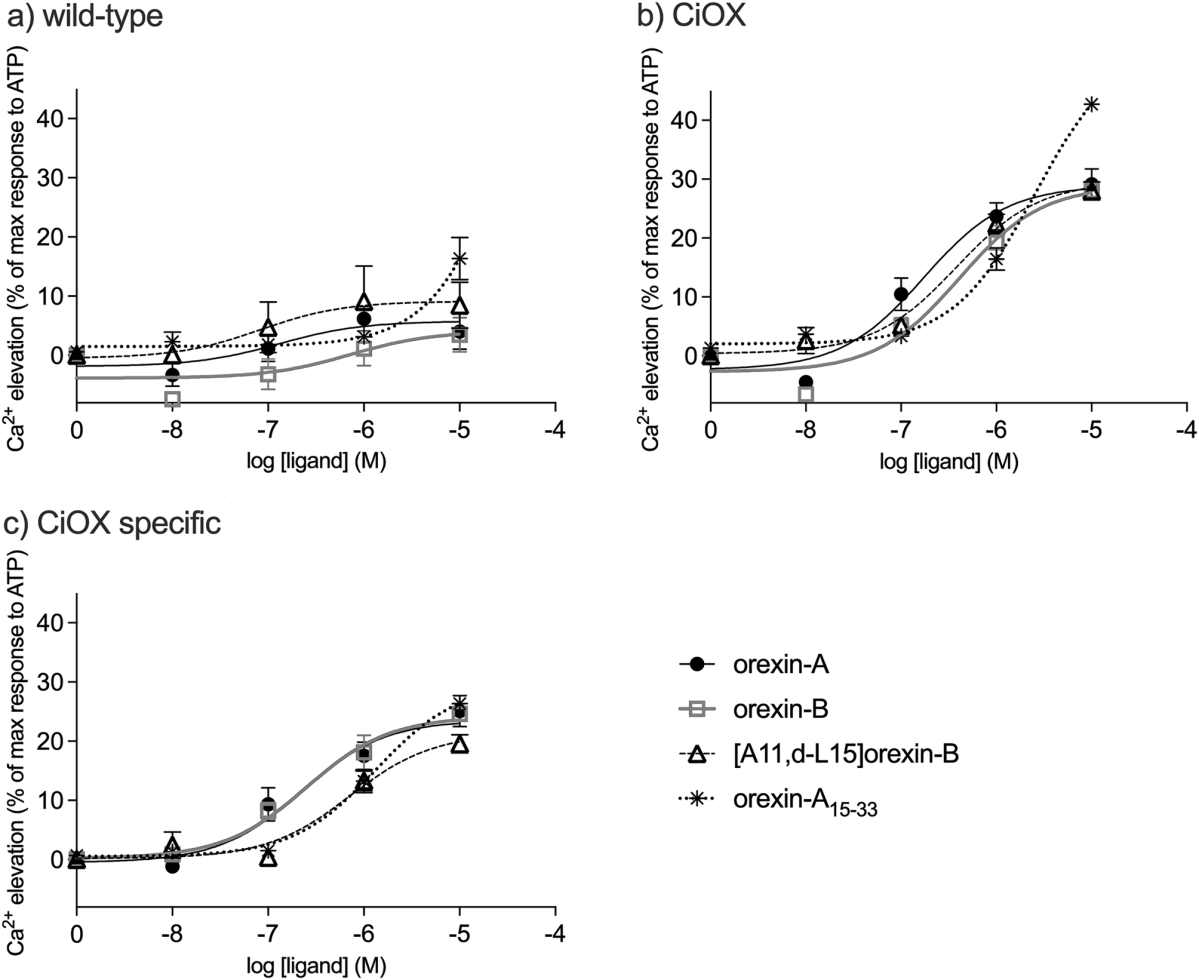
Ca^2+^ responses to receptor stimulation. Concentration–response curves for orexin-A, orexin-B, [A11, d-L15]orexin-B and orexin-A_15–33_ in (**a**) wild-type and (**b**) CiOX-expressing cells. In (**c**), CiOX-specific response, obtained by subtracting the response in wild-type cells from the response in CiOX-expressing cells. Responses are presented as normalized to the maximum ATP (100 µM; 100%) response separately for each independent sample to allow comparison between cell types. Data presented as mean ± S.E.M. N = 3.

Orexin-A and orexin-B, endogenous ligands of hOX_1/2_, and ATP (an endogenous ligand for endogenous P2 receptors) induced strong, concentration-dependent intracellular Ca^2+^ elevations in hOX_1_ and hOX_2_ receptor-expressing CHO-K1 and HEK293 cells (see, e.g. ^32^) and we have seen the same in Flp-In T-REx 293 cells (Rinne & Kukkonen, unpublished; see also Supplementary Fig. S11). We tested here the effect of the hOX_1/2_ agonists on wild-type and CiOX-eGFP-expressing Flp-In T-REx 293 cells. All human orexin peptide ligands (orexin-A, orexin-B, [A11, d-L15]orexin-B and C-terminal fragment of orexin-A (19 amino acid; orexin-A_15–33_)) weakly elevated Ca^2+^ in the wild-type cells (Fig. 6a) via either endogenous orexin receptors or some other target, however, the maximum elevations were not significant when compared to basal (P value > 0.01). Luckily, the Ca^2+^ elevation was clearly larger in CiOX-eGFP-expressing cells (Fig. 6b). The specific response (derived by subtracting the response in wild-type cells from that in CiOX cells) amounted to about 20% of the response to 100 µM ATP (Fig. 6c). EC_50_-values were as follows: orexin-A (224.7 ± 8.1 nM), orexin-B (250.3 ± 6.6 nM), and [A11, d-L15]orexin-B (715.8 nM ± 20.5 nM); no saturation was reached with orexin-A_15–33_, and thus its EC_50_ value could not be determined.

In contrast, none of the four Ci-orexin-A peptides induced any Ca^2+^ elevation in CiOX receptor-expressing cells (not shown). We additionally tested the potent and efficacious small molecule agonist of human orexin receptors, Nag 26^33,34^, and the *M. sexta* allatotropin but these induced no Ca^2+^ elevation either in CiOX or wild-type cells.

**Figure 7:**
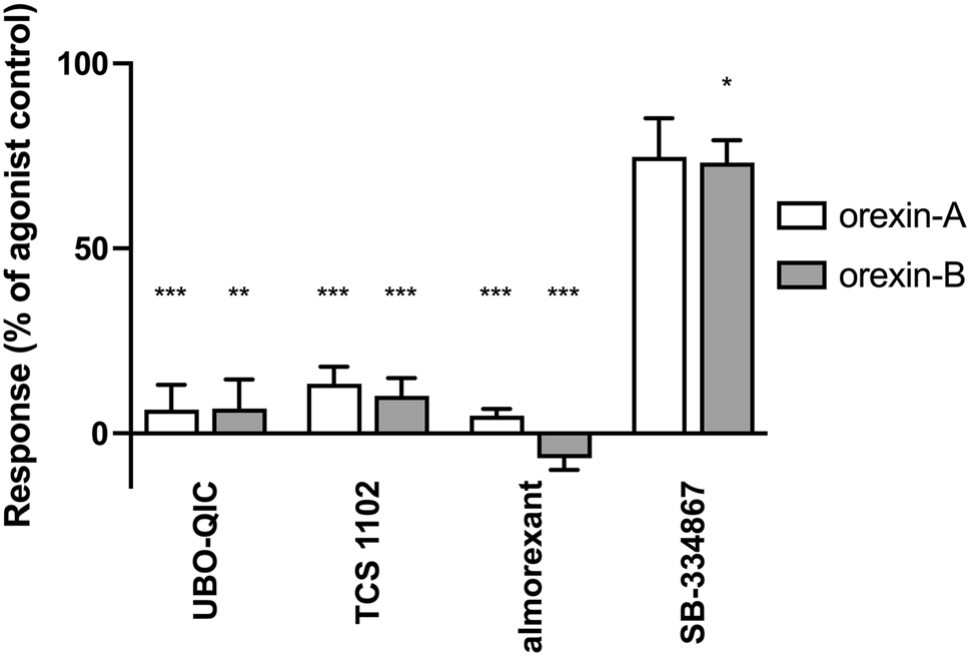
The sensitivity of the human endogenous orexin peptide (1 µM) -mediated Ca^2+^ responses to inhibitors (1 µM) in CiOX-expressing cells. The responses were normalized to the basal + inhibitor (0%) and the control agonist peptide response (in the absence of inhibitor; 100%) separately for each independent experiment before averaging. Data presented as mean ± S.E.M. N = 3–4. The significances are given in relation to the agonist control.

The Ca^2+^ responses to CiOX stimulation with orexin-A and -B (1 µM) were nearly fully inhibited by the G_q/11/14_ inhibitor UBO-QIC (1 µM). The non-selective human orexin receptor antagonists TCS 1102 and almorexant (1 µM) also nearly fully blocked the response, whereas the hOX_1_-selective antagonist SB-334867 (1 µM) had a much weaker effect (Fig. 7).

### Modelling of the peptide–CiOX complexes

To gain a structural understanding on the binding of orexin-A and Ci-orexin-A to CiOX, we used a cryo-electron microscopy (cryo-EM) structure of hOX_2_ in complex with orexin-B and a G protein to build homology models of human orexin-A–CiOX and Ci-orexin-A–CiOX complexes. Orexin-A and orexin-B differ only at the terminal residue (L33 in orexin-A, M28 in orexin-B) in the segment of orexin-B that is resolved in the cryo-EM structure, which suggests that binding interactions in this segment are conserved among the two peptides.

**Figure 8:**
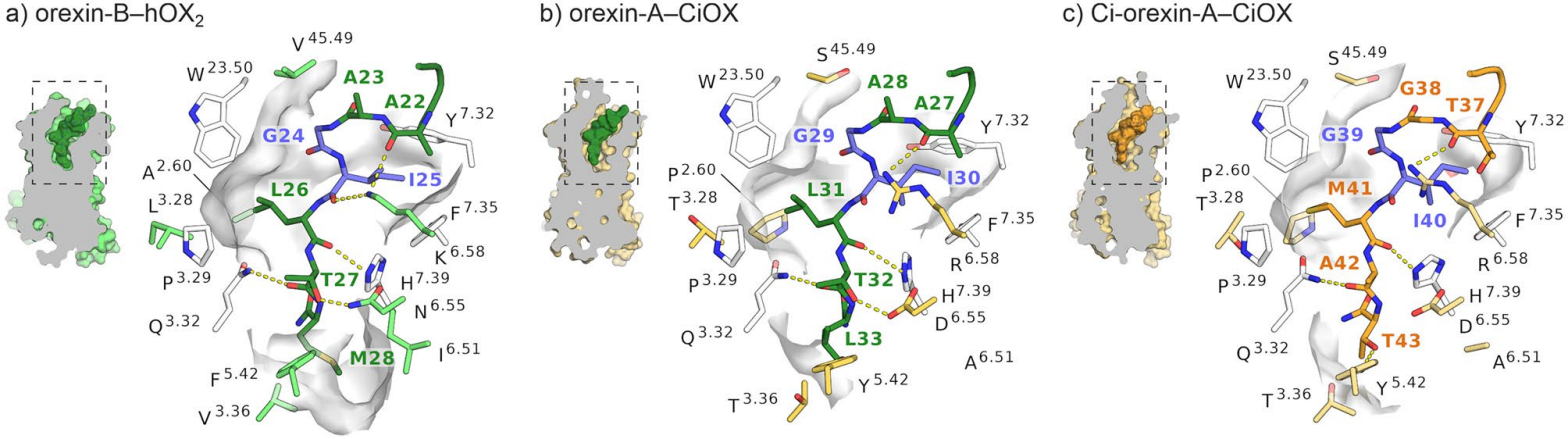
Comparison of the binding interactions of human and *C. intestinalis* orexin peptides. (**a**) Cryo-EM structure of human orexin-B (dark green) bound to hOX_2_ (light green) (PDB ID: 7L1U), (**b**) homology model of the putative binding mode of human orexin-A (dark green) to CiOX (bleak yellow), and (**c**) homology model of the putative binding mode of Ci-orexin-A (orange) to CiOX (bleak yellow). Binding site side chains conserved between hOX_2_ and CiOX are shown as uncoloured sticks. Residues not conserved are shown in light green (hOX_2_) and bleak yellow (CiOX). Residues conserved between human and *C. intestinalis* orexin peptides are shown as blue sticks.

In the orexin-B–hOX_2_ structure, residues N20–M28 of the C-terminal part of orexin-B are resolved in an extended conformation, with the C-terminus inserted deep in the core of the receptor (Fig. 8a). Residues G24, I25, L26 and M28 of orexin-B provide high shape-complementarity with the binding cavity, while key polar interactions are formed by backbone oxygen atoms and the sidechain hydroxyl of T27. In detail, the main chain α-carbon of G24 is packed against V^ECL2.49^ in ECL2 of hOX_2_, the side chain of I25 is sandwiched between the aromatic rings of Y^7.32^ and F^7.35^ in TM7, while the side chain of L26 occupies a hydrophobic pocket between TM2 and TM3 formed by P^3.29^, L3.28, W^ECL1.50^ and A^2.60^. In the bottom of the binding cavity, M28 is surrounded by the side chains of F^5.42^, I^6.51^ and V^3.36^. In addition to these hydrophobic contacts, hydrogen bonds are formed to K^6.58^, H^7.39^, Q^3.32^ and N^6.55^ of hOX_2_.

Analysis of the homology models shows that the interactions in the binding cavity would be mostly preserved between the ligand–receptor pairs of orexin-B–hOX_2_ and orexin-A–CiOX. In the orexin-A–CiOX model (Fig. 8b), G29, I30, and L31 of orexin-A form hydrophobic contacts to ECL2, TM7, and the TM2–TM3 pocket, respectively, while hydrogen bonds are formed to R^6.58^, H^7.39^, Q^3.32^ and D^6.55^ of CiOX, which are equivalent to the positions in hOX_2_ (Fig. 8a). The main difference in the binding interactions is found in the bottom of the cavity, where F^5.42^ and V^3.36^ of hOX_2_ (Fig. 8a) are substituted for Y^5.42^ and T^3.36^ in CiOX (Fig. 8b), introducing a slightly more polar environment in the vicinity of L33 of orexin-A. Importantly, in the modelled active CiOX conformation, the distance between the hydroxyl groups of Y^5.42^ and T^3.36^ (> 5 Å) does not permit a hydrogen bond interaction.

In the Ci-orexin-A–CiOX model, Ci-orexin-A retains the hydrophobic contacts to ECL2 and TM7 with the conserved G-I motif (Fig. 4) and occupies the TM2–TM3 pocket with M41. The hydrogen bonds formed between CiOX and the peptide backbone oxygen atoms are equivalent to those in orexin-A–CiOX and orexin-B–hOX_2_ complexes, but the hydrogen bond to D^6.55^ is absent in Ci-orexin-A–CiOX as Ci-orexin-A contains A42 (Fig. 8c). The corresponding position in the human orexin peptides contains a threonine (connecting to N^6.55^ in hOX_2_ or D^6.55^ in CiOX; Fig. 8a,b, respectively). Ci-orexin-A has T43 as the C-terminal residue, which is positioned well to form a hydrogen bond with Y^5.42^ in the bottom of the CiOX binding cavity (Fig. 8c).

## Discussion

In this study, we expressed and characterized a putative *C. intestinalis* orexin receptor in human cells. CiOX is slightly closer to hOX_1/2_ than to ATRs in terms of sequence conservation. In contrast, in the phylogenetic analyses, the orexin receptors in vertebrates and cephalochordates are positioned in five out of six trees closer one to another in comparison with CiOX from tunicates. This is unexpected because vertebrates and tunicates are believed to be evolutionary closer than vertebrates and cephalochordates^35^. Importantly, some of the bootstrap values are low (< 65%), indicating a lack of robustness in the position of the corresponding branches, and the CiOX clade is characterized by the longest branches, indicating high degree of divergence. This would suggest that tunicate receptors have diverged at a faster rate, which would explain why they branch further away from their expected positions. This could be explained by the high amino acid substitution rate in *C. intestinalis*^9^.

The data presented in this study strongly support the notion that this orphan *C. intestinalis* receptor, here called CiOX, is an orexin-like receptor. Despite the sequence divergence, the (proposed) binding cavity of hOX_2_ and CiOX are highly similar. The homology models of CiOX suggests that the amino acid residues that are crucial for binding are conserved throughout the species, and located spatially in a way compatible with a key role of the peptides’ C-terminus. Experimentally, we demonstrate that the receptor is well expressed on the surface of human cells and binds TAMRA-labelled human orexin-A. The receptor couples in some degree to human G_q_ as demonstrated by the sensitivity of the Ca^2+^ responses triggered by the human orexin peptide variants orexin-A, orexin-B, [A11, d-L15]orexin-B and orexin-A_15–33_ to the G_q/11/14_ inhibitor UBO-QIC. These Ca^2+^ responses were also inhibited by the mammalian orexin receptor antagonists TCS 1102, almorexant and SB-3348967. In contrast, the small molecule hOX agonist Nag 26 did not induce any calcium response. The data further support the concept of the similarity between the binding site of the CiOX and the hOX_1/2_. Very few compounds have been reported to tested on both orexin and allatotropin receptors, and, to our knowledge, testing mammalian orexin peptides in allatotropin receptors has not been reported in the literature. However, allatotropin receptor of *Tribolium castaneum* was not inhibited by almorexant^36^.

We also aimed at identifying the endogenous *C. intestinalis* prepro-orexin peptide; to our best knowledge, a candidate orexin peptide in tunicates has not been proposed before. The identified sequence (CiPPO) harboured a potential peptide that we named Ci-orexin-A based on the presence of a cysteine-containing N-terminus; neither orexin-B equivalent nor cryptic peptide as in the PPO of *S. kowalewskii* and in the allatotropin propeptides were found. We could see that the full-length synthetic Ci-orexin-A(*1*) competed with TAMRA-orexin-A for the binding to CiOX, suggesting that it binds to CiOX. We tested the four synthetic variants of this putative orexin-A sequence (Ci-orexin-A variants), but no Ca^2+^ elevation was seen upon stimulation of the CiOX receptor with any of the peptides. A reasonable doubt remains as to whether the correct endogenous peptide has been identified, including its cleavage sites (particularly in N-terminus), posttranslational modifications, as well as whether the formation of disulphide bridges should have been controlled. It should also be noted that other candidate peptides, if they exist, may be identified in the future using data mining techniques tailored for retrieving short peptides.

Another explanation for the lack of efficacy of CiOX may lie in potentially different proteins needed for orexin receptor signalling among human and tunicates. It is thus possible that the signal transduction machinery of mammalian cells is less optimal for CiOX. Finally, we must consider the possibility that CiOX, unlike its mammalian orthologs, may rather signal via other pathways than G_q_, as we did not investigate other known GPCR signal transduction pathways. Although human orexin receptors couple to Ca^2+^elevation presumably via G_q_, in basically every cell type tested ^1^, it is not said that their distant relative, CiOX, couples in the same manner. However, when expressed alone or together with the promiscuous G_16_-protein, insect ATRs couple to Ca^2+^ elevation in mammalian cells^37^. Further studies of the CiOX signalling are thus required^37–39^.

The evolutionary events that led to the emergence of the ATR, the CiOX, and the vertebrate OX_1_ and OX_2_ receptors are blurry. The data presented in this study, together with the compelling evidence for two rounds of whole genome duplication in the lineage leading to vertebrates^38–40^, can be reconciliated into a simple evolutionary hypothesis: a single orthologous gene would encode for the allatotropin receptors in protostome and the orexin-like receptors in tunicates. The ability to be activated by the long orexin-like peptides instead of allatotropin peptides would have emerged before the split between tunicates and vertebrates through point mutations. As shown in this study, about 16 point mutations at the binding site may explain the binding specificity for allatotropin and orexin ligands at least in the deep-binding region. Following this split, the CiOX would have diverged at high rate, and the OX_1_ and OX_2_ receptors would have emerged from the vertebrate whole-genome duplication. Two hypotheses are plausible: (a) either one the ancestral OX genes has been inactivated after the first round, or (b) two of the four duplicated orexin receptors has been lost after the second round^38,41^. There is no evidence of whole genome duplications in the *C. intestinalis* genome, which harbours approximately 16,000 predicted genes, including 169 suggested GPCR genes^42^*.* While this hypothesis is parsimonious, it does not exclude other scenarios^21,41^. More studies, in particular gene synteny analysis, could give further insights into evolutionary relationships of genes between different organisms. It should however be noted that *C. intestinalis* has reduced gene synteny conservation^9^, which makes this analysis challenging or even unfeasible.

The orexin system has a central role in many physiological functions in mammalians (see Introduction). In the allatotropin system, first identified in *M. sexta*, regulates the production of juvenile hormones, modulates the circadian clock and the myotropic activity, and has role also in feeding^37,43,44^. Thus, there is evidence for similar roles of the orexin and allatotropin systems, at least in the regulation of circadian activities and feeding. The function of the potential orexin system in tunicates is unknown, but it is not unreasonable to assume a function along similar lines.

Altogether, these data demonstrate that CiOX is an orexin receptor-like receptor, an evolutionary distant relative of both the OX_1_ and OX_2_ receptors. Furthermore, we propose that Ci-orexin-A binds to hOX_1_ and CiOX and affects CiOX, although its actual sequence and post-translational modifications are uncertain.

## Methods

### Molecular phylogeny and three-dimensional structure modelling

The CiOX is encoded by the Ensembl gene ENSCING00000007467^17^ (accessed 17^th^ July 2023). Two transcripts may be produced from this gene, X1 and X2, which differ in their C-terminus. The X2 transcript (NCBI transcript XM_002127151.3; NCBI protein XP_002127187.1^46^ and Uniprot F6YII5_CIOIN^45^; accessed 17th July 2023) was obtained from the mRNA of animal tissue—a strong indication that this variant actually exists in nature—and cloned to the RIKEN cDNA library. Only the X2 transcript was known when this study started.

The phylogenetic relationships of CiOX with vertebrate orexin receptors and ATRs was investigated using the MEGA X package^47^. The analysis included retrieval of orexin and ATR amino acid sequences for organisms of different orders from the NCBI peptide database. This data set was supplemented with four human neuropeptide receptor sequences to root the tree (NPFF1 neuropeptide FF receptor, GAL_2_ galanin receptor, QRFP pyroglutamylated RFamide peptide receptor, and ET_B_ endothelin receptor). The initial alignment produced by MEGA X was manually revised to align GPCR family-specific amino acids and to avoid gaps in TM (sequences and alignments are in Supplementary material 1–3). The phylogenetic analyses were conducted either with full sequences (599 positions, out of 798 possible positions in the alignment that did not contain many gaps) or restricted to the regions corresponding to TMs (231 positions out of 249). Three different tree construction methods were compared: minimum evolution (Poisson model), maximum likelihood (Jones–Taylor–Thornton model) and neighbour-joining (Poisson model). Five hundred bootstrap replicates were conducted in all cases.

Homology modelling of the CiOX receptor was conducted using the high-resolution crystal structure of hOX_2_ receptor (PDB code: 5WQC^22^). Regions absent from template structure, such as termini and intercellular loop 3, were not modelled. Five models were constructed using the MODELLER software v9.22 with default settings^48^, and the model with the best discrete optimized protein energy (DOPE) score selected for further analysis. The well-defined binding cavity of the crystal structure of hOX_2_—suvorexant complex (PDB code 4S0V^25^) was used for the definition of the CiOX binding pocket: residues with atoms within 5 Å of suvorexant, complemented with residues forming the 4 salt bridges in the hOX_2_'s binding site.

Discovering a hit for the CiPPO proved to be challenging. We aimed to find an unassigned open reading frame for a PPO peptide containing more than 100 amino acids, the cysteine cap (pattern CCx_n1_Cx_n2_C), and the cleavage site (ILTL/GKR) characteristic of the vertebrate orexin-A peptide. Database searches were conducted using BLAST to query the Ensembl database (release 95)^49^. Several vertebrate and invertebrate PPO segments coding the signal peptide, orexin-A and the cleavage site were queried against *C. intestinalis* and *Ciona savignyi* cDNA using TBLASTN (BLAST of protein sequence on a nucleotide database); Blosum45, distant homologies, gap penalties 10 for opening and 3 for extension, low complexity regions filtered out). The initial hit was identified in *C. savignyi* cDNA using zebrafish (*Danio rerio*) PPO segment as a query. The initial hit was further queried against *C. intestinalis* cDNA and the final hit identified as an uncharacterized protein with mRNA evidence in NCBI; code: XM_026835150, reading frame 2, location in LOC113474376, chromosome 7 (accessed 17th July 2023).

Following discovery of a suitable open reading frame, we analysed its putative 3D structure by homology modelling. A three-dimensional model of the Ci-orexin-A was constructed using the human orexin-A peptide (PDB code: 1WSO^50^) as the template structure. The presence of a potential signal peptide was predicted with SignalP-5.0 online tool (likelihood 0.9995)^51^. One hundred homology models were constructed in order to cover multiple conformations, and one with the highest DOPE score selected for further analysis.

To model the structures of orexin-A and Ci-orexin-A in complex with CiOX, we used the active-state cryo-EM structure of hOX_2_ bound to orexin-B (PDB ID: 7L1U^24^) as a template. This structure was solved while the study was under way. N- and C-termini, as well as intracellular loop 3, which were absent in the template structure, were removed from the target sequence. A total of 100 models of both orexin-A—CiOX and Ci-orexin-A—CiOX complex models were built using MODELLER 10.1 with default settings, and one with the highest DOPE scores selected for analysis.

Visualization and figure preparation were performed with PyMOL^52^ and Gimp 2.10.14 GIMP (http://www.gimp.org, RRID:SCR_003182). The alignment figures were created with Jalview^53^.

### Materials

Human orexin-A, orexin-B and [A11, d-L15]orexin-B were purchased from NeoMPS (Strasbourg, France), orexin-A_15–33_ from GenScript (Nanjing, China), the putative *C. intestinalis* orexin peptide (and variants) and the human TAMRA-orexin-A from Peptide Specialty Laboratories GmbH (Heidelberg, Germany), *M. sexta* allatotropin from Bachem (Bubendorf, Switzerland), TCS 1102 ((2*S*)-1-[2-(1-methylbenzimidazol-2-yl)sulfanylacetyl]-*N*-(2-phenylphenyl)pyrrolidine-2-carboxamide) and SB-334867 from Tocris Bioscience (Bristol, UK). Almorexant ((2*R*)-2-[(1*S*)-6,7-dimethoxy-1-[2-[4-(trifluoromethyl)phenyl]ethyl]-3,4-dihydro-1*H*-isoquinolin-2-yl]-*N*-methyl-2-phenylacetamide) was a gift from Actelion Pharmaceuticals Ltd. (Allschwil, Switzerland), Nag 26 (*N*-[2-[3-[[5-[3-(dimethylcarbamoyl)phenyl]-2-methoxyphenyl]sulfonylamino]anilino]ethyl]-3-methylbenzamide) was synthetized at the University of Helsinki)^33^, and UBO-QIC/FR900359 ([(1*R*)-1-[(3*S*,6*S*,9*S*,12*S*,18*R*,21*S*,22*R*)-21-acetamido-18-benzyl-3-[(1*R*)-1-methoxyethyl]-4,9,10,12,16-pentamethyl-15-methylidene-2,5,8,11,14,17,20-heptaoxo-22-propan-2-yl-1,19-dioxa-4,7,10,13,16-pentazacyclodocos-6-yl]-2-methylpropyl] (2S,3*R*)-3-hydroxy-4-methyl-2-(propanoylamino)pentanoate) was from the Institute of Pharmaceutical Biology, University of Bonn (Bonn, Germany).

### Cell culture

The Flp-In T-REx 293 human cell line (Thermofisher Scientific, Waltham, MA, USA), based on HEK293 cells, was cultured at 37 °C in 5% CO_2_ in an air‐ventilated humidified incubator according to manufacturer’s protocol, in high glucose Dulbecco’s Modified Eagle Medium (Biowest, Nuaillé, France) supplemented with 10% FBS, 1% Glutamax (Gibco), 10 mM HEPES (VWR LifeScience, Radnor, PA, USA), 100 U/mL penicillin G (Sigma Chemical Co., St. Louis, MO, USA), 80 U/mL streptomycin (Sigma), blasticidin (15 µg/mL) (InvivoGen, San Diego, CA, USA). The host cell line was maintained with zeocin (100 µg/mL) (InvitroGen, Carlsbad, CA, USA) and the transfected cell lines with hygromycin B (100 µg/mL) (InvitroGen). The experimental medium, the HEPES-buffered medium, (HBM: 137 mM NaCl, 5 mM KCl, 1.2 mM MgCl_2_, 0.44 mM KH_2_PO_4_, 4.2 mM NaHCO3, 1 mM CaCl_2_, 10 mM glucose, 20 mM HEPES, adjusted to pH 7.4 with NaOH), was used in all laboratory experiments. For the calcium elevation assay, HBM was supplemented with 1 mM probenecid [4-(dipropylsulfamoyl)benzoic acid] (Sigma) and 0.2% lipid-free bovine serum album.

### Plasmids and cloning

The cDNA of CiOX (transcript variant X2) in the pBluescriptII SK(–) vector (https://dna.brc.riken.jp/en/cloneseten/ciona_est_en) was obtained from RIKEN BioResource Research Center (Tsukuba, Japan). The stop codon was removed by polymerase chain reaction and the enhanced green fluorescent protein (eGFP) coding sequence was fused to the 3'-end of the receptor coding sequence. hOX_1_ was similarly fused to eGFP as described previously^54^. The CiOX-eGFP and hOX_1_-eGFP constructs were transferred into the mammalian expression vector pcDNA5/FRT/TO (Invitrogen/ThermoFisher Scientific, Waltham, MA, USA). These constructs allow stable tetracycline-inducible mammalian expression of CiOX and hOX_1_ receptors in Flp-In T-REx 293 cells; the C-terminal eGFP enables visualization of the expression levels and the subcellular localizations of the receptors. Plasmid modifications were planned with the software SerialCloner2.6^55^. *C. intestinalis* genetic code was not optimized for human cells as the receptor was well expressed as such in human Flp-In T-REx 293 cells (see later in Results).

### Generation of the stable cell lines

The stable cell lines were based on the Flp-In T-REx 293 cells (ThermoFisher) allowing flippase-mediated recombination with a single integrated flippase recognition target (FRT) site. Recombination with the insert in pcDNA5/FRT/TO generates stable cell lines with tetracycline-inducible expression. The host cells were separately transfected with the receptor plasmids pcDNA5/FRT/TO-CiOX-eGFP and pcDNA5/FRT/TO-hOX_1_-eGFP (+ the accessory plasmid pOG44) using GeneJuice transfection reagent (Merck, Darmstadt, Germany) and cultured and selected according to the manufacturers protocol. Four foci were picked for each cell type and tested for doxycycline-induced gene expression and zeocin-sensitivity. One focus was selected for each cell type for further studies. The doxycycline induced gene expression was further assessed by fluorescent microscopy (eGFP) after 24 h and 48 h from the treatment with different concentrations of doxycycline: 0, 0.1, 1, 10, 100 and 1000 ng/mL. 100 ng/mL was chosen for the induction of the expression in further studies, to ensure comparable expression in both cell lines. Differences in the apparent expression level were not observed between the timepoints 24 h and 48 h.

### Binding of TAMRA-orexin-A

We have previously observed that the N-terminus of orexin-A can tolerate additions without apparent loss of activity on orexin receptors (Kukkonen, unpublished) and it has been reported that fluorescently labelled orexin-A can be used as probe for hOX_1/2_^56^. We thus ordered custom synthesis of TAMRA-orexin-A. This peptide shows red fluorescence (555/580 nm), and could thus be used to show binding to the membranes and co-localization with the green fluorescent, eGFP-fused OX_1_ and CiOX receptors in microscopy.

The cells were seeded on polyethyleneimine-coated 25 mm circular glass coverslips (Menzel-Gläser, Braunschweig, Germany). Twenty-four hours after seeding, cells were treated with doxycycline (100 ng/mL) and incubated for another 24 h. Then, the coverslip was washed once with HBM, before mounting in the microfluorometric experiment chamber. The imaging system, composed of the Sutter Lambda DG-4 xenon light source/filter shifter (Sutter Instrument Company, Novato, CA, USA), Nikon TE2000 epifluorescence microscope (Nikon, Tokyo, Japan), Nikon Apo TIRF 60×/1.49 oil immersion objective and Andor iXon 885 electron-multiplying charge-coupled device camera (Oxford Instruments, Abingdon, Oxfordshire, UK), was under the control of the Nikon NIS Elements AR software. Images were alternately acquired at 480/30 nm excitation/510 nm dichroic mirror/535/40 nm emission for eGFP fluorescence and at 540/25 nm excitation/565 nm dichroic mirror/605/55 nm emission for TAMRA fluorescence. A pair of images was acquired once per minute or once per every five minutes depending on the incubation time; i.e., for a longer incubation 5 min was used to avoid potential bleaching. All the additions were made by direct pipetting into the chamber. Experiments were carried out at room temperature. 30 nM TAMRA-orexin-A was used.

### Calcium elevation

Intracellular calcium elevation is the apparent response upon orexin receptor activation in many cell types^1^. The calcium elevation assay was conducted as previously described^33^. Cells, 1 × 10^5^ per well, were plated on polyethyleneimine (25 μg/mL, 1 h, 37 °C)-coated black, clear-bottom, half-area 96-well plates (Greiner). After 24 h, 100 ng/mL doxycycline was added onto the cells and incubated for another 24 h before loading the cells with FLIPR Calcium 5 Assay Kit (Molecular Devices, Sunnyvale, CA, USA) dissolved in and diluted with HBM and supplemented as described above. Cells were incubated for 1 h with the loading dye, before running the assay in FlexStation 3 fluorescence plate reader (Molecular Devices). Inhibitors were added manually at the midpoint of the probe incubation (30 min). Intracellular Ca^2+^ levels were measured as fluorescent changes for 150 s (of which 30 s baseline prior to stimulation) with excitation at 485 nm, emission at 525 nm (recording every 1.3 s) at 37 °C. For the wild-type cells and CiOX-expressing cells, 100 µM ATP gives a robust Ca^2+^ response and thus it was used as a positive control.

### Data collection and analysis

Calcium measurements were performed in triplicate or quadruplicate. Microsoft Excel was used for calcium data visualizations and analyses. Non-linear curve-fitting were conducted with Prism 5 (GraphPad Software Inc., La Jolla, CA, USA) using the following equation:

$${\text{Y}}=\text{basal }+\frac{ \left({{\text{response}}}_{{\text{max}}}-{\text{basal}}\right)}{1+{10}^{\left({{\text{logEC}}}_{50}-{\text{X}}\right)}}$$

The data were normalized in each experiment to the control response and then averaged between experiments by calculation of mean ± S.E.M. The N denotes the number of independent experiments and was always at least 3. Paired two-tailed t-test was used for comparison. Significances were as follows: * P < 0.05, ** P < 0.01, *** P < 0.001.

### Supplementary Information

Supplementary Information 1.

Supplementary Information 2.

Supplementary Information 3.

## Data Availability

Data available on request from the corresponding authors Henri Xhaard or Jyrki Kukkonen.

## Abbreviations

Ac-Ci-orexin-A(*2*) and Ci-orexin-A(*1*): *C. intestinalis* Putative orexin-A with or without N-terminal acetyl group
Ac-Ci-orexin-A_26–43_(*4*) and Ci-orexin-A_26–43_(*3*): The 18-amino acid-long C-terminal fragment of Ci-orexin-A with or without N-terminal acetyl group
ATR: Allatotropin receptor
CiOX: *C. intestinalis* Putative orexin receptor
CiPPO: *C. intestinalis* Putative prepro-orexin
cryo-EM: Cryo-electron microscopy
DOPE: Discrete optimized protein energy
ECL: Extracellular loop
eGFP: Enhanced green fluorescent protein
GPCR: G protein-coupled receptor
HBM: HEPES buffered medium
hOX_1_ and hOX_2_: Human orexin receptors
orexin-A_15–33_: The 19-amino acid-long C-terminal fragment of orexin-A
PDB: Protein Data Bank
PPO: Prepro-orexin
TAMRA: 5-/6-Carboxytetramethylrhodamine
TM: Transmembrane segment
