# Supplementary material for: Characterization of a putative orexin receptor in Ciona intestinalis sheds light on the evolution of the orexin/hypocretin system in chordates
Source: Sci Rep. 2024 Apr 2;14:7690. doi: 10.1038/s41598-024-56508-1 (PMC10987541; doi:10.1038/s41598-024-56508-1)
Supplement: Supplementary file 1 — Supplementary Information 1. [file 41598_2024_56508_MOESM1_ESM.docx]

**Sequences of receptors used in phylogenetic trees in Figure 1 and in sequence identity matrices in Figures S1 and S2**

>NP_001516.2 orexin receptor type 1 [Homo sapiens]

MEPSATPGAQMGVPPGSREPSPVPPDYEDEFLRYLWRDYLYPKQYEWVLIAAYVAVFVVALVGNTLVCLAVWRNHHMRTVTNYFIVNLSLADVLVTAICLPASLLVDITESWLFGHALCKVIPYLQAVSVSVAVLTLSFIALDRWYAICHPLLFKSTARRARGSILGIWAVSLAIMVPQAAVMECSSVLPELANRTRLFSVCDERWADDLYPKIYHSCFFIVTYLAPLGLMAMAYFQIFRKLWGRQIPGTTSALVRNWKRPSDQLGDLEQGLSGEPQPRARAFLAEVKQMRARRKTAKMLMVVLLVFALCYLPISVLNVLKRVFGMFRQASDREAVYACFTFSHWLVYANSAANPIIYNFLSGKFREQFKAAFSCCLPGLGPCGSLKAPSPRSSASHKSLSLQSRCSISKISEHVVLTSVTTVLP

>NP_001517.2 orexin receptor type 2 [Homo sapiens]

MSGTKLEDSPPCRNWSSASELNETQEPFLNPTDYDDEEFLRYLWREYLHPKEYEWVLIAGYIIVFVVALIGNVLVCVAVWKNHHMRTVTNYFIVNLSLADVLVTITCLPATLVVDITETWFFGQSLCKVIPYLQTVSVSVSVLTLSCIALDRWYAICHPLMFKSTAKRARNSIVIIWIVSCIIMIPQAIVMECSTVFPGLANKTTLFTVCDERWGGEIYPKMYHICFFLVTYMAPLCLMVLAYLQIFRKLWCRQIPGTSSVVQRKWKPLQPVSQPRGPGQPTKSRMSAVAAEIKQIRARRKTARMLMIVLLVFAICYLPISILNVLKRVFGMFAHTEDRETVYAWFTFSHWLVYANSAANPIIYNFLSGKFREEFKAAFSCCCLGVHHRQEDRLTRGRTSTESRKSLTTQISNFDNISKLSEQVVLTSISTLPAANGAGPLQNW

>XP_017175645.1 orexin receptor type 1 isoform X1 [Mus musculus]

MEPSATPGAQPGVPTSSGEPFHLPPDYEDEFLRYLWRDYLYPKQYEWVLIAAYVAVFLIALVGNTLVCLAVWRNHHMRTVTNYFIVNLSLADVLVTAICLPASLLVDITESWLFGQALCKVIPYLQAVSVSVAVLTLSFIALDRWYAICHPLLFKSTARRARGSILGIWAVSLAVMVPQAAVMECSSVLPELANRTRLFSVCDEHWADELYPKIYHSCFFIVTYLAPLGLMAMAYFQIFRKLWGRQIPGTTSALVRNWKRPSEQLEAQHQGLCTEPQPRARAFLAEVKQMRARRKTAKMLMVVLLVFALCYLPISVLNVLKRVFGMFRQASDREAVYACFTFSHWLVYANSAANPIIYNFLSGKFREQFKAAFSCCLPGLGPGSSARHKSLSLQSRCSVSKVSEHVVLTTVTTVLS

>NP_001351480.1 orexin receptor type 2 isoform 2 [Mus musculus]

MSSTKLEDSLSRRNWSSASELNETQEPFLNPTDYDDEEFLRYLWREYLHPKEYEWVLIAGYIIVFVVALIGNVLVCVAVWKNHHMRTVTNYFIVNLSLADVLVTITCLPATLVVDITETWFFGQSLCKVIPYLQTVSVSVSVLTLSCIALDRWYAICHPLMFKSTAKRARNSIVVIWIVSCIIMIPQAIVMECSSMLPGLANKTTLFTVCDEHWGGEVYPKMYHICFFLVTYMAPLCLMILAYLQIFRKLWCRQIPGTSSVVQRKWKQQQPVSQPRGSGQQSKARISAVAAEIKQIRARRKTARMLMVVLLVFAICYLPISILNVLKRVFGMFTHTEDRETVYAWFTFSHWLVYANSAANPIIYNFLSGKFREEFKAAFSCCLGVHHRQGDRLARGRTSTESRKSLTTQISNFDNVSKLSEHVVLTSISTLPAANGAGPLQNW

>BAD72879.1 orexin receptor [Gallus gallus]

MSGTQPEDVSPPCRDWTSSPELNETREPFLNPSADYDDEEFLRYLWKEYLHPKGYEWALIAGYIVVFIVALVGNVLVCIAVWKNHHMRTVTNYFIVNLSLADILVTITCLPATLVVDITETWFFGHHLCKAIPYLQTVSVSVSVLTLSCIALDRWYAICHPLMFKSTAKRARNSIIIIWIVSCIIMIPQAIVMECSSVFPGLANKTTLFTVCDEHWGAEVYPKMYHTCFFLVTYMAPLCLMVLAYLQIFRKLWCRQIPGTSSVVQKKWKSLQSSAQQRGLGQSTKSKISAVAAEIKQIRARRKTARMLMVVLLVFALCYLPISILNILKRVFGMFNHADDRETVYAWFTFSHWLVYANSAANPIIYNFLSGKFREEFKAAFSCCIFGIHSHHDERLTRGRASTESRKSLTTQISNFDNVSKHSEHVLLTNINTLTANGITATFSPLKSMELHLHIPGVNVSSNLDEAVRISAGCTGNTENAEWDKFVPSVTKLTSMELQQG

>XP_003226120.1 PREDICTED: orexin receptor type 2 [Anolis carolinensis]

MSAAEVEDGLLAYRNCSLNMELNGTREPFGKPTADYDEEEFLRYLWREYLHPKEYEWVLIAGYIIVFLVALIGNILVCVAVWKNHHMRTVTNYFIVNLSLADVLVTITCLPATLVVDITETWFLGDSLCKGIPYLQTVSVSVSVLTLSCIALDRWYAICHPLMFKSTAKRARNSIIIIWIVSCIIMIPQAIVMECSSMFPELANKTILFTVCDEHWGAEIYPKLYHTCFFLITYMAPLCLMVLAYLQIFQKLWCRQIPGTSSVVQRKWKPLQPGVQTRGLRPSASLRISAVTAEIKQIRTRRKTARMLMVVLLVFALCYLPISILNILKRVFGMFNHASDRETVYAWFTFSHWLVYANSAANPIIYNFLSGKFREEFKAAFSFCCFDVRRHHHYHHDERVRGRISTESRKSLTTQISHFDHATKISEHVALSNINTLPPDGTAPIHLW

>XP_002934896.2 PREDICTED: orexin receptor type 2 [Xenopus tropicalis]

MLSPERSTGSRQMNSSMQGAKLDDLLYRNWSEQDVNGTQEPFLNPNADYDDEFLRYLWREYLHPKQYEWVLIVGYIIVFIIALIGNILVCVAVWKNHHMRTVTNYFIVNLSLADVLVTIICLPATLLVDITETWFFGKTLCKVIPYLQTVSVSVSVLTLSCIALDRWYAICHPLMFKSTAKRAQQSIVIIWIVSCAIMIPQAIVMECRSVFPELANKTILFTVCDERWEGQIYSKVYHICFFCITYMVPLCLMILAYLQIFRKLWCRQIPGTSSVVQKKWKPLQCSIQSKGQQSTKSRNNAVAAEIKQIHARRKTARMLMVVLLVFALCYLPISILNILKRVFGMFTHTNDRETVYAWFTFSHWLVYANSAANPIIYNFLSGKFREEFKAAFSCCCRGIHNNQDDRLIRGRASTESRKSLTTQISNCDNVSRLSEHVVLTNINTLNANGSGAVHNW

>NP_001073337.1 orexin receptor type 2 [Danio rerio]

MSGISVQRACNSCFTSAQHLNSSADTISHSHAENEDDELLKYIWREYLHPKQYEWVLIAGYILVFLVSLVGNTLVCFAVWKNHHMRTVTNYFIVNLSFADILVTITCLPASLVVDITETWFFGQTLCKILPYLQTISVSVSVLTLSCIAQDRWYAICHPLKFKSTAKRARKSIVLIWLVSCIMMIPQAVVMESSSLMPELTNKTSLFTVCDEQWPDEIYPKVYHTCFFIVTYFAPLCLMVLAYIQICHKLWCQQIPGSSSVLQRQWKSLQCSAHAVGSGESVKVRTSTVSAEAKQVKARRKTARMLMVVLFVFALCYLPISILNIMKRVFGAFKNTGNRETVYAWFTFSHWLIYANSAANPIIYNFLSGKFREEFKAAFICQCSGRGETHKQRARGRTSTDSRKSLSTQVNNLDNISRISDQAV

>XP_015203969.1 PREDICTED: orexin receptor type 1-like [Lepisosteus oculatus]

MDRAQLNASAPGASEPNGTAQAGGAHSDYEEEILRYLWKEYLFPRQYEWVLIAGYIFVFVVALTGNILVCLAVWRNHHMRTVTNYFIVNLSLADLLVTAICLPVSLVVDITESWFFGQTLCKVIPYLQTVSVSVSVLTLSFIALDRWYAICHPLMFKSTARRARNSIVLIWLLSLAIMVPQAVVMETSSMIPELANRTLLLSVCEERWGGEVYPRVYHVCFFLVTYLAPLCLMFMAYFQIFRKLWSRQIPGASGAVSRKWVRGAGSSDDGGQAPGVERPSGSAGLVARPGTAPSAEVKQLRARRKTAKMLLVVLLVFSLCYLPISVLNVLKRVSGVFDNAGDREAIYAWFTFSHWLVYANSAANPIIYNFLSGKFRGEFKAAFSCCFRGLRRCRGAEARRLARTTSQKSLTNGSKSEPLSSRVSEHVVLSSVRAVPS

>XP_006638920.1 PREDICTED: orexin receptor type 2 [Lepisosteus oculatus]

MSGVTANSVCEDCSPLLHEFNSSVESTHDPSVDGDDELLRYIWREYLHPKQYEWVLIAGYIIVFFISLIGNTLVCIAVWKNHHMRTVTNYFIVNLSFADVLVTITCLPASLVVDITETWFFGQTLCKVLPYVQTTSVSVSVLTLSCIALDRWYAICHPLMFKSTAKRARKSIVIIWIVSCVIMIPQAIVMECSSMVPELTNRTSLFTVCDEHWGDEIYPKVYHICFFIVTYLAPLCLMVLAYIQIFHKLWCQQIPGTSSVVQRKWRSLQRSAQSSTPGESARIRTNAAAAEIKQIRARRKTARMLMVVLFVFALCYLPISVLNVMKRVFGAFDNTSDREAVYAWFTFSHWLIYANSAANPIIYNFLSGKFREEFKAAFSCCCCEIRSPKEEHQIRGRTSTDSRKSLTTQLSNFDNVSRISEQLVLTSMGTLRSNDGDKTTW

>NP_071429.1 neuropeptide FF receptor [Homo sapiens]

MEGEPSQPPNSSWPLSQNGTNTEATPATNLTFSSYYQHTSPVAAMFIVAYALIFLLCMVGNTLVCFIVLKNRHMHTVTNMFILNLAVSDLLVGIFCMPTTLVDNLITGWPFDNATCKMSGLVQGMSVSASVFTLVAIAVERFRCIVHPFREKLTLRKALVTIAVIWALALLIMCPSAVTLTVTREEHHFMVDARNRSYPLYSCWEAWPEKGMRRVYTTVLFSHIYLAPLALIVVMYARIARKLCQAPGPAPGGEEAADPRASRRRARVVHMLVMVALFFTLSWLPLWALLLLIDYGQLSAPQLHLVTVYAFPFAHWLAFFNSSANPIIYGYFNENFRRGFQAAFRARLCPRPSGSHKEAYSERPGGLLHRRVFVVVRPSDSGLPSESGPSSGAPRPGRLPLRNGRVAHHGLPREGPGCSHLPLTIPAWDI

>AEP43759.1 pyroglutamylated RFamide peptide receptor [Homo sapiens]

MQALNITPEQFSRLLRDHNLTREQFIALYRLRPLVYTPELPGRAKLALVLTGVLIFALALFGNALVFYVVTRSKAMRTVTNIFICSLALSDLLITFFCIPVTMLQNISDNWLGGAFICKMVPFVQSTAVVTEILTMTCIAVERHQGLVHPFKMKWQYTNRRAFTMLGVVWLVAVIVGSPMWHVQQLEIKYDFLYEKEHICCLEEWTSPVHQKIYTTFILVILFLLPLMVMLILYSKIGYELWIKKRVGDGSVLRTIHGKEMSKIARKKKRAVIMMVTVVALFAVCWAPFHVVHMMIEYSNFEKEYDDVTIKMIFAIVQIIGFSNSICNPIVYAFMNENFKKNVSSAVCYCIVNKTFSPAQRHGNSGITMMRKKAKFSLRENPVEETKGEAFSDGNIEVKLCEQTEEKKKLKRHLALFRSELAENSPLDSGH

>NP_003848.1 galanin receptor type 2 [Homo sapiens]

MNVSGCPGAGNASQAGGGGGWHPEAVIVPLLFALIFLVGTVGNTLVLAVLLRGGQAVSTTNLFILNLGVADLCFILCCVPFQATIYTLDGWVFGSLLCKAVHFLIFLTMHASSFTLAAVSLDRYLAIRYPLHSRELRTPRNALAAIGLIWGLSLLFSGPYLSYYRQSQLANLTVCHPAWSAPRRRAMDICTFVFSYLLPVLVLGLTYARTLRYLWRAVDPVAAGSGARRAKRKVTRMILIVAALFCLCWMPHHALILCVWFGQFPLTRATYALRILSHLVSYANSCVNPIVYALVSKHFRKGFRTICAGLLGRAPGRASGRVCAAARGTHSGSVLERESSDLLHMSEAAGALRPCPGASQPCILEPCPGPSWQGPKAGDSILTVDVA

>AAP32295.1 endothelin receptor type B [Homo sapiens]

MQPPPSLCGRALVALVLACGLSRIWGEERGFPPDRATPLLQTAEIMTPPTKTLWPKGSNASLARSLAPAEVPKGDRTAGSPPRTISPPPCQGPIEIKETFKYINTVVSCLVFVLGIIGNSTLLRIIYKNKCMRNGPNILIASLALGDLLHIVIDIPINVYKLLAEDWPFGAEMCKLVPFIQKASVGITVLSLCALSIDRYRAVASWSRIKGIGVPKWTAVEIVLIWVVSVVLAVPEAIGFDIITMDYKGSYLRICLLHPVQKTAFMQFYKTAKDWWLFSFYFCLPLAITAFFYTLMTCEMLRKKSGMQIALNDHLKQRREVAKTVFCLVLVFALCWLPLHLSRILKLTLYNQNDPNRCELLSFLLVLDYIGINMASLNSCINPIALYLVSKRFKNCFKSCLCCWCQSFEEKQSLEEKQSCLKFKANDHGYDNFRSSNKYSSS

>AYA73846.1 orexin receptor-1 [Branchiostoma japonicum]

MSNNSTAAATEGVFNMTEDVYPTWLLPTPPDYDLEDYLLDYLYPKHYEWALIIAYILVFLLALIGNGLVCFVVIRNSHMRTVTNYFIANLSAGDLLVTIICLPPTLVVDIMETWFFGETMCKIIPYLQMVSVSVSVLTLCAIAVERWYAIVHPLKFKSTNARARTIICLIWVVSLSIMAPLIPMYKTNRTMPAEKTDLMMVCDEHWPDPIYGKIYHAAIVMVLFGVPIVLMMVSYCMIVWKLWSDQVPGISSSTSLRAPTRSNDSQRIKTPVSRSTSDNIVLSTSSSTAAVVTASSSFHSGTGSIADKTAENTVQSRRKVARMLVAVVVVFAICYIPLMILTFLKRVYGFFELMNDRSGLYAAFTVSHWLLYLNSAINPLIYNFMSEKFRSEFKASLPCCFPEAARKKREARGMTVGRPTLSRMHTTRTTGTTELLSRFESTATTRYPSTYEFTFARDRNEFGLSRFDSRPLPKSPILLHDTMKPRDLPKITYDAIQQDTDLRMISEHTSEPVAEPSEISCIVPTLCTNDIPNQIE

>XP_019619548.1 PREDICTED: orexin receptor type 1-like [Branchiostoma belcheri]

MSEDVYPTWLLLPTPPDYDFESYLLDYLYPKHYEWALIVGYILVFLLALIGNGLVCFVVARNSHMRTVTNYFIANLSAGDLLVTIICLPPTLVVDIMETWFFGETMCKIIPYLQMVSVSVSVLTLCAIAVERWYAIVHPLKFKSTNARARTIICLIWVVSLSIMAPLVPMYKTSRFYPEHKTDLMTVCDEHWPDPIYGKIYHAAIVMVLFGIPIVLMMVSYCMIVWKLWSDQVPGISSSTSLRAPSRSSDSQRIKTPVSRSTSDNIVLSTSSSAAATVVTASSSFHSGTGSIADKTAENTVQSRRKVARMLVAVVVVFAICYIPLMILTFLKRVYGFFDVTNNRQGVYAAFMVSHWLLYLNSAINPLIYNFMSEKFRSEFKASLPCCFPEAARKKREARGMTVGRPTISRMHTTRTTGTTELLSRFESTTRYPSTYEFAFARDRNEFGLSRFDTRPIPKSPILPHDTMKPRDLPKSTYETTQQDTDLRMISEHTSEPTAEPAEISCIVPALCTNDLPKQIE

>XP_019619556.1 PREDICTED: orexin receptor type 2-like [Branchiostoma belcheri]

MRTVTNYFIVNLSAGDLLVTIICLPPTLVVDIMETWFFGETMCKVFSYMQMVSVSVSVLTLCAVAVERWYAIVHPLKFKSTPARARNIIICIWVTSFLVTIPLLLVSKTTGTFPSDLTNLYVMCDEHWPENTFYGQIYHSAILGLIYIVPLCIISIAYTMIVWKLWSNQISLTVALKVTGNDVGKRRGSLVGQFRRSLRLKNSADFSTDFRPYTVSPADECPPSPRSHTVSPAEEQPRSASPSRDTIGQRSGETIFARRKVARMLIAVVIIFAVCWAPSMVMNFMKRVLGSFDVTNDRGGMYAAYGVAHWLIYFNSAINPVIYGFLSEKFRSGFQASLGCPQANKKKQNLIREVTVTRPTISRVMTTKTTSTADVGSRLESIRQYPFFTETVV

>XP_030838918.1 orexin receptor type 1 [Strongylocentrotus purpuratus]

MAGDYYGHTNLSQPHHLSSSVNGSTSSEWDYDDYIQGIYDQTRKRVYPEIHEYFLIAIYFIIFFVAIVGNSMVCIAILKNDHMRTVTNYYIMNLATTDIMIAVVCLPITITVDVSESWFFGQTACYLIPYFQLVLVCASIYTLMMIAVDRYLAICHPLKFQIRASRTLLTIALVWVVSFFIALPVAVVNGLESQPASVHIGKPLWRMSCTESRWVSKVWEKLYHTAFFLAVYIVPLAVIGVAYTRVCRRLWSGIPTEEGHGASKPSFNQANVVSTTTISKSTEAQLKSRRKVASMLIVVVVTFAICFFPFQLLNVLKKHNAFGNLRDASSSAQYNAVYIPYIIGHLMAFINSAINPIIYNFMSAKFRQAFKSMFDCLPCCRSPRQSPAIDGTSAGPAYRRANSTGVSDTHTTEYVPMTSIRNGRGVNNNISAMSKGV

>XP_002733613.1 PREDICTED: orexin receptor type 2-like [Saccoglossus kowalevskii]

MAYDCIWSGNLSYYNSTEGDISLFPCGDNDTSSENAYDWDYYYDVKLPTRLRNFVYPAPHEWFLISVYALVFLMALVGNVLVCFAVLRNQQMRTVTNYYIVNLSFADILVSLICLPVTVTFETTETWYFGDLACKIIPYVQVVSMSVSVLTLSAIAVDRYFAICQPLLFKSTAKRTLTIIFSIWLVSFIIPIPQAIVYETEAADTYKRTYIYFTKCYEKVWFGTIQQKIYHVALVLVIYVIPLLLIGIAYLFICRQLWATIPGTMPSGGGKHCHSNGSDMNRTTMSQLKSRRKVANMLIIVAILFALCYLPLHLLNIIRQFPIFDEVVQEDRHSFHIPFLVAHWLAFANSATNPVVYNFLSAKFRKEFKAAFTCCLSCCARKRRRRHKRGGYQGSIMHSSMASTSKSYGNCTEHISMSTVRTGVHV

>ADX66344.1 allatotropin receptor [Manduca sexta]

MLNKSINISILLLIIVESSTSEIIEDNITREPIKATELNRRIIRLIEIKESNEDLPNRYKRSLPENDKEPPETKENTTEECVGAAEFCNMTKEAYIAMLQEYIYPQTYEWVLIATHSIVFLTGLIGNALVCIAVYRNHSMRTVTNYFIVNLAVADFMVILFCLPATVLWDVTETWFLGDVLCKMLLYFQSVSVTVSVLTLTFISVDRWYAICFPLKFKSTTSRAKTAILIIWILSLSFNSPDLVVLKTDKPVPLRFELEYLVQCIATWSSQADPVWHILKVVFIYTIPLLLMTVAYLQIVRVLWHSDKIPGQAETIKLAPAEQTQLRSRRKAAKMLVAVVVMFAVCYFPVHLLSVLRYTLDMEQSDAITFLALVSHVMCYANSAVNPLIYNFMSGKFRREFRRAFCCSSRPVHENFTSLTRVTTSKKKEQSCDKSLSQRNVSNTTFIQNGFKSGYYA

>AEX08666.2 allatotropin receptor [Schistocerca gregaria]

MTENETDYYSQWESALNESNASEATTSSPLYLAWWTLSPSSNVTATTLVVNASTPDFSLDEDGNATEGQNCTNDYCIPDIDYWNMVYQHVYPKDYEWILIAMHSLVFVAGLVGNALVCLAVYRNHAMRTVTNYFIVNLAVADFMVILFCLPPTVLWDVTETWFMGTGLCKVVLYLQTVSVAVSVLTLTFISVDRWYAICFPLRFNSTTGRAKTAIAIIWLLALAFDIPELVVLRARGRDWDSVLLTQCEGSWSYDSEMVFHGAKSLLLYTLPLLFMSVAYFQIVRVLWRSDNIPGHDDHNGDVISSKEAGHHATFAPSGSVGSRRVPMAGNSTTEAQLRSRRKAAKMLVAVVAMFAICYLPVHLLNILRYTVDIPQNDTTSAISMLSHWLCYANSAVNPVIYNFMSGKFRAEFRRLFWTCAYGSNRYSPAPGAAPSAAMVARHGGARQRPGGGGAASSAGHEMRSLYRRGPGP

>AEN03789.1 allatotropin receptor [Aedes aegypti]

MSVRIDRSLEPSSEGKPPAMTTSNFNGAICRDGNNVGTEAEQGSGSCALVNNGTKSPLAAGLDGNQTVVTPYYTIVNLDNHNDVLCDEEYDTEEYNENCFIDHNVTCVGDPLYCNLTYDEYRQLLMDYIYPSTAEWILIASHSVVFIMGLVGNALVCIAVYTNHSMRTVTNIFIVNLAVADFFVILFCLPPTVVWDVTETWFMGKAMCKVVIYFQTVSVTVSVLTLTYISIDRWYAICFPLRYKPRPERAWRFIAVIWLIGFLSDLPEFLVLTTRRKKLRFDIKLFTQCVSTWDNEKEKTFYIVKFVFLYSLPLLFMTIAYFQIVRVLWRSDTIPGHRESRTQPYGIHSTRTTLNCVGNTSTMGQLRARRKAAKMLVAVVVMFASCYFPVHMLNVARYTFDIGQSDVVAVLSLFSHWLCYANSAVNPVIYNFMSGKFRREFKNALEKCHCLRNPRGLGGRVGGYDDRSMYHTATRMNASPSSRSNYHLTSVRNISIKHTQQTSFNNGSRHHHARNSINHPGSLTGAPQISPVSFEERMALTKNMDGNIGCGDPTMAGTATSVASRAEGNSSGHVGANSNTNHHHLHHHGVACNGSTPDAPATTTGTAPPTNGGSSSMLMIVNKSSNCKINGT

>KAE8742626.1 Allatotropin Receptor [Frankliniella occidentalis]

MAVTSPPLLDNDSAPDGWNGTYFNDSCRNYTNDYCVPDEEYLDMMLEYIMPTKMEWVIIAMHCAVFIGGLVGNALVCLAVYRNHTMRTVTNYFIVNLAVADFLVILMCLPPTVLWDVTETWFMGTALCKIVLYFQTVSVTVSVLTLTTISVDRWYAICFPLKFKSTTSRAKKAIIIIWLLALSFDVPELVVLETKRKALGIDTIFFTQCLPTWGDSSETTYHCVKTLFLFFLPLAFMTVTYVQIVKVLWSKTNIPGHAETKSLSYQYCNGNGVSGTRRTMHRSISASSQILSRRKAAKMLVVVVLMFFICYLPVHLLSILRYTMIIPQTELMTVTAMFVHWLCYANSAVNPLIYNFMSGKFRGEFRLAFQQCACESWVLGGDTVRPAAGGGGPGGGPGPGALAGVSPGHVVLGMSQLHRRPAPRAAPRAASTLAATATTTLSVSVLDSGP

>AIT70966.1 allatotropin receptor [Helicoverpa armigera armigera]

MNFDKKISIFIIGLIILTSVEGIHAEETRTGIKNNETKSRHNKTIEDLTAENATASDEPSKENATEVCVGQKEFCNLSKEEYVSMLNNYIYPHTYEWVLIGTHTLVFITGLVGNALVCVAVYRNHSMRTVTNYFIVNLAAADFMVILFCLPATVVWDVTETWFLGDVLCKMLLYFQSVSVTVSVLTLTFISVDRWYAICFPLKFKSTTGRAKTAILIIWTLSLIFNAPELVVLTTEKSVPLRFELEYLVQCVATWSSNSDLVWHIIKVIFIYTLPLLLMTVAYYQIVKVLWRSEKIPGHAETMKLAPAEQTQLRSRRKAAKMLVAVVIMFAVCYFPVHLLSVLRYTLDMEQNDVITCLALISHVMIYANSAINPLIYNFMSGKFRREFRRAFCCSTASDLENFTTLSRITTSKKRPCALMTFETKTQGRNVCSTTFVHSSYKDRLT

>OWR50755.1 allatotropin receptor [Danaus plexippus plexippus]

MAIKIILALAVLIIYFHKNDAKIRFNGLQEDFMIESHNNDIFGEDTFLRLKRSVEQDKKLLIGDNNKSKNEIESNSSEPCVGDAEFCNMTREDYIQMLYEYIYPQTYEWVLIGVHTTVFVIGLIGNLLVCLAVYRNHAMRTVTNYFLVNLAVADFMVLLFCLPATVLWDVTETWFLGDALCKILLYIQSVSVTVSVLTLTFISVDRWYAICFPLKFKSTINSAKTAILVIWALSLVFNTPELVVLTTVKVVPLRFDLEYLVQCTATWSYSSDLIWHIIRIVFVYTVPLLLMTVAYHQIVRVLWSSQKIPGLAETMKLASAEQIQLQSRRKAAKMLVAVVVMFAVCYFPVHLLSVLRYLDMEQNDMITCLALVSHVLCYVNSAINPLIYNFMSGKYRREFRRVFCCNQNLTRNTFTTMTRLTTSRKKYETADKTQRSSLKFHKCENMALRHHNCGLALKSQCGHIALNERVNELNQGFRVCENVMNNGQRCSIKAIGF

>NP_001291369.1 allatotropin receptor precursor [Bombus terrestris]

MHPLELVIVGWLASVISTLVDAIDYLDDYSAMDYTDESDIDYNATNCTNSYCISNEEYVDRMINYIFPKFWDWVLIASHSVVFVVGLVGNALVCIAVYRNHSMRTVTNYFIVNLAVADFLVLLLCLPFTVLWDITETWFLGLTLCKAVPYLQTVSVTVSILTLTFISIDRWYAICFPLRFKSTTGRAKSAIIGIWAAALLFDIPDLVVLHTVPPTHIKIKTVLFTQCDISWSQRSQVAFTIVKLIFLYTGPLIFMSVAYWQIVKVLWRSNIPGHNLPSRASQMSQIPSTGGGNPEVQLRSRRKAAKMLVTVVITFAICYFPVHLLSVLRYTTTLPSNKWINAISLIAHGLCYFNSAVNPLIYNFMSGKFRKAFRRTFRCARENGSRIQRGYLASTSNFPRIKSRTTTIRTTFKNNNNLQRNTEIIPLSAITTIQQNEKHD

>AKQ63030.1 allatotropin receptor 2 [Platynereis dumerilii]

MAVRNYSVDNPSVLEIDAMHPAYEDGDFGITETPPNATNCRNEYCVSDEEYLDMIKAYVFPSRFEWVLIVLYIQVFTIGLCGNLLVCFAVWRNQHMRTVTNYFIVNLAVADLLVIIICLPPTVLVDVSETWYMGAVMCKVVHYMQGVSVSVSVLTLSCISVERWYAICHPLTFRSTTTRVRSIIVVTWVVALVILIPELIVLDTSSKYENLTILLTVCRPTMLPFYNPMAYELFKMVALYFLPIILMSVTYGNIVICLWSNAIPCEPTTASSRPLHNNSRTTAEAQLIARRKAAKMLIAVVVMFGVCYLPVHLTNILRYAKLLPESENITFFPLVAHWLCYFNSAINPVIYNFMSARFRNEFKHACSCCTRVWCRDVRLRRRQGDSMYSYRYTNDMSQTEQMTLTTIRPDINHVDE

>AKQ63076.1 allatotropin receptor 1 [Platynereis dumerilii]

MAVRNYSVDNPSVLEIDAMHPAYEDGDFGITETPPNATNCRNEYCVSDEEYLDMIKAYVFPSRFEWVLIVLYIQVFTIGLCGNLLVCFAVWRNQHMRTVTNYFIVNLAVADLLVIIICLPPTVLVDVSETWYMGAVMCKVVHYMQGVSVSVSVLTLSCISVERWYAICHPLTFRSTTTRVRSIIVVTWVVALVILIPELIVLDTSSKYENLTILLTVCRPTMLPFYNPMAYELFKMVALYFLPIILMSVTYGNIVICLWSNAIPCEPTTASSRPLHNNSRTTAEAQLIARRKAAKMLIAVVVMFGVCYLPVHLTNILRYAKLLPESENITFFPLVAHWLCYFNSAINPVIYNFMSEKFQKAFRNTLFCCSSPCMEGKCCNVCNSKSDKPAQPACV

>XP_002127187.1 orexin receptor type 1 isoform X2 [Ciona intestinalis]

MNSRALTSTVLTAGRKFSEANYSVGINSTAVSINQLDFDIWEYYLKPTNAEWFVMSLYVLVFLISIIGNCLTIAFILRRKHLRTTINYFMLNLALADIMVTIICLPPTLMVDFMESWLVGQFLCKFTPYLQMAVTSVSSLSLGAIAVNRWFVVCHPLKVARTRRSAKHALLTMTSIWLFSLITLCPIIFVTELTEDFPGYKELNLLKSCGEHWTTFLHQAVFHIYYVTVCYALPLMVMAIAYTNVFRKLSYTKIPGHVSRETNPIPKRRGQCHSCSSNSEHTHRGSTIGSEPNSPSKSNPSSPTAKKQDGASVGQEHVRNGIDLPMPREQESLFYTGAWKVKRDSEDYKKLYLQRNRNSSTFSKLITSRKTQRTYCRKCKIKRNLIQSRKRSGRIQVALVVVYFLCYSPAMVLDLIRRTSDLFTSVHRESTYFLFAIAHLLVYLNSALNPIIYNCFSVRVSGEILFVQRPRFARSKTPRTVQAVI

>XP_018673071.1 Cint orexin receptor type 2 isoform X1 [Ciona intestinalis]

MNSRALTSTVLTAGRKFSEANYSVGINSTAVSINQLDFDIWEYYLKPTNAEWFVMSLYVLVFLISIIGNCLTIAFILRRKHLRTTINYFMLNLALADIMVTIICLPPTLMVDFMESWLVGQFLCKFTPYLQMAVTSVSSLSLGAIAVNRWFVVCHPLKVARTRRSAKHALLTMTSIWLFSLITLCPIIFVTELTEDFPGYKELNLLKSCGEHWTTFLHQAVFHIYYVTVCYALPLMVMAIAYTNVFRKLSYTKIPGHVSRETNPIPKRRGQCHSCSSNSEHTHRGSTIGSEPNSPSKSNPSSPTAKKQDGASVGQEHVRNGIDLPMPREQESLFYTGAWKVKRDSEDYKKLYLQRNRNSSTFSKLITSRKTQRTYCRKCKIKRNLIQSRKRSGRIQVALVVVYFLCYSPAMVLDLIRRTSDLFTSVHRESTYFLFAIAHLLVYLNSALNPIIYNCFSVQFRKEFRLTFNCCFSSSSQRRNSVRSTAALRSLEDTKDRTSCDMML

>ENSCSAVP00000016473_Csav/1-346 [Ciona savignyi]

IWEFYLKPTQTEWFVISLYAIVFLTSIVGNCLTIAFILRRKHLRTTINYFMLNLALADIMVTIICLPPTLMVDFMESWLVGQFLCKFTPYLQMAVTSVSSLSLGAIALNRWFVVCHPLRVARSRRSAKNSLISMACIWLFSLITLCPVVFVTELTDDFPGYSELELLKSCGEHWATFLHGAVFHVYFVTVCFALPLVVMAIAYTSVFRKLSRTKVTKRTVSQLFKRLGESIPTFKRGLRAGFADKVESQQSDSEKPNLISSRKRSGRIQVALVIVYFLCYSPAMVLDVIRRTSNVFDSVHRESTYFMFAIAHLLMYLNSALNPIIYNCFSVQFRKEFRLTFSCCFP

>XP_011445021.1 PREDICTED: orexin receptor type 2 [Crassostrea gigas]

MINMKESQNHTREKTDVGTEDAIALFIVKEHFEKLMMENLTNIPANLSGTRDNSSVPIECTDIFCRPDEEYLDYLEDYVFPDDWEWGIIILYALTFIVGLSGNVLVCFAVWRNRSMRTVTNIFIVNLAIADLAVIIICLPPTLLSDVTETWYFGFAMCKIALFLQTTSVAVSVFTLSAISVERWYAICYPLRFKSTKRRAKIIILVIWIIAFLLALPEVIVADLTRFVKRQYIDLLIFCGPQWSDKTNQVVYQSVIIVLMYLLPLVLMTVTYSMIAVVLWTGKIPGAIESANRPMMDGNVNRAEEQLESRKKAAKMLITVVIGFAVCYFPVHLFNILRYADALRFVAPRMIQVLSMISHWLPYLNSSINPIIYNFMSAKFRKEFTAACCCTKRRRAFSVHYKSGVSTFSCASQYTHRNNSNSCTEQVLLSTYPDH

>XP_014772633.1 PREDICTED: orexin receptor type 2-like [Octopus bimaculoides]

MDNSTGDVLSDESSTAPCYNVYCLSDEDYINMVEQHVQPNAGEWILVVIFIILFIVGLVGNFLVCYAVIKNSQMRTVTNLFIMNLAIADFMVILICLPSSLLVDVSETWFFGEVMCKIFLYLQTVSVAVSVLTLSAISIERWYAICHPLSFKSTASRARNIILTIWLLSACVASPDLVTARTYRSLPMRYNYVKWLVSCRPSWTQRSQFIYQMFLFIALYFLPFCLMAFTYTRITLVLWREDIPGVNETAGGHRLMAENRNPNTNAQLQTRRKAAKMLITVVIVFGICNLPVHILNIVRYANISNNLKAISIFSLISRLLCYVNSAINPIIYNFMSAKFRKEFKSVCLCCVSPLEQEQHTQRPKSGGSYNISYSRTNCQTEQFTLISVKE

**Sequences of propeptides used in the orexin propeptide figure Figure 3**

>NP_001515.1 orexin precursor [Homo sapiens]

MNLPSTKVSWAAVTLLLLLLLLPPALLSSGAAAQPLPDCCRQKTCSCRLYELLHGAGNHAAGILTLGKRRSGPPGLQGRLQRLLQASGNHAAGILTMGRRAGAEPAPRPCLGRRCSAPAAASVAPGGQSGI

>NP_034540.1 orexin precursor [Mus musculus]

MNFPSTKVPWAAVTLLLLLLLPPALLSLGVDAQPLPDCCRQKTCSCRLYELLHGAGNHAAGILTLGKRRPGPPGLQGRLQRLLQANGNHAAGILTMGRRAGAELEPHPCSGRGCPTVTTTALAPRGGSGV

>NP_989516.1 orexin precursor [Gallus gallus]

MEVPNAKLQRSACLLLLLLLLCSLAGGRQSLPECCRQKTCSCRIYDLLHGMGNHAAGILTLGKRKSIPPAFQSRLYRLLHGSGNHAAGILTIGKREERPGTACRDALSCAAGTQPTVTPRGTAASPRECQEHAEKDLTKG

WAAAKSFY

>NP_001070860.1 orexin precursor [Danio rerio]

MDCTAKKLQVLVFMALLAHLARDAEGVASCCARAPGSCKLYEMLCRAGRRNDSSVARHLVHLNNDAAVGILTLGKRKVGESRVHDRLQQLLHNSRNQAAGILTMGKRLEEPAKFLIPTVPQDVDSYEKR

>AMN82803.1 orexin precursor [Oncorhynchus mykiss]

MGFNTKHLTTGPGMDTACSTTKKLKVLILLLLVSHLACDAQGVANCCKQKSHSCRLYVLLCRSGNGMGTRGPLTDDAATGILTLGKRKETDERRFQNRLNQLLHGSRNQAAGILTMGKRTEDTAEPLMCLFPHLETAPTTTTQLVLQLPFK

>XP_007884949.1 PREDICTED: orexin, partial [Callorhinchus milii]

RSALALALLLLCSLVSVSSPVPDCCRRKTCPCHLLTRLRGTGNHAAGILTLGKRRAGSVLDFRSQLYRLLRGSGNHAAGILTMGKREGRGVG

>XM_026835150.1 PREDICTED: Ciona intestinalis uncharacterized LOC113474376 (LOC113474376), mRNA, translated: 5'3' Frame 2

MNLQCILLVCLLGYCTATIPNECCDNPDLQNYSKCLGTERICELMSLLNENRETGGIMATGKRSRDICDDFRIISPRLKAMCEANANKLKSTMDKKYEASVGNWFWN

>reftig:CSAV2.0:reftig_2:2296967:2297917:-1 [Ciona savignyi], translated: 5'3' Frame 1

MKMLIFLLVLTVFVYYVSGVPTECCSSYPGSARSAECEGTERICELMALLNEDGGTGGILPTGKRSGLVDPYQQCWLAKRLSPKLAAHCMSLMEKYKRSDVARDRGYHPDVGNWLWY

>XP_019618039.1 PREDICTED: uncharacterized protein LOC109465289 [Branchiostoma belcheri]

MRCMHVLLAVLFVGLLCKGSWAFPRCCWSRKCNCPYFRRLFGPRNHGHGILTVGKRQRGEAEYKSPSSRGEHDPTHGGLPATLVDLHPPQVDLHRPHGDGLPERTLDHGYSERMNHLEHSKVWDLLHHLRRLLNLTAEADARSRRRLTDVQPYETSPRHWQISMRR

>XP_002598524.1 hypothetical protein BRAFLDRAFT_66903 [Branchiostoma floridae]

MRCMHVLLAVLFVGLLCKGSWAFPRCCWRRTCNCPHFRRLFGPRNHGHGILTVGKRQRGEGEYKSLPRGEHDPSHGGLPTSLVDLHDRAQVDLDRLEGLPELTLDHDYRQMRCMHVLLAVLFVGLLCKGSWAFPRCCWRRTCNCPHFRRLFGPRNHGHGILTVGKRQRGEGEYKSLPRGEHDPSHGGLPTSLVDLHDRAQVDLDRLEGLPELTLDHDYSERKNQLEHSKVWELLHHLRRLLNLTAEADARSRRRQTELQPYETTPRHWQISMRR

>ASK86256.1 orexin 2 precursor [Ophionotus victoriae]

MPQTGPFLSLIVFTILLYLTGLLAQKQSCCRVKGCSIPPDCDCPLKQELCKDVTKGILSMGKRTRSYEENVYRQLDQNKDRQHQGTRTSSKVMNTILKLLQSQEQEDEPQDWKPMLSRNLWENEDFNEDLYDQQPNFYAD

>ALJ99960.1 orexin-type precursor 2 [Asterias rubens]

MRTTTLFVIQTVVVIGYFTCSSTAAANACCRGTCHDIPPGCNCPYKSYLCGELNALTMGKRKADDTSYLLTQEQETQQQNQQRRTQQTQPWVDRQPDDDRIVDVLNNLLKLFKETHQGDQDGFDLQDQSDDWEPVTSSRKQQSENAHNVYRHQPLFSADIL

>BCM_Contig134164_Spur

MNRGVPSQIPRVLLAISLVLLVCLPEISHADRACCKRTVGCNLRSDCTCRIREITCTDPSLGLQNYGKRSPARSPTYYDSYTPILERIAELASLQQDDRWKRKRSSSSSSRYPFYDYNMRPF

>XM_002734948.2 PREDICTED: Saccoglossus kowalevskii uncharacterized LOC100370804 (LOC100370804), mRNA, translated: 5'3' Frame 1

MRTTIMLIFSAVLVAVLFTHTSAQPQCCRGVGCKIPPNCKCPFQSIICDNPTKNVLTAGKRNYLPISKSNELSTYQRPVSDAKTNVVTSDDIVNLIRSSPSLIRKIVKAIDLNGDDMISKAELQSLVYD

>CBBN5296.fwd CBBN Lottia gigantea 3,4,5,6.5d Larvae (M) Lottia gigantea cDNA clone CBBN5296 5', mRNA sequence, translated: 5'3' Frame 3

MKLTLVLVTVTLTITILANAYPQSPSLSHHRSKRGFKANSASRVAHGYGKRGFPSWKNYFQDGGSDVLSVEDMAELVAENPSLAKALLRKFVDTDNDGIISTTELLGTKQMLK

>AAB08759.1 allatotropin [Manduca sexta]

MNLTMQLAVIVAVCLCLAEGAPDVRLTRTKQQRPTRGFKNVEMMTARGFGKRDRPHPRAERDVDHQAPSARPNRGTPTFKSPTVGIARDFGKRASQYGNEEEIRVTRGTFKPNSNILIARGYGKRTQLPQIDGVYGLDNFWEMLETSPEREVQEVDEKTLESIPLDWFVNEMLNNPDFARSVVRKFIDLNQDGMLSSEELLRNF

**Table S1**. Taxonomy and species list of used orexin/allatotropin receptor and PPO and prepro-allatotropin sequences.

| **Superphylum** | **Phylum** | **Subphylum** | **Species** | **in English** | **Code** |
| --- | --- | --- | --- | --- | --- |
| Deuterostomia | Chordata | Vertebrata | *Homo sapiens* | human | H. sap |
|  |  |  | *Mus musculus* | house mouse | M. mus |
|  |  |  | *Gallus* | red junglefowl, chicken | G. gal |
|  |  |  | *Xenopus tropicalis* | western clawed frog | X. tro |
|  |  |  | *Latimeria chalumnae* | coelacanth | L. cha |
|  |  |  | *Danio rerio* | zebrafish | D. rer |
|  |  |  | *Lepisosteus oculatus* | spotted gar | L. ocu |
|  |  |  | *Oncorhynchus mykiss* | rainbow trout | O. myk |
|  |  |  | *Callorhinchus milii* | elephant shark | C. mil |
|  |  | Tunicata | *Ciona intestinalis* | ascidian | C. int |
|  |  |  | *Ciona savignyi* | ascidian | C. sav |
|  |  | Cephalochordata | *Branchiostoma belcheri* | lancelet | B. bel |
|  |  |  | *Branchiostoma floridae* | lancelet | B. flo |
|  |  |  | *Branchiostoma japonicum* | lancelet | B. jap |
|  | Ambulacraria | Echinodermata | *Ophionotus victoriae* | brittle star | O. vic |
|  |  |  | *Asterias rubens* | common starfish | A. rub |
|  |  |  | *Strongylocentrotus purpuratus* | purple sea urchin | S. pur |
|  |  | Hemichordata | *Saccoglossus kowalevskii* | acorn worm | S. kow |
| Protostomia | Spiralia | Mollusca | *Lottia gigantea* | owl limpet | L. gig |
|  |  |  | *Crassostrea gigas* | Pacific oyster | C. gig |
|  |  |  | *Octopus bimaculoides* | California two-spot octopus | O. bim |
|  |  | Annelida | *Platynereis dumerilii* | ragworm | P. dum |
|  | Ecdysozoa | Arthopoda | *Manduca sexta* | tobacco hawk moth | M. sex |
|  |  |  | *Helicoverpa armigera* | cotton bollworm | H. arm |
|  |  |  | *Danaus plexippus* | monarch butterfly | D. ple |
|  |  |  | *Bombus terrestris* | buff-tailed bumblebee | B. ter |
|  |  |  | *Frankliniella occidentalis* | western flower thrips | F. occ |
|  |  |  | *Schistocerca gregaria* | desert locust | S. gre |
|  |  |  | *Aedes aegypti* | yellow fever mosquito | A. aeg |

**Figure S1**. Amino acid identity between the receptors' predicted TM sequences (sequences as given above).

**Figure S2**. Amino acid identity between the receptors' full sequences (sequences as given above).

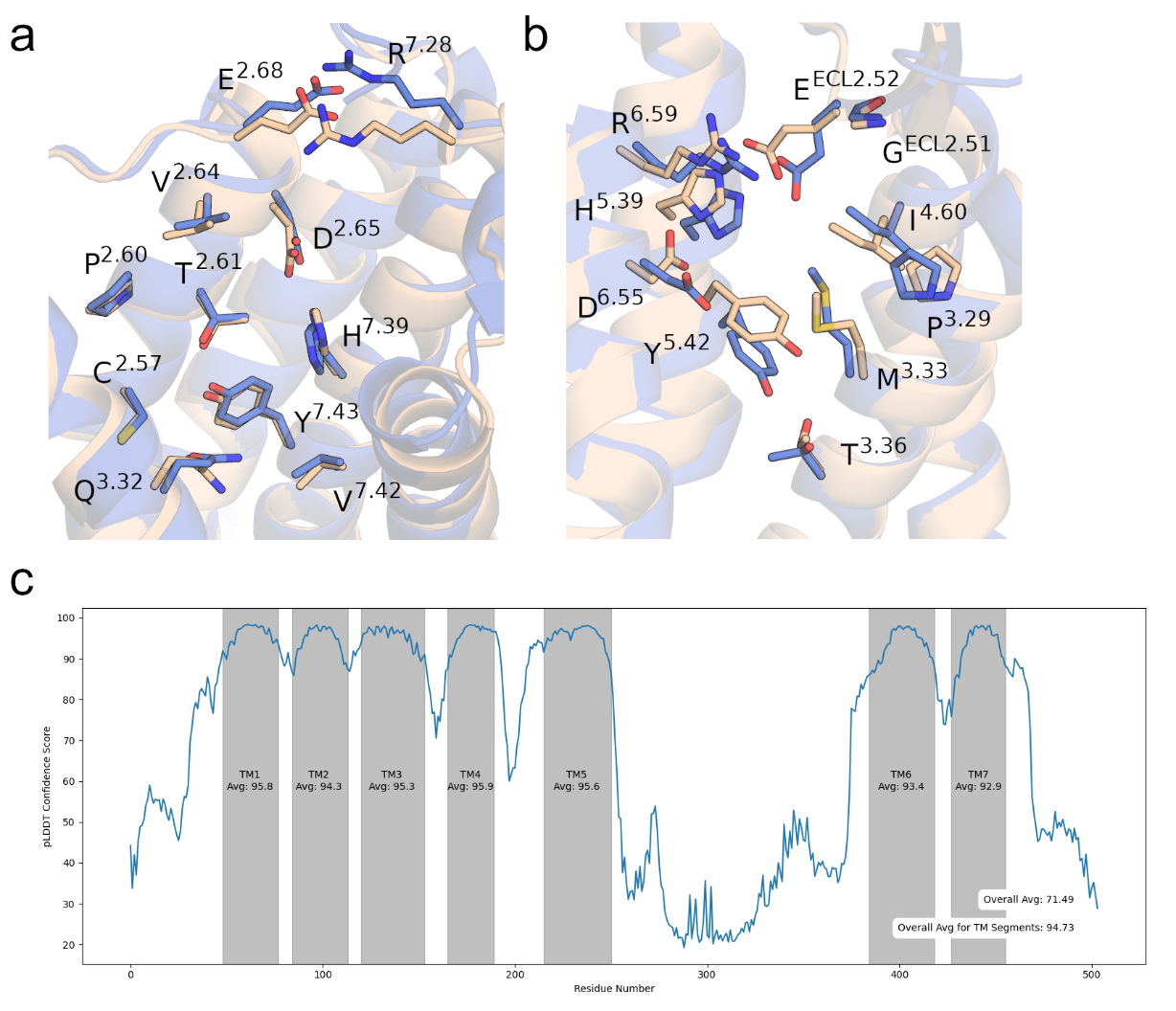


**Figure S3**. Superimposition of a model of CiOX constructed using AlphaFold (blue cartoon and stick) and the homology model of CiOX presented in the manuscript (light brown cartoon and sticks). The overall Cα superimposition RMSD is 5.0 Å, and the Cα RMSD for the superimposition of only the TM regions is 0.98 Å. a) Amino acids of the transmembrane cavity shown in Figure 2b from TMs 2, 3 and 7. b) Amino acids of the transmembrane cavity shown in Figure 2b from TMs 3-6. c) Reported pLDDT confidence score of the CiOX AlphaFold model, showing high confidence for the TM regions. The high confidence is likely a consequence of the high number of GPCRs in the PDB, including orexin receptors.


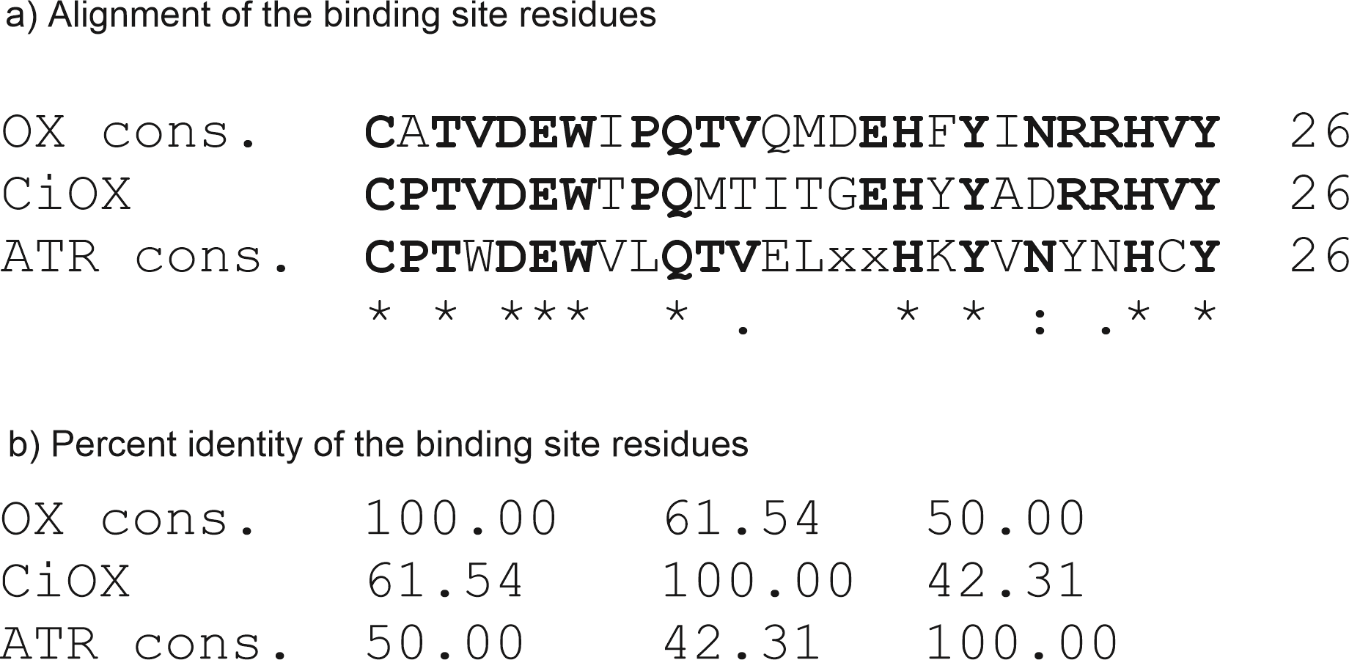


**Figure S4.** Conservation of the binding site. a) Alignment of consensus sequences of the binding site residues of vertebrate orexin receptors and ATRs (sequences as given above) and the binding site residues of CiOX. Conserved (between any group) residues bolded; x: no conservation in consensus. Consensus sequence (cons.) constructed with EMBOSS Cons with default parameters. b) %identity of the binding site residues.


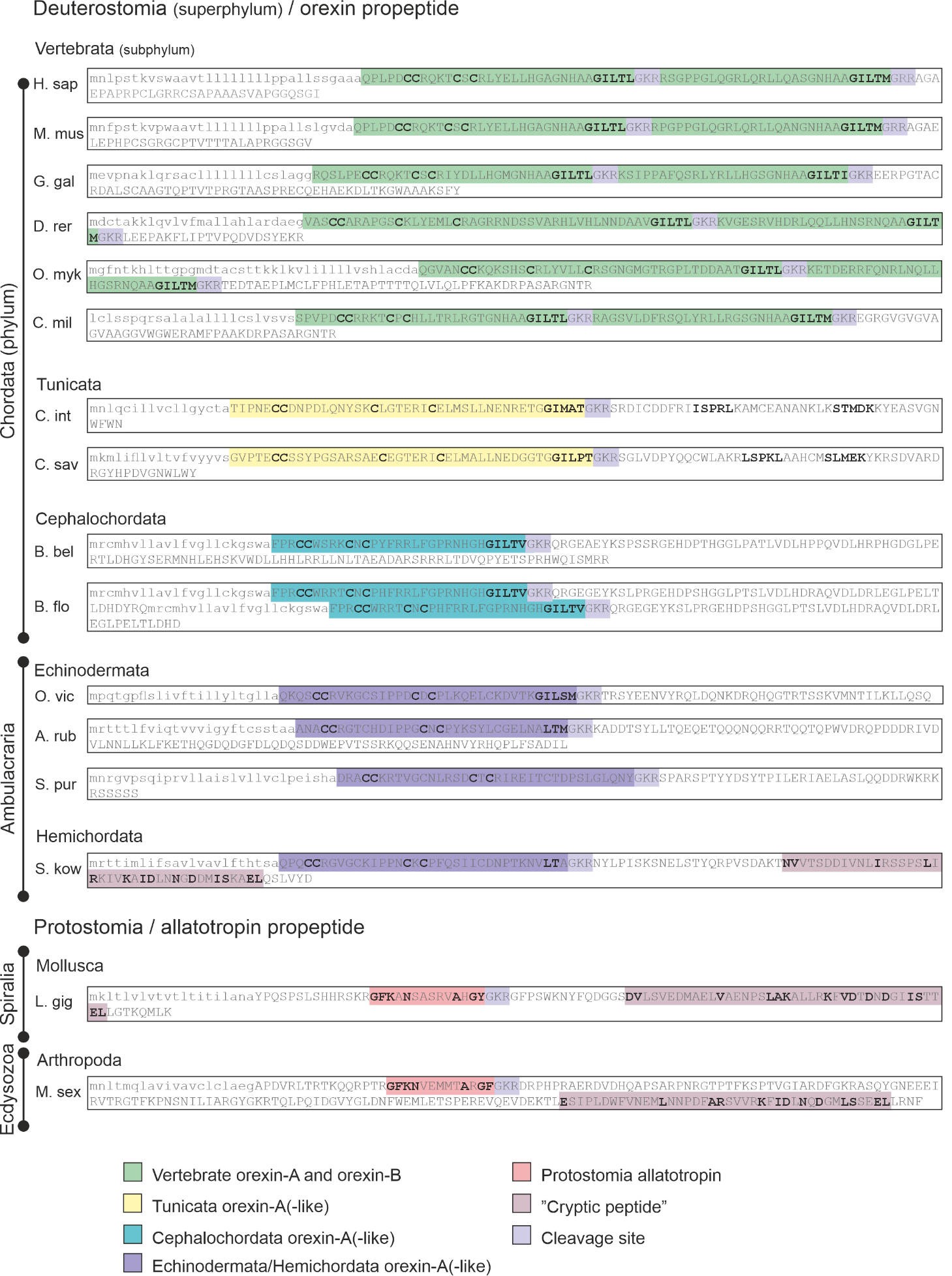


**Figure S5**. The orexin/allatotropin propeptides through different species groups. Signal peptide (predicted by SignalP-5.0) in lower case.


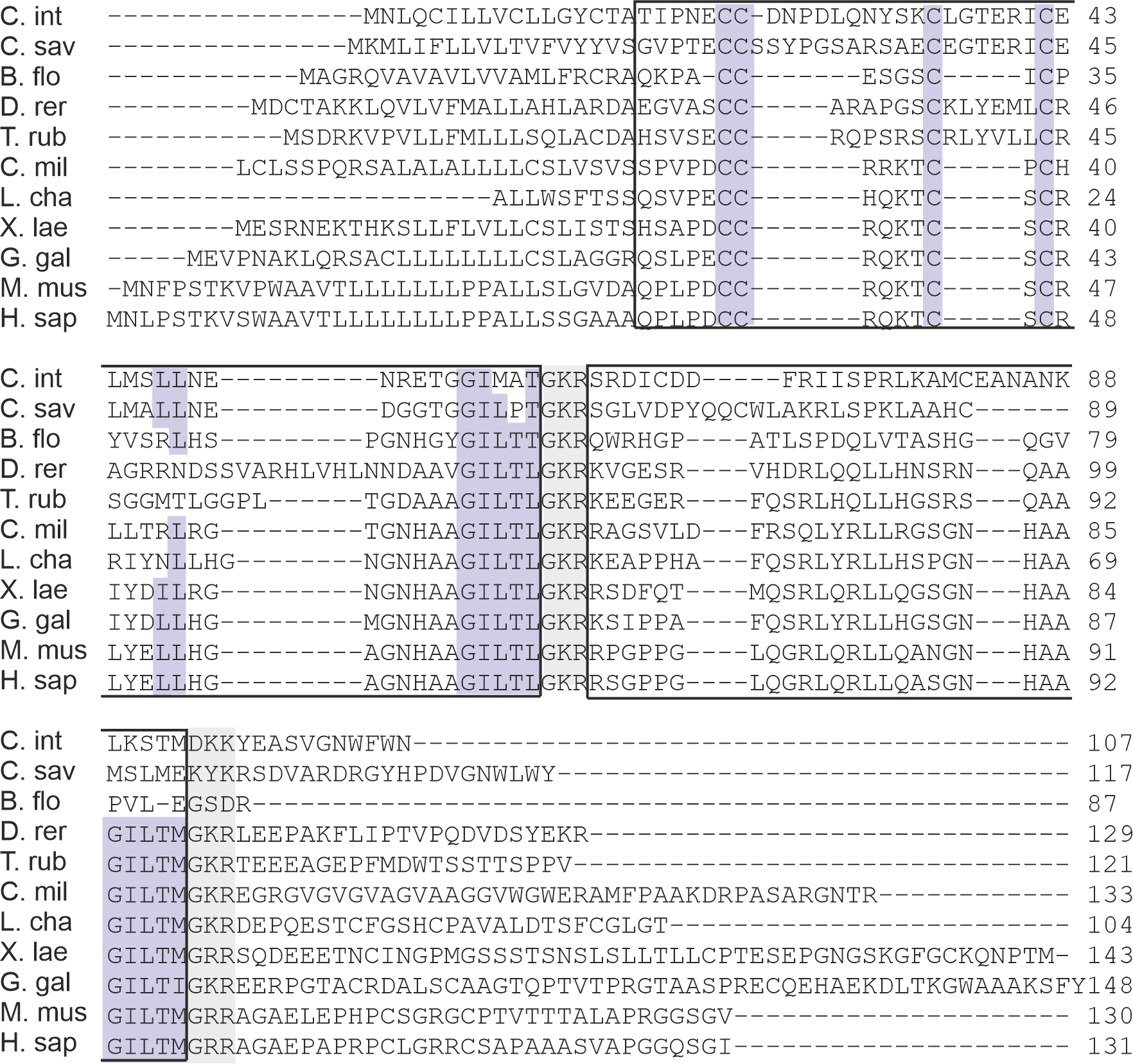


**Figure S6**. Alignment of PPOs of invertebrates *C. intestinalis, C. savignyi* and *B. floridae* (lancelet) and vertebrates *D. rerio* (zebrafish), *Takifugu rubripes* (pufferfish)*, Callorhinchus milii* (ghostshark), *Latimeria chalumnae* (coelacanth)*, Xenopus laevis* (frog)*, G. gallus* (chicken)*, H. sapiens* (human) *and M. musculus* (mouse). Conserved regions are highlighted with light violet and cleavage sites with grey, while the black boxes represent the orexin-A and orexin-B (or equivalent region). The alignment has been manually modified to prevent gaps within secondary structure elements (as defined by the human orexin NMR structures) as well as to align cysteines within the predicted orexin-A.


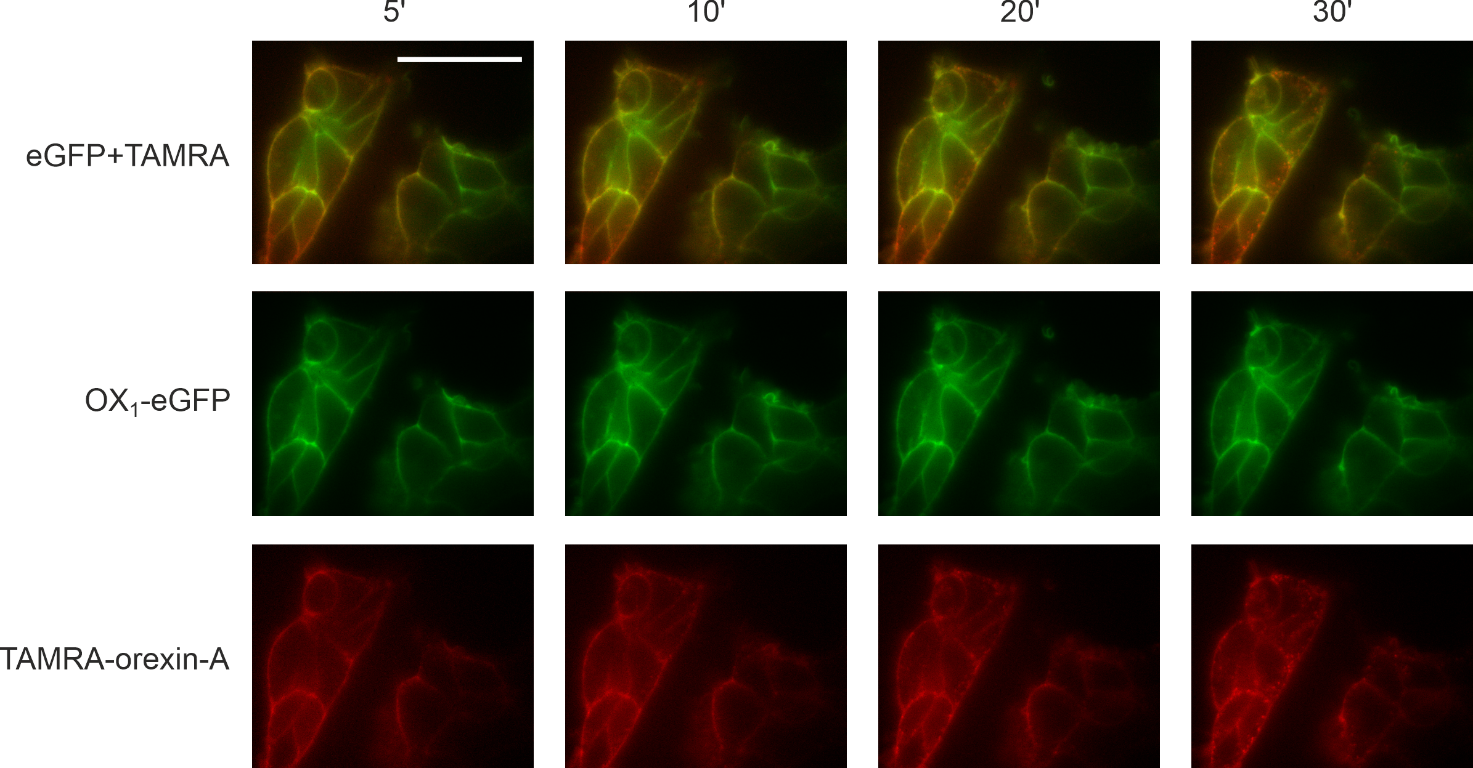


**Figure S7**. The binding of TAMRA-orexin-A in hOX_1_-eGFP-expressing cells. The panels from left to right show the binding of TAMRA-orexin-A at different time points (given on the top) after the addition. Scale bar (in white) 50 µm.


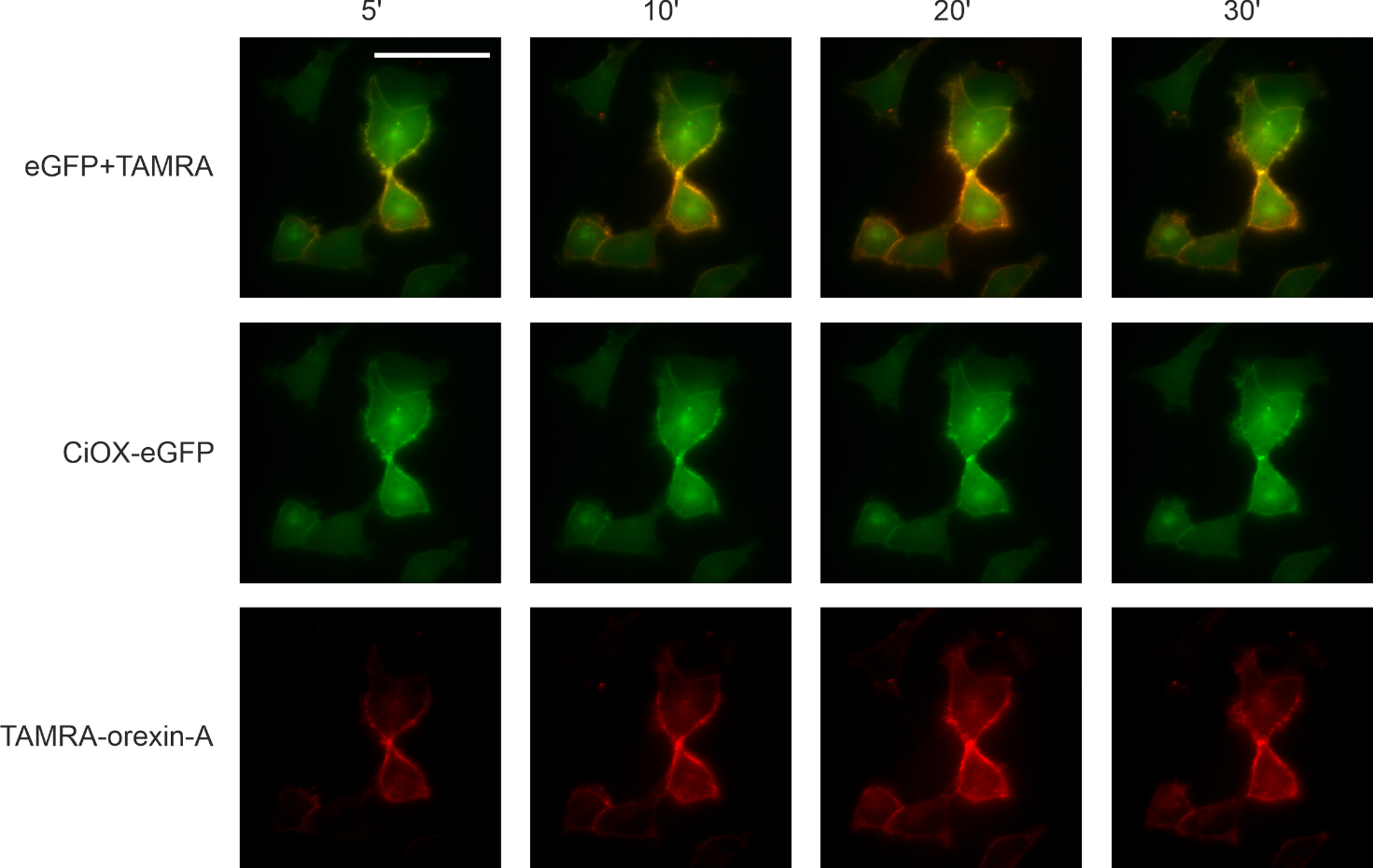


**Figure S8**. The binding of TAMRA-orexin A in CiOX-eGFP-expressing cells. The panels from left to right show the binding of TAMRA-orexin-A at different time points (given on the top) after the addition. Scale bar (in white) 50 µm.


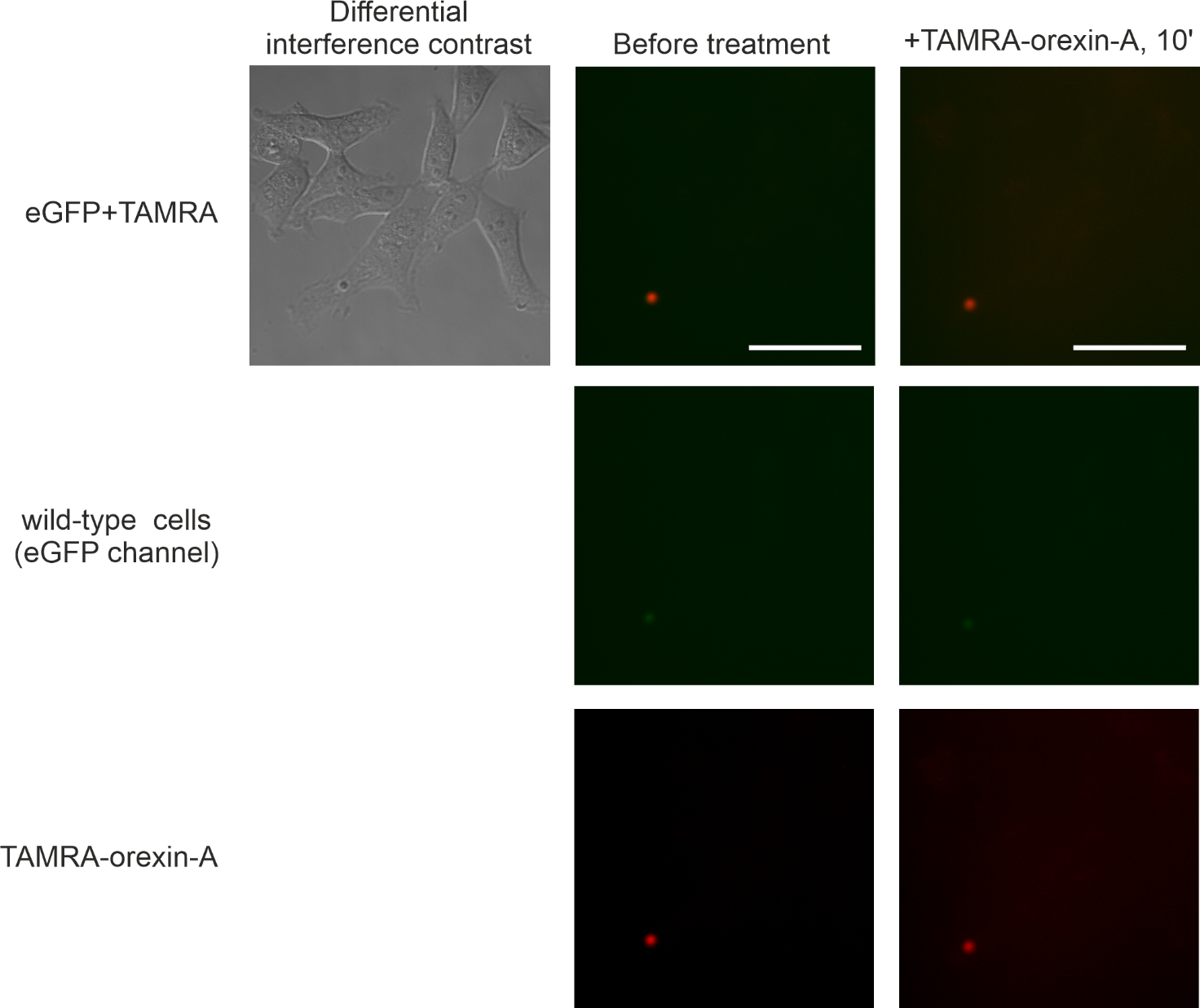


**Figure S9.** The binding of TAMRA-orexin-A in wild-type cells. The subfigures are organized in the same way as in Figure 7. A differential interference contrast image of the cells (a), untreated cells (b), the same cells after 10-minute incubation with TAMRA-orexin-A (c). No eGFP expression or plasma membrane binding of TAMRA-orexin-A observed. Scale bar (in white) 50 µm. The small red spot is an artefact.

**
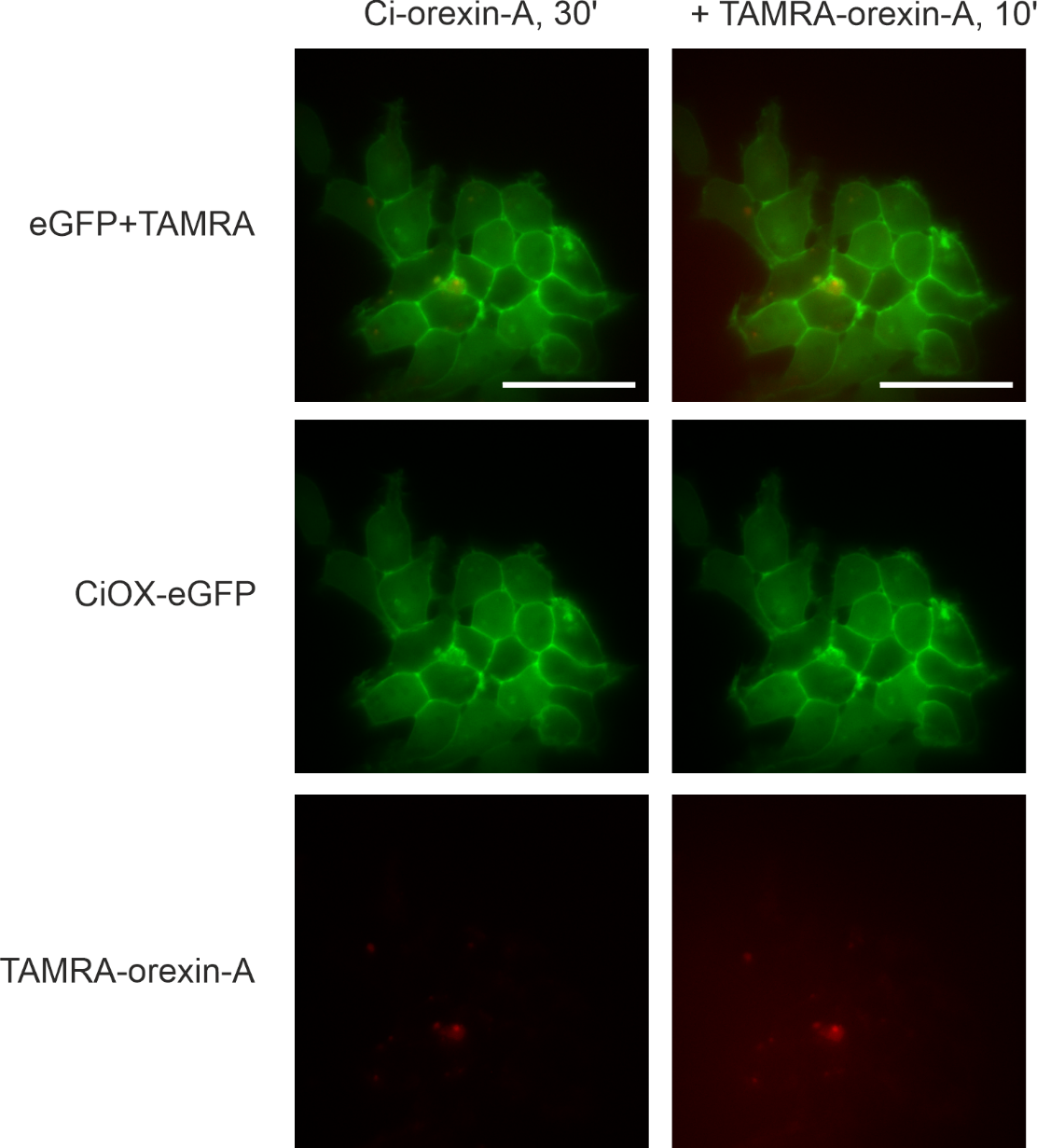
**

**Figure S10.** The effect of a 30 min pre-incubation with Ci-orexin-A on the binding of TAMRA-orexin-A to CiOX-eGFP cells. On the left, the cells incubated for 30 min with Ci-orexin-A (1 µM) and the same cells after an additional 10-min incubation with 30 nM TAMRA-orexin-A (right). No binding of TAMRA-orexin-A was observed; only some general red background from TAMRA in the medium is seen. The scale bar is 50 µm. The small red spots seen are artefacts.


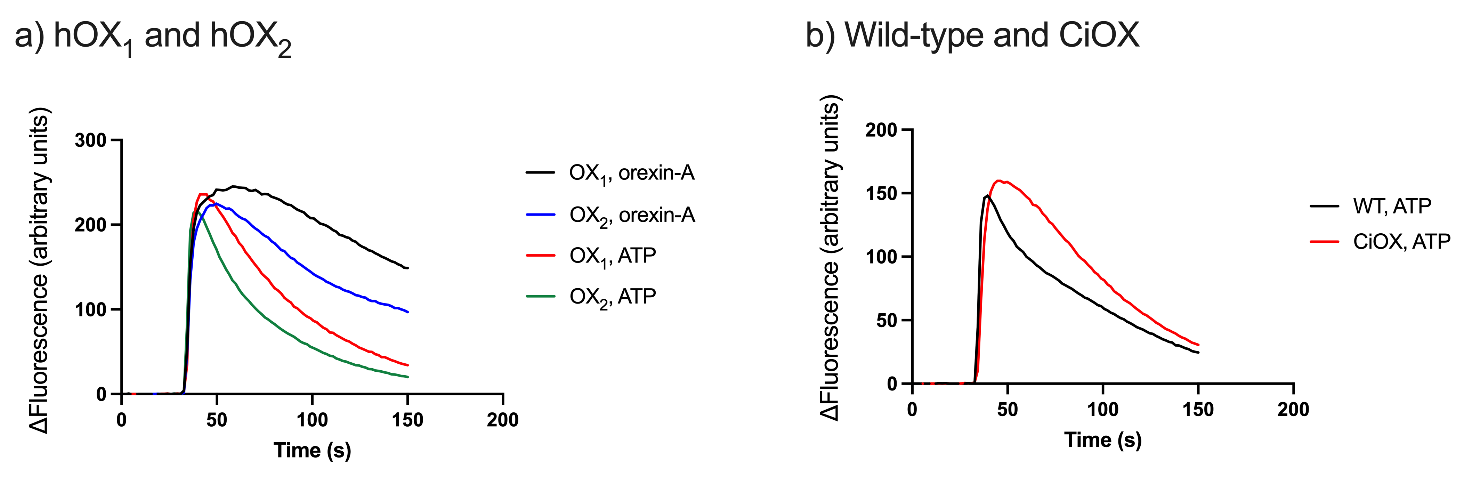


**Figure S11**. Ca^2+^ responses in Flp-In T-REx 293 cells. Fluorescence traces in ATP (100 µM) and orexin-A (10 nM) stimulated hOX_1_- and hOX_2_-expressing cells (a) as well as in wild-type and CiOX-expressing cells (b). Each subfigure represents a representative experiment (N = 1), and all the traces are means of triplicate wells.
