# Supplementary material for: Characterization of a putative orexin receptor in Ciona intestinalis sheds light on the evolution of the orexin/hypocretin system in chordates
Source: Sci Rep. 2024 Apr 2;14:7690. doi: 10.1038/s41598-024-56508-1 (PMC10987541; doi:10.1038/s41598-024-56508-1)
Supplement: Supplementary file 2 — Supplementary Information 2. [file 41598_2024_56508_MOESM2_ESM.docx]

>H._sap_OX1

-----------------------------------MEPSATPG-------------------------------------AQMGV--PPGSREP-SPVPPDYED---------------------EFLRYLWRDYLYPKQYEWVLIAAYVAVFVVALVGNTLVCLAVWRNHHMRTVTNYFIVNLSLADVLVTAICLPASLLVDITESWLFGHALCKVIPYLQAVSVSVAVLTLSFIALDRWYAICHPLL---FKSTARRARGSILGIWAVSLAIMVPQAAVME----CSSVLPELANRTRLFSVCDERWAD-DLYPKIYHSCFFIVTYLAPLGLMAMAYFQIFRKLWGR-QIPG-------------------------------------------TTSALVRNWKRPSDQLGDLEQGLSGEPQPR-----AR---A--------------------------------------FLAEVKQMRARRKTAKMLMVVLLVFALCYLPISVLNVLKRVFGMFRQA-----SDREAVYACFTFSHWLVYANSAANPIIYNFLSGKFREQFKAAFSCCLPGL-----------------------------------------------------------------------------------------GPCGSLKAPSPRSSASHKSLSLQSRCS--ISKISEHVVLTSVTTVLP-----------------------------------------------------------------------------------

>H._sap_OX2

-----------------------------------MSGTKLED--------------------------------SPPCRNWSSASELNETQEPFLNP-TDYDDE--------------------EFLRYLWREYLHPKEYEWVLIAGYIIVFVVALIGNVLVCVAVWKNHHMRTVTNYFIVNLSLADVLVTITCLPATLVVDITETWFFGQSLCKVIPYLQTVSVSVSVLTLSCIALDRWYAICHPLM---FKSTAKRARNSIVIIWIVSCIIMIPQAIVME----CSTVFPGLANKTTLFTVCDERWGG-EIYPKMYHICFFLVTYMAPLCLMVLAYLQIFRKLWCR-QIPG-------------------------------------------TSSVVQRKWKPLQPVSQP--RGPGQPTKSRM--------SA--------------------------------------VAAEIKQIRARRKTARMLMIVLLVFAICYLPISILNVLKRVFGMFAHT-----EDRETVYAWFTFSHWLVYANSAANPIIYNFLSGKFREEFKAAFSCCCLGV----------------------------------------------------------------------------------------HHRQEDRLTRGRTSTESRKSLTTQISNFDNISKLSEQVVLTSISTLPAANGAG---PLQNW----------------------------------------------------------------------

>M._mus_OX1

-----------------------------------MEPSATPG-------------------------------------AQPGV--PTSSGEP-FHLPPDYED---------------------EFLRYLWRDYLYPKQYEWVLIAAYVAVFLIALVGNTLVCLAVWRNHHMRTVTNYFIVNLSLADVLVTAICLPASLLVDITESWLFGQALCKVIPYLQAVSVSVAVLTLSFIALDRWYAICHPLL---FKSTARRARGSILGIWAVSLAVMVPQAAVME----CSSVLPELANRTRLFSVCDEHWAD-ELYPKIYHSCFFIVTYLAPLGLMAMAYFQIFRKLWGR-QIPG-------------------------------------------TTSALVRNWKRPSEQLEAQHQGLCTEPQPR-----AR---A--------------------------------------FLAEVKQMRARRKTAKMLMVVLLVFALCYLPISVLNVLKRVFGMFRQA-----SDREAVYACFTFSHWLVYANSAANPIIYNFLSGKFREQFKAAFSCCLPGL-----------------------------------------------------------------------------------------GPG---------SSARHKSLSLQSRCS--VSKVSEHVVLTTVTTVLS-----------------------------------------------------------------------------------

>M._mus_OX2

-----------------------------------MSSTKLED--------------------------------SLSRRNWSSASELNETQEPFLNP-TDYDDE--------------------EFLRYLWREYLHPKEYEWVLIAGYIIVFVVALIGNVLVCVAVWKNHHMRTVTNYFIVNLSLADVLVTITCLPATLVVDITETWFFGQSLCKVIPYLQTVSVSVSVLTLSCIALDRWYAICHPLM---FKSTAKRARNSIVVIWIVSCIIMIPQAIVME----CSSMLPGLANKTTLFTVCDEHWGG-EVYPKMYHICFFLVTYMAPLCLMILAYLQIFRKLWCR-QIPG-------------------------------------------TSSVVQRKWKQQQPVSQP--RGSGQQSKARI--------SA--------------------------------------VAAEIKQIRARRKTARMLMVVLLVFAICYLPISILNVLKRVFGMFTHT-----EDRETVYAWFTFSHWLVYANSAANPIIYNFLSGKFREEFKAAFS-CCLGV----------------------------------------------------------------------------------------HHRQGDRLARGRTSTESRKSLTTQISNFDNVSKLSEHVVLTSISTLPAANGAG---PLQNW----------------------------------------------------------------------

>G._gal_OX2

-----------------------------------MSGTQPEDV-------------------------------SPPCRDWTSSPELNETREPFLNPSADYDDE--------------------EFLRYLWKEYLHPKGYEWALIAGYIVVFIVALVGNVLVCIAVWKNHHMRTVTNYFIVNLSLADILVTITCLPATLVVDITETWFFGHHLCKAIPYLQTVSVSVSVLTLSCIALDRWYAICHPLM---FKSTAKRARNSIIIIWIVSCIIMIPQAIVME----CSSVFPGLANKTTLFTVCDEHWGA-EVYPKMYHTCFFLVTYMAPLCLMVLAYLQIFRKLWCR-QIPG-------------------------------------------TSSVVQKKWKSLQSSAQQ--RGLGQSTKSKI--------SA--------------------------------------VAAEIKQIRARRKTARMLMVVLLVFALCYLPISILNILKRVFGMFNHA-----DDRETVYAWFTFSHWLVYANSAANPIIYNFLSGKFREEFKAAFSCCIFGI----------------------------------------------------------------------------------------HSHHDERLTRGRASTESRKSLTTQISNFDNVSKHSEHVLLTNINTLTANGITATFSPLKSMELHLHIPGVNVSSNLDEAVRISAGCTGNTENAEWDKFVPSVTKLTSMELQQG------------------

>A._car_OX2

-----------------------------------MSAAEVEDG-------------------------------LLAYRNCSLNMELNGTREPFGKPTADYDEE--------------------EFLRYLWREYLHPKEYEWVLIAGYIIVFLVALIGNILVCVAVWKNHHMRTVTNYFIVNLSLADVLVTITCLPATLVVDITETWFLGDSLCKGIPYLQTVSVSVSVLTLSCIALDRWYAICHPLM---FKSTAKRARNSIIIIWIVSCIIMIPQAIVME----CSSMFPELANKTILFTVCDEHWGA-EIYPKLYHTCFFLITYMAPLCLMVLAYLQIFQKLWCR-QIPG-------------------------------------------TSSVVQRKWKPLQPGVQT--RGLRPSASLRI--------SA--------------------------------------VTAEIKQIRTRRKTARMLMVVLLVFALCYLPISILNILKRVFGMFNHA-----SDRETVYAWFTFSHWLVYANSAANPIIYNFLSGKFREEFKAAFSFCCFDVRRH-------------------------------------------------------------------------------------HHYHHDERVRGRISTESRKSLTTQISHFDHATKISEHVALSNINTLPPDGTAP----IHLW----------------------------------------------------------------------

>X._tro_OX2

-------------------MLSPERSTGSRQMNSSMQGAKLDD---------------------------------LLYRNWS-EQDVNGTQEPFLNPNADYDD---------------------EFLRYLWREYLHPKQYEWVLIVGYIIVFIIALIGNILVCVAVWKNHHMRTVTNYFIVNLSLADVLVTIICLPATLLVDITETWFFGKTLCKVIPYLQTVSVSVSVLTLSCIALDRWYAICHPLM---FKSTAKRAQQSIVIIWIVSCAIMIPQAIVME----CRSVFPELANKTILFTVCDERWEG-QIYSKVYHICFFCITYMVPLCLMILAYLQIFRKLWCR-QIPG-------------------------------------------TSSVVQKKWKPLQCSIQS--KGQ-QSTKSRN--------NA--------------------------------------VAAEIKQIHARRKTARMLMVVLLVFALCYLPISILNILKRVFGMFTHT-----NDRETVYAWFTFSHWLVYANSAANPIIYNFLSGKFREEFKAAFSCCCRGI----------------------------------------------------------------------------------------HNNQDDRLIRGRASTESRKSLTTQISNCDNVSRLSEHVVLTNINTLNANGSGA----VHNW----------------------------------------------------------------------

>D._rer_OX2

-----------------------------------MSGISVQR---------------------------------ACNSCFTSAQHLNSSADTISHSHAENEDD--------------------ELLKYIWREYLHPKQYEWVLIAGYILVFLVSLVGNTLVCFAVWKNHHMRTVTNYFIVNLSFADILVTITCLPASLVVDITETWFFGQTLCKILPYLQTISVSVSVLTLSCIAQDRWYAICHPLK---FKSTAKRARKSIVLIWLVSCIMMIPQAVVME----SSSLMPELTNKTSLFTVCDEQWPD-EIYPKVYHTCFFIVTYFAPLCLMVLAYIQICHKLWCQ-QIPG-------------------------------------------SSSVLQRQWKSLQCSAHA--VGSGESVKVRT--------ST--------------------------------------VSAEAKQVKARRKTARMLMVVLFVFALCYLPISILNIMKRVFGAFKNT-----GNRETVYAWFTFSHWLIYANSAANPIIYNFLSGKFREEFKAAFICQCSG-----------------------------------------------------------------------------------------RGETHKQRARGRTSTDSRKSLSTQVNNLDNISRISDQAV--------------------------------------------------------------------------------------------

>L._ocu_OX1

-----------------------------------MDRAQLNA-------------------------------------SAPGASEPNGTAQA-GGAHSDYEE---------------------EILRYLWKEYLFPRQYEWVLIAGYIFVFVVALTGNILVCLAVWRNHHMRTVTNYFIVNLSLADLLVTAICLPVSLVVDITESWFFGQTLCKVIPYLQTVSVSVSVLTLSFIALDRWYAICHPLM---FKSTARRARNSIVLIWLLSLAIMVPQAVVME----TSSMIPELANRTLLLSVCEERWGG-EVYPRVYHVCFFLVTYLAPLCLMFMAYFQIFRKLWSR-QIPG-------------------------------------------ASGAVSRKWVRGAGSSDDGGQAPGVERPSGSAGLVARPGTA--------------------------------------PSAEVKQLRARRKTAKMLLVVLLVFSLCYLPISVLNVLKRVSGVFDNA-----GDREAIYAWFTFSHWLVYANSAANPIIYNFLSGKFRGEFKAAFSCCFRGL-----------------------------------------------------------------------------------------RRCRGAEARRLARTTSQKSLTNGSKSEPLSSRVSEHVVLSSVRAVPS-----------------------------------------------------------------------------------

>L._ocu_OX2

-----------------------------------MSGVTANS---------------------------------VCEDCSPLLHEFNSSVESTHDPSVD-GDD--------------------ELLRYIWREYLHPKQYEWVLIAGYIIVFFISLIGNTLVCIAVWKNHHMRTVTNYFIVNLSFADVLVTITCLPASLVVDITETWFFGQTLCKVLPYVQTTSVSVSVLTLSCIALDRWYAICHPLM---FKSTAKRARKSIVIIWIVSCVIMIPQAIVME----CSSMVPELTNRTSLFTVCDEHWGD-EIYPKVYHICFFIVTYLAPLCLMVLAYIQIFHKLWCQ-QIPG-------------------------------------------TSSVVQRKWRSLQRSAQS--STPGESARIRT--------NA--------------------------------------AAAEIKQIRARRKTARMLMVVLFVFALCYLPISVLNVMKRVFGAFDNT-----SDREAVYAWFTFSHWLIYANSAANPIIYNFLSGKFREEFKAAFSCCCCEI----------------------------------------------------------------------------------------RSPKEEHQIRGRTSTDSRKSLTTQLSNFDNVSRISEQLVLTSMGTLRSNDGDKT-----TW----------------------------------------------------------------------

>H._sap_NRFFR

-----------------------------------MEGEPSQP--------------------------------------------PNSSWPLSQNGTNTEATP--------------------ATNLTFSSYYQHTSPVAAMFIVAYALIFLLCMVGNTLVCFIVLKNRHMHTVTNMFILNLAVSDLLVGIFCMPTTLVDNLITGWPFDNATCKMSGLVQGMSVSASVFTLVAIAVERFRCIVHPFR---EKLTLRKALVTIAVIWALALLIMCPSAVTLTVTREEHHFMVDARNRSYPLYSCWEAWPE-KGMRRVYTTVLFSHIYLAPLALIVVMYARIARKLCQA---PG----------------------------------------------------------PAPGGE----------------------------------------------------------EAADPRASRRRARVVHMLVMVALFFTLSWLPLWALLLLIDYGQLSAPQ------LHLVTVYAFPFAHWLAFFNSSANPIIYGYFNENFRRGFQAAFRARLCPRPS------------------------------------------------------------------------------------------GSHKEAYSERPGGLLHRRVFVVVRPSDSGLPSESGPSSGAPRPGRLPLR-----------------------------NGRVAHHGLPREGPGCSHLPLTIPAWDI---------------------

>H._sap_QRFPR

-----------------------------------MQALNITP------------------------------------------EQFSRLLRDHNLTREQFIALY--------------------RLRPLVYTPELPGRAKLALVLTGVLIFALALFGNALVFYVVTRSKAMRTVTNIFICSLALSDLLITFFCIPVTMLQNISDNWLGGAFICKMVPFVQSTAVVTEILTMTCIAVERHQGLVHPFKM-KWQYTNRRAFTMLGVVWLVAVIVGSPMWHVQQ------LEIKYDFLYEKEHICCLEEWTS-PVHQKIYTTFILVILFLLPLMVMLILYSKIGYELWIKKRVGD--------------------------------------------------------GSVLRTIH----------------------------------------------------------GKEMSKIARKKKRAVIMMVTVVALFAVCWAPFHVVHMMIEYSNFEKEY------DDVTIKMIFAIVQIIGFSNSICNPIVYAFMNENFKKNVSSAVCYCIVNKTFS-------------------------------------------------------------------------------------PAQRHGNSGITMMRKKAKFSLRENPVEETKGEAFSDGNIEVKLCEQTEEKKKLKR------------------------HLALFRSELAENSPLDSGH---------------------------------

>H._sap_GALR2

---------------------------------------------------------------------------------------MNVSGCPGAGNASQAGGG------------------------------GGWHPEAVIVPLLFALIFLVGTVGNTLVLAVLLRGGQAVSTTNLFILNLGVADLCFILCCVPFQATIYTLDGWVFGSLLCKAVHFLIFLTMHASSFTLAAVSLDRYLAIRYPLHS-RELRTPRNALAAIGLIWGLSLLFSGPYLSYYR------------QSQLANLTVCHPAWSA--PRRRAMDICTFVFSYLLPVLVLGLTYARTLRYLWRA------------------------------------------------------------------VDP----------------------------------------------------------VAAGSGARRAKRKVTRMILIVAALFCLCWMPHHALILCVWFGQFP---------LTRATYALRILSHLVSYANSCVNPIVYALVSKHFRKGFRTICAGLLGRAPG---------------------------------------------------------------------------------------------RASGRVCAAARGTHSGSVLERESSDLLHMSEAAGALRPCPGAS----------------------------QPCILEPCPGPSWQGPKAGDSILTVDVA-------------------------

>H._sap_ENDRB

-MQPPPSLCGRALVALVLACGLSRIWGEERGFPPDRATPLLQTAEIMTPP-------------------------TKTLWPKGSNASLARSLAPAEVPKGDRTAGS---------------PPRTISPPPCQGPIEIKETFKYINTVVSCLVFVLGIIGNSTLLRIIYKNKCMRNGPNILIASLALGDLLHIVIDIPINVYKLLAEDWPFGAEMCKLVPFIQKASVGITVLSLCALSIDRYRAVASWSRI-KGIGVPKWTAVEIVLIWVVSVVLAVPEAIGFD----IITMDYKGSYLRICLLHPVQKTAFMQFYKTAKDWWLFSFYFCLPLAITAFFYTLMTCEMLRK-----------------------------------------------------------------KSGM----------------------------------------------------------QIALNDHLKQRREVAKTVFCLVLVFALCWLPLHLSRILKLTLYNQNDPNR--CELLSFLLVLDYIGINMASLNSCINPIALYLVSKRFKNCFKSCLCCWCQSFE----------------------------------------------------------------------------------------------EKQSLEEKQS---------CLKFKANDHGYDNFRSSNKYSSS----------------------------------------------------------------------------------

>B._jap_OX

-----------------------------------MSNNSTAA-------------------------------------ATEGVFNMTEDVYPTWLL-PTPPDY--------------------DLEDYLL-DYLYPKHYEWALIIAYILVFLLALIGNGLVCFVVIRNSHMRTVTNYFIANLSAGDLLVTIICLPPTLVVDIMETWFFGETMCKIIPYLQMVSVSVSVLTLCAIAVERWYAIVHPLK---FKSTNARARTIICLIWVVSLSIMAPLIPMYK----TNRTMP--AEKTDLMMVCDEHWPD-PIYGKIYHAAIVMVLFGVPIVLMMVSYCMIVWKLWSD-QVPG-ISSSTS----------------------------LRAPTRSNDSQRIKT---PVSRSTSDNIVLSTSSSTAA-VVTASSSFHSGTGSIA--------------------------------DKTAENTVQSRRKVARMLVAVVVVFAICYIPLMILTFLKRVYGFFELM-----NDRSGLYAAFTVSHWLLYLNSAINPLIYNFMSEKFRSEFKASLPCCFPEAAR--------------------------------------------------------------------------------------KKR-EARGMTVGRPTLSRMHTTRTTGTTELLSRFESTATTRYPSTYEFTFARDRNEFGLSRFDSRPLPKSPILLHDTMKPRDLPKITYDAIQQDTDLRMISEHTSEPVAEPSEISCIVPTLCTNDIPNQIE

>B._bel_OX

---------------------------------------------------------------------------------------MSEDVYPTWLLLPTPPDY--------------------DFESYLL-DYLYPKHYEWALIVGYILVFLLALIGNGLVCFVVARNSHMRTVTNYFIANLSAGDLLVTIICLPPTLVVDIMETWFFGETMCKIIPYLQMVSVSVSVLTLCAIAVERWYAIVHPLK---FKSTNARARTIICLIWVVSLSIMAPLVPMYK----TSRFYP--EHKTDLMTVCDEHWPD-PIYGKIYHAAIVMVLFGIPIVLMMVSYCMIVWKLWSD-QVPG-ISSSTS----------------------------LRAPSRSSDSQRIKT---PVSRSTSDNIVLSTSSSAAATVVTASSSFHSGTGSIA--------------------------------DKTAENTVQSRRKVARMLVAVVVVFAICYIPLMILTFLKRVYGFFDVT-----NNRQGVYAAFMVSHWLLYLNSAINPLIYNFMSEKFRSEFKASLPCCFPEAAR--------------------------------------------------------------------------------------KKR-EARGMTVGRPTISRMHTTRTTGTTELLSRFES--TTRYPSTYEFAFARDRNEFGLSRFDTRPIPKSPILPHDTMKPRDLPKSTYETTQQDTDLRMISEHTSEPTAEPAEISCIVPALCTNDLPKQIE

>S._pur_OX

-------------------MAGDYYGHTNLSQPHHLSSSVN-----------------------------------------------GSTSSEWDYD-DYIQG---------------------IYDQTRKR--VYPEIHEYFLIAIYFIIFFVAIVGNSMVCIAILKNDHMRTVTNYYIMNLATTDIMIAVVCLPITITVDVSESWFFGQTACYLIPYFQLVLVCASIYTLMMIAVDRYLAICHPLK---FQIRASRTLLTIALVWVVSFFIALPVAVVNGL--ESQPASVHIGKPLWRMSCTESRWVS-KVWEKLYHTAFFLAVYIVPLAVIGVAYTRVCRRLWSG--IPTEEG------------------------------------------HGASKPSFNQANVVSTTT----------------------------------------------------------ISKSTEAQLKSRRKVASMLIVVVVTFAICFFPFQLLNVLKKHNAFGNLRDASSSAQYNAVYIPYIIGHLMAFINSAINPIIYNFMSAKFRQAFKSMFD-CLPCCR----------------------------------------------------------------------------------------------SPRQSPAIDGT-----------SAG-PAYRRANSTGVSDTHTT------------------------------------EYVPMTSIRNGRGVNNNISAMSKGV--------------------

>S._kow_OX

-------------------MAYDCIWSGNLSYYNSTEGDISLFP-----------------------------------------CGDNDTSSENAYDWDYYYD---------------------VKLPTRLRNFVYPAPHEWFLISVYALVFLMALVGNVLVCFAVLRNQQMRTVTNYYIVNLSFADILVSLICLPVTVTFETTETWYFGDLACKIIPYVQVVSMSVSVLTLSAIAVDRYFAICQPLL---FKSTAKRTLTIIFSIWLVSFIIPIPQAIVY----ETEAADTYKRTYIYFTKCYEKVWFG-TIQQKIYHVALVLVIYVIPLLLIGIAYLFICRQLWAT--IPGTMP------------------------------------------SGGGKHCHSNG-----SD----------------------------------------------------------MNRTTMSQLKSRRKVANMLIIVAILFALCYLPLHLLNIIRQFPIF----DEVVQEDRHSFHIPFLVAHWLAFANSATNPVVYNFLSAKFRKEFKAAFTCCLSCCA----------------------------------------------------------------------------------------------RKRRRRHKRGG-----------YQGSIMHSSMASTSKSYGNCT------------------------------------EHISMSTVRTGVHV-------------------------------

>M._sex_ATR

-MLNKSINISILLLIIVESSTSEIIEDNITREPIKATELNRRIIRLIEIK---------------------ESNEDLPNRYKRSLPENDKEPPETKENTTEE-----------CVGAAEFCNMTKEAYIAMLQEYIYPQTYEWVLIATHSIVFLTGLIGNALVCIAVYRNHSMRTVTNYFIVNLAVADFMVILFCLPATVLWDVTETWFLGDVLCKMLLYFQSVSVTVSVLTLTFISVDRWYAICFPLK---FKSTTSRAKTAILIIWILSLSFNSPDLVVL----KTDKP-VPLRFELEYLVQCIATWSS-QA-DPVWHILKVVFIYTIPLLLMTVAYLQIVRVLWHSDKIPGQAE-------------------------------------------------------TIKLA------------------------------------------------------------PAEQTQLRSRRKAAKMLVAVVVMFAVCYFPVHLLSVLRYTLDME---------QSDAITFLALVSHVMCYANSAVNPLIYNFMSGKFRREFRRAFCCSSRPVH--------------------------------------------------------------------------------------------ENFTSLTRVTTS------------KKK---EQSCDKSLSQRNVSN------------------------------------TTFIQNGFKSGYYA-------------------------------

>S._gre_ATR

MTENETDYYSQWESALNESNASEATTSSPLYLAWWTLSPSSNVT---------------------------ATTLVVNASTPDFSLDEDGNATEGQNCTNDY-----------CIPD--------IDYWNMVYQHVYPKDYEWILIAMHSLVFVAGLVGNALVCLAVYRNHAMRTVTNYFIVNLAVADFMVILFCLPPTVLWDVTETWFMGTGLCKVVLYLQTVSVAVSVLTLTFISVDRWYAICFPLR---FNSTTGRAKTAIAIIWLLALAFDIPELVVL----RAR----GRDWDSVLLTQCEGSWSY-DS-EMVFHGAKSLLLYTLPLLFMSVAYFQIVRVLWRSDNIPGHDDHNGDVIS-----------------------------SKEAGHHATFAPSGSVGSRRVPMAG----------------------------------------------------------NSTTEAQLRSRRKAAKMLVAVVAMFAICYLPVHLLNILRYTVDIP---------QNDTTSAISMLSHWLCYANSAVNPVIYNFMSGKFRAEFRRLFWTCAYGS---------------------------------------------------------------------------------------------NRYSPAPGAAPS-------------AAMVARHGGARQ--RPGGGG------------------------AASSAGHEMRSLYRRGPGP--------------------------------------

>A._aeg_ATR

-------MSVRIDRSLEPSSEGKPPAMTTSNFNGAICRDGNNVGTEAEQGSGSCALVNNGTKSPLAAGLDGNQTVVTPYYTIVNLDNHNDVLCDEEYDTEEYNENCFIDHNVTCVGDPLYCNLTYDEYRQLLMDYIYPSTAEWILIASHSVVFIMGLVGNALVCIAVYTNHSMRTVTNIFIVNLAVADFFVILFCLPPTVVWDVTETWFMGKAMCKVVIYFQTVSVTVSVLTLTYISIDRWYAICFPLR---YKPRPERAWRFIAVIWLIGFLSDLPEFLVL----TTRR--KKLRFDIKLFTQCVSTWDN-EK-EKTFYIVKFVFLYSLPLLFMTIAYFQIVRVLWRSDTIPGHRE------------------------------------SRTQPY------GIHSTRTTLNCVG----------------------------------------------------------NTSTMGQLRARRKAAKMLVAVVVMFASCYFPVHMLNVARYTFDIG---------QSDVVAVLSLFSHWLCYANSAVNPVIYNFMSGKFRREFKNALEKCHCLRNPRGLGGRVGGYDDRSMYHTATRMNASPSSRSNYHLTSVRNISIKHTQQTSFNNGSRHHHARNSINHPGSLTGAPQISPVSFEERMALTKNMDGNIGCGDPTMAGTATSVASRAEGNSSGHVGANSNTNHHHLHHHGV------------------------ACNGSTPDAPATTTGTAPPTNGGSSSMLMIVNKSSNCKINGT---------------

>F._occ_ATR

-------MAVTSPPLLD--NDSAPDGWNGTYFN-DSCRN----------------------------------------------------------YTNDY-----------CVPD--------EEYLDMMLEYIMPTKMEWVIIAMHCAVFIGGLVGNALVCLAVYRNHTMRTVTNYFIVNLAVADFLVILMCLPPTVLWDVTETWFMGTALCKIVLYFQTVSVTVSVLTLTTISVDRWYAICFPLK---FKSTTSRAKKAIIIIWLLALSFDVPELVVL----ETKR--KALGIDTIFFTQCLPTWGD-SS-ETTYHCVKTLFLFFLPLAFMTVTYVQIVKVLWSKTNIPGHAE------------------------------------TKSLSYQYCNGNGVSGTRRTMHR------------------------------------------------------------SISASSQILSRRKAAKMLVVVVLMFFICYLPVHLLSILRYTMIIP---------QTELMTVTAMFVHWLCYANSAVNPLIYNFMSGKFRGEFRLAFQQCACES--WVLGG-----------------------------DTVR---------------------------PAAGGGGP-----------------GG--GPGPGALAG-----------VSPGHVVLGMSQLHRRPAPRAA------------------------PRAASTLAATATTTLSVSVLDSGP---------------------------------

>H._arm_ATR

--MNFDKKISIFIIGLIILTSVEGIHAEETRTGIKNNETKSRHNKTIEDL---------------------TAEN----------ATASDEP--SKENATEV-----------CVGQKEFCNLSKEEYVSMLNNYIYPHTYEWVLIGTHTLVFITGLVGNALVCVAVYRNHSMRTVTNYFIVNLAAADFMVILFCLPATVVWDVTETWFLGDVLCKMLLYFQSVSVTVSVLTLTFISVDRWYAICFPLK---FKSTTGRAKTAILIIWTLSLIFNAPELVVL----TTEKS-VPLRFELEYLVQCVATWSS-NS-DLVWHIIKVIFIYTLPLLLMTVAYYQIVKVLWRSEKIPGHAE-------------------------------------------------------TMKL------------------------------------------------------------APAEQTQLRSRRKAAKMLVAVVIMFAVCYFPVHLLSVLRYTLDME---------QNDVITCLALISHVMIYANSAINPLIYNFMSGKFRREFRRAFCCSTASDL--------------------------------------------------------------------------------------------ENFTTLSRITTS------------KKRPCALMTFETKTQGRNVCS------------------------------------TTFVHSSYKDRLT--------------------------------

>D._ple_ATR

--MAIKIILALAVLIIYFHKNDAKIRFNGLQEDFMIESHNNDIFGEDTFL---------------------RLKRSVEQDKKLLIGDNNKSKNEIESNSSEP-----------CVGDAEFCNMTREDYIQMLYEYIYPQTYEWVLIGVHTTVFVIGLIGNLLVCLAVYRNHAMRTVTNYFLVNLAVADFMVLLFCLPATVLWDVTETWFLGDALCKILLYIQSVSVTVSVLTLTFISVDRWYAICFPLK---FKSTINSAKTAILVIWALSLVFNTPELVVL----TTVKV-VPLRFDLEYLVQCTATWSY-SS-DLIWHIIRIVFVYTVPLLLMTVAYHQIVRVLWSSQKIPGLAE-------------------------------------------------------TMKL------------------------------------------------------------ASAEQIQLQSRRKAAKMLVAVVVMFAVCYFPVHLLSVLRY-LDME---------QNDMITCLALVSHVLCYVNSAINPLIYNFMSGKYRREFRRVFCCNQNLTR--------------------------------------------------------------------------------------------NTFTTMTRLTTS------------RKKYETADKTQRSSLKFHKCENMALRHHNCGLALKSQCGHIALNERVNELNQGFRVCENVMNNGQRCSIKAIGF----------------------------

>B._ter_ATR

-----------------------MHPLELVIVGWLASVISTLVD---------------------------AIDYLDDYSAMDYT-DESDIDYNATNCTNSY-----------CISN--------EEYVDRMINYIFPKFWDWVLIASHSVVFVVGLVGNALVCIAVYRNHSMRTVTNYFIVNLAVADFLVLLLCLPFTVLWDITETWFLGLTLCKAVPYLQTVSVTVSILTLTFISIDRWYAICFPLR---FKSTTGRAKSAIIGIWAAALLFDIPDLVVL----HTVPP-THIKIKTVLFTQCDISWSQ-RS-QVAFTIVKLIFLYTGPLIFMSVAYWQIVKVLWRS-NIPGHN-----------------------------------------------LPSRASQMSQIPSTGGG----------------------------------------------------------NPEVQLRSRRKAAKMLVTVVITFAICYFPVHLLSVLRYTTTLP---------SNKWINAISLIAHGLCYFNSAVNPLIYNFMSGKFRKAFRRTFRCARENG---------------------------------------------------------------------------------------------SRIQRGYLASTSN-----------FPRIKSRTTTIRTTFKNNNNL------------------------QRNTEIIPLSAITTIQQNEKHD-----------------------------------

>P._dum_ATR

----MAVRNYSVDNPSVLEIDAMHP----------AYEDGD--------------------------------------------FGITETPPNATNCRNEY-----------CVSD--------EEYLDMIKAYVFPSRFEWVLIVLYIQVFTIGLCGNLLVCFAVWRNQHMRTVTNYFIVNLAVADLLVIIICLPPTVLVDVSETWYMGAVMCKVVHYMQGVSVSVSVLTLSCISVERWYAICHPLT---FRSTTTRVRSIIVVTWVVALVILIPELIVL----DTSS---KYENLTILLTVCRPTMLP-FYNPMAYELFKMVALYFLPIILMSVTYGNIVICLWSN-AIPCEPT-------------------------------------------------TASS-RPLHNNSRT----------------------------------------------------------TAEAQLIARRKAAKMLIAVVVMFGVCYLPVHLTNILRYAKLLP---------ESENITFFPLVAHWLCYFNSAINPVIYNFMSARFRNEFKHACSCCTRVWC----------------------------------------------------------------------------------------------RDVRLRRRQG----------------DSMYSYRYTN---DMSQ-----------------------------------TEQMTLTTIRPDINHVDE----------------------------

>C._int_OX_(X2)

-----------------------------------MNSRALTS------------------------------------TVLTAGRKFSEANYSVGINSTAVSIN--------------------QLDFDIWEYYLKPTNAEWFVMSLYVLVFLISIIGNCLTIAFILRRKHLRTTINYFMLNLALADIMVTIICLPPTLMVDFMESWLVGQFLCKFTPYLQMAVTSVSSLSLGAIAVNRWFVVCHPLKVARTRRSAKHALLTMTSIWLFSLITLCPIIFVTE----LTEDFPG-YKELNLLKSCGEHWTT-FLHQAVFHIYYVTVCYALPLMVMAIAYTNVFRKLSYT-KIPGHVSRETNPIPKRRGQCHSCSSNSEHTHRGSTIGSEPNSPSKSNPSSPTAKKQDGASVGQEHVRNGIDLPMPREQESLFYTGAWKVKRDSEDYKKLYLQRNRNSSTFSKLITSRKTQRTYCRKCKIKRNLIQSRKRSGRIQVALVVVYFLCYSPAMVLDLIRRTSDLFTS------VHRESTYFLFAIAHLLVYLNSALNPIIYNCFSVRVSGEILFVQRPRFARS---------------------------------------------------------------------------------------------------KTPR-TVQAVI-------------------------------------------------------------------------------------------------------------

>C._int_OX_(X1)

-----------------------------------MNSRALTS------------------------------------TVLTAGRKFSEANYSVGINSTAVSIN--------------------QLDFDIWEYYLKPTNAEWFVMSLYVLVFLISIIGNCLTIAFILRRKHLRTTINYFMLNLALADIMVTIICLPPTLMVDFMESWLVGQFLCKFTPYLQMAVTSVSSLSLGAIAVNRWFVVCHPLKVARTRRSAKHALLTMTSIWLFSLITLCPIIFVTE----LTEDFPG-YKELNLLKSCGEHWTT-FLHQAVFHIYYVTVCYALPLMVMAIAYTNVFRKLSYT-KIPGHVSRETNPIPKRRGQCHSCSSNSEHTHRGSTIGSEPNSPSKSNPSSPTAKKQDGASVGQEHVRNGIDLPMPREQESLFYTGAWKVKRDSEDYKKLYLQRNRNSSTFSKLITSRKTQRTYCRKCKIKRNLIQSRKRSGRIQVALVVVYFLCYSPAMVLDLIRRTSDLFTS------VHRESTYFLFAIAHLLVYLNSALNPIIYNCFSVQFRKEFRLTFNCCFSSS---------------------------------------------------------------------------------------------------SQRRNSVRSTAALRSLEDTKDRTSCDMML-------------------------------------------------------------------------------------------

>C._sav_OX

----------------------------------------------------------------------------------------------------------------------------------IWEFYLKPTQTEWFVISLYAIVFLTSIVGNCLTIAFILRRKHLRTTINYFMLNLALADIMVTIICLPPTLMVDFMESWLVGQFLCKFTPYLQMAVTSVSSLSLGAIALNRWFVVCHPLRVARSRRSAKNSLISMACIWLFSLITLCPVVFVTE----LTDDFPG-YSELELLKSCGEHWAT-FLHGAVFHVYFVTVCFALPLVVMAIAYTSVFRKLSRT-KVT---KRTVSQLFKRLG-----------------------------ESIPTFKRG---------LRAGFADKVESQQS------------DSE-----------------------------------KPNLISSRKRSGRIQVALVIVYFLCYSPAMVLDVIRRTSNVFDS------VHRESTYFMFAIAHLLMYLNSALNPIIYNCFSVQFRKEFRLTFSCCFP-----------------------------------------------------------------------------------------------------------------------------------------------------------------------------------------------------------------------------

>C._gig_ATR

MINMKESQNHTREKTDVGTEDAIALFIVKEHFEKLMMENLTNIP--------------------------------------ANLSGTRDNSSVPIECTDIF-----------CRPD--------EEYLDYLEDYVFPDDWEWGIIILYALTFIVGLSGNVLVCFAVWRNRSMRTVTNIFIVNLAIADLAVIIICLPPTLLSDVTETWYFGFAMCKIALFLQTTSVAVSVFTLSAISVERWYAICYPLR---FKSTKRRAKIIILVIWIIAFLLALPEVIVA----DLTR--FVKRQYIDLLIFCGPQWSD-KTNQVVYQSVIIVLMYLLPLVLMTVTYSMIAVVLWTG-KIPGAIE-------------------------------------------------SAN--RPMMDGN----------------------------------------------------------VNRAEEQLESRKKAAKMLITVVIGFAVCYFPVHLFNILRYADALRF-------VAPRMIQVLSMISHWLPYLNSSINPIIYNFMSAKFRKEFTAACCCTKRRRA----------------------------------------------------------------------------------------------FSVHYKSGVS----------------TFSCASQYTHRNNSNSC-----------------------------------TEQVLLSTYPDH----------------------------------

>O._bim_ATR

------------------------------------MDNST--------------------------------------------GDVLSDESSTAPCYNVY-----------CLSD--------EDYINMVEQHVQPNAGEWILVVIFIILFIVGLVGNFLVCYAVIKNSQMRTVTNLFIMNLAIADFMVILICLPSSLLVDVSETWFFGEVMCKIFLYLQTVSVAVSVLTLSAISIERWYAICHPLS---FKSTASRARNIILTIWLLSACVASPDLVTA----RTYRSLPMRYNYVKWLVSCRPSWTQ-RS-QFIYQMFLFIALYFLPFCLMAFTYTRITLVLWRE-DIPGVNE-------------------------------------------------TAGGHRLMAENR----------------------------------------------------------NPNTNAQLQTRRKAAKMLITVVIVFGICNLPVHILNIVRYANIS---------NNLKAISIFSLISRLLCYVNSAINPIIYNFMSAKFRKEFKSVCLCCVSPLE----------------------------------------------------------------------------------------------QEQHTQRPKS----------------GGSYNISYSR---TNCQ-----------------------------------TEQFTLISVKE-----------------------------------

>O._myk_OX2

----------------------------------MMSGITVNS---------------------------------DCVECSP-PSHEPCTTELHPYSSID-DDD--------------------ELLKYIWREYLHPKQYEWVLIVGYIIVFFVSLIGNTLVCFAVWKNHHMRTVTNCFIVNLSFADVLVTITCLPASLVVDITETWFFGNTLCKILPYLQTISVSVSVLTLSCIALDRWYAICHPLM---FKSTARRARKSILLIWGVSCIIMIPQAIVME----CSSLLPELTNKTSLFTVCEERWGA-DVYPKVYHTCFFIVTYFAPLCLMVLAYIQICHKLWCQ-QIPG-------------------------------------------TSSVLQRKRTPLQGSTYS--PGPGESARVRTST----------------------------------------------VSAEIKQVRARRKTARMLMVVLFVFALCYLPISVLNIMKRVFGTFKYT-----NSRETVYAWFTFSHWLIYANSAANPIIYNFLSGKFRAEFKAAFSCRSFG-----------------------------------------------------------------------------------------RCQNQTEGIRRRMNTDSRKSLSTQVNNMDNVSRISDHVV--------------------------------------------------------------------------------------------

>C._mil_OX2

---------------------------------MAIPGTRAAP--------------------------------EDLRLDSNVSVSMSPTTNTSAQSTADYDD---------------------EFIRYLWGEYLYPKEYEWVLIAAYIAVFVVALVGNVLVCVAVWKNHHMRTVTNYFIVNLSFADVLVTIICLPASLVVDITETWFFGQIFCKVIPYLQTVSVSVSVLTLSCIALDRWYAICHPLM---FKSTAKRARNSIIIIWIVSCVLMTPQAIVME----CSIMVPELVNKTILFTVCDEHWGD-TVYPKVYHICFFIVTYMGPLCLMILAYFQIFRKLWCR-QIPG-------------------------------------------TSSVVQRKWKPLALQCSSQ-RAAGLQTKPRINA----------------------------------------------VAAEIKQIRARRKTARMLMVVLFFFAVCYLPISILNVLKRVFGMFDNI-----DDRATVYAWFMFSHWLVYANSAANPIIYNFLSGKFREEFKAAFS-CSFGT----------------------------------------------------------------------------------------GQQREEGLRRGRASTESRKSLTTQICHLDNPSKISEQVVLTSLNTLQGSHLAS----NRNW----------------------------------------------------------------------

>L._cha_OX2

-----------------------------------MAWIMLDS---------------------------------LYGNITSAALDLNTTQEPSPNPVSDYDD---------------------EFLRYLWNEYLHPKEYEWALIAGYIIVFIVALFGNILVCVAVWKNHHMRTVTNYFIVNLSLADVLVTITCLPASLVVDITETWFFGQTLCKVIPYLQTVSVSVSVLTLSCIALDRWYAICHPLM---FKSTAKRARNSIIIIWIVSCVIMIPQAIVME----CSNLVPELANKTNLFTVCDEQWGD-EIYPKVYHICFFLVTYMAPLCLMILAYLQIFHKLWCR-QIPG-------------------------------------------TSSVVQRKWKPLQCSLQP--RGSGFKNKPKVNA----------------------------------------------VAAEIKQIHARRKTARMLMVVLFIFALCYLPISILNILKRVFGMFKSS-----NDRETVYAWFTFSHWLVYANSAANPIIYNFLSGKFREEFKAAFSCCCLAT----------------------------------------------------------------------------------------QTHQEERLSRGQASTESRKSLTTQVTNFDHASRVPEHVTLTSIITQPTNGNIS----QQSW----------------------------------------------------------------------
