# Supplementary material for: Characterization of a putative orexin receptor in Ciona intestinalis sheds light on the evolution of the orexin/hypocretin system in chordates
Source: Sci Rep. 2024 Apr 2;14:7690. doi: 10.1038/s41598-024-56508-1 (PMC10987541; doi:10.1038/s41598-024-56508-1)
Supplement: Supplementary file 3 — Supplementary Information 3. [file 41598_2024_56508_MOESM3_ESM.docx]

>H._sap_OX1

YEWVLIAAYVAVFVVALVGNTLVCLAVWRNTVTNYFIVNLSLADVLVTAICLPASLLVDITEGHALCKVIPYLQAVSVSVAVLTLSFIALDRWYAICHTARRARGSILGIWAVSLAIMVPQAAVMLYPKIYHSCFFIVTYLAPLGLMAMAYFQIFRKLWGR-QIPFLAEVKQMRARRKTAKMLMVVLLVFALCYLPISVLNVLKRVFDREAVYACFTFSHWLVYANSAANPIIYNFLSGKFREQFKAAF

>H._sap_OX2

YEWVLIAGYIIVFVVALIGNVLVCVAVWKNTVTNYFIVNLSLADVLVTITCLPATLVVDITEGQSLCKVIPYLQTVSVSVSVLTLSCIALDRWYAICHTAKRARNSIVIIWIVSCIIMIPQAIVMIYPKMYHICFFLVTYMAPLCLMVLAYLQIFRKLWCR-QIPVAAEIKQIRARRKTARMLMIVLLVFAICYLPISILNVLKRVFDRETVYAWFTFSHWLVYANSAANPIIYNFLSGKFREEFKAAF

>M._mus_OX1

YEWVLIAAYVAVFLIALVGNTLVCLAVWRNTVTNYFIVNLSLADVLVTAICLPASLLVDITEGQALCKVIPYLQAVSVSVAVLTLSFIALDRWYAICHTARRARGSILGIWAVSLAVMVPQAAVMLYPKIYHSCFFIVTYLAPLGLMAMAYFQIFRKLWGR-QIPFLAEVKQMRARRKTAKMLMVVLLVFALCYLPISVLNVLKRVFDREAVYACFTFSHWLVYANSAANPIIYNFLSGKFREQFKAAF

>M._mus_OX2

YEWVLIAGYIIVFVVALIGNVLVCVAVWKNTVTNYFIVNLSLADVLVTITCLPATLVVDITEGQSLCKVIPYLQTVSVSVSVLTLSCIALDRWYAICHTAKRARNSIVVIWIVSCIIMIPQAIVMVYPKMYHICFFLVTYMAPLCLMILAYLQIFRKLWCR-QIPVAAEIKQIRARRKTARMLMVVLLVFAICYLPISILNVLKRVFDRETVYAWFTFSHWLVYANSAANPIIYNFLSGKFREEFKAAF

>G._gal_OX2

YEWALIAGYIVVFIVALVGNVLVCIAVWKNTVTNYFIVNLSLADILVTITCLPATLVVDITEGHHLCKAIPYLQTVSVSVSVLTLSCIALDRWYAICHTAKRARNSIIIIWIVSCIIMIPQAIVMVYPKMYHTCFFLVTYMAPLCLMVLAYLQIFRKLWCR-QIPVAAEIKQIRARRKTARMLMVVLLVFALCYLPISILNILKRVFDRETVYAWFTFSHWLVYANSAANPIIYNFLSGKFREEFKAAF

>A._car_OX2

YEWVLIAGYIIVFLVALIGNILVCVAVWKNTVTNYFIVNLSLADVLVTITCLPATLVVDITEGDSLCKGIPYLQTVSVSVSVLTLSCIALDRWYAICHTAKRARNSIIIIWIVSCIIMIPQAIVMIYPKLYHTCFFLITYMAPLCLMVLAYLQIFQKLWCR-QIPVTAEIKQIRTRRKTARMLMVVLLVFALCYLPISILNILKRVFDRETVYAWFTFSHWLVYANSAANPIIYNFLSGKFREEFKAAF

>X._tro_OX2

YEWVLIVGYIIVFIIALIGNILVCVAVWKNTVTNYFIVNLSLADVLVTIICLPATLLVDITEGKTLCKVIPYLQTVSVSVSVLTLSCIALDRWYAICHTAKRAQQSIVIIWIVSCAIMIPQAIVMIYSKVYHICFFCITYMVPLCLMILAYLQIFRKLWCR-QIPVAAEIKQIHARRKTARMLMVVLLVFALCYLPISILNILKRVFDRETVYAWFTFSHWLVYANSAANPIIYNFLSGKFREEFKAAF

>D._rer_OX2

YEWVLIAGYILVFLVSLVGNTLVCFAVWKNTVTNYFIVNLSFADILVTITCLPASLVVDITEGQTLCKILPYLQTISVSVSVLTLSCIAQDRWYAICHTAKRARKSIVLIWLVSCIMMIPQAVVMIYPKVYHTCFFIVTYFAPLCLMVLAYIQICHKLWCQ-QIPVSAEAKQVKARRKTARMLMVVLFVFALCYLPISILNIMKRVFNRETVYAWFTFSHWLIYANSAANPIIYNFLSGKFREEFKAAF

>L._ocu_OX1

YEWVLIAGYIFVFVVALTGNILVCLAVWRNTVTNYFIVNLSLADLLVTAICLPVSLVVDITEGQTLCKVIPYLQTVSVSVSVLTLSFIALDRWYAICHTARRARNSIVLIWLLSLAIMVPQAVVMVYPRVYHVCFFLVTYLAPLCLMFMAYFQIFRKLWSR-QIPPSAEVKQLRARRKTAKMLLVVLLVFSLCYLPISVLNVLKRVSDREAIYAWFTFSHWLVYANSAANPIIYNFLSGKFRGEFKAAF

>L._ocu_OX2

YEWVLIAGYIIVFFISLIGNTLVCIAVWKNTVTNYFIVNLSFADVLVTITCLPASLVVDITEGQTLCKVLPYVQTTSVSVSVLTLSCIALDRWYAICHTAKRARKSIVIIWIVSCVIMIPQAIVMIYPKVYHICFFIVTYLAPLCLMVLAYIQIFHKLWCQ-QIPAAAEIKQIRARRKTARMLMVVLFVFALCYLPISVLNVMKRVFDREAVYAWFTFSHWLIYANSAANPIIYNFLSGKFREEFKAAF

>H._sap_NRFFR

VAAMFIVAYALIFLLCMVGNTLVCFIVLKNTVTNMFILNLAVSDLLVGIFCMPTTLVDNLITDNATCKMSGLVQGMSVSASVFTLVAIAVERFRCIVHTLRKALVTIAVIWALALLIMCPSAVTLGMRRVYTTVLFSHIYLAPLALIVVMYARIARKLCQA-PGEEAADPRASRRRARVVHMLVMVALFFTLSWLPLWALLLLID--LHLVTVYAFPFAHWLAFFNSSANPIIYGYFNENFRRGFQAAF

>H._sap_QRFPR

AKLALVLTGVLIFALALFGNALVFYVVTRSTVTNIFICSLALSDLLITFFCIPVTMLQNISDGAFICKMVPFVQSTAVVTEILTMTCIAVERHQGLVHTNRRAFTMLGVVWLVAVIVGSPMWHVQVHQKIYTTFILVILFLLPLMVMLILYSKIGYELWIKRGIHGKEMSKIARKKKRAVIMMVTVVALFAVCWAPFHVVHMMIE--DDVTIKMIFAIVQIIGFSNSICNPIVYAFMNENFKKNVSSAV

>H._sap_GALR2

EAVIVPLLFALIFLVGTVGNTLVLAVLLRGSTTNLFILNLGVADLCFILCCVPFQATIYTLDGSLLCKAVHFLIFLTMHASSFTLAAVSLDRYLAIRYTPRNALAAIGLIWGLSLLFSGPYLSYYPRRRAMDICTFVFSYLLPVLVLGLTYARTLRYLWRA--DPVAAGSGARRAKRKVTRMILIVAALFCLCWMPHHALILCVW--LTRATYALRILSHLVSYANSCVNPIVYALVSKHFRKGFRTIC

>H._sap_ENDRB

FKYINTVVSCLVFVLGIIGNSTLLRIIYKNNGPNILIASLALGDLLHIVIDIPINVYKLLAEGAEMCKLVPFIQKASVGITVLSLCALSIDRYRAVASVPKWTAVEIVLIWVVSVVLAVPEAIGFFYKTAKDWWLFSFYFCLPLAITAFFYTLMTCEMLRK--GMQIALNDHLKQRREVAKTVFCLVLVFALCWLPLHLSRILKLCELLSFLLVLDYIGINMASLNSCINPIALYLVSKRFKNCFKSCL

>B._jap_OX

YEWALIIAYILVFLLALIGNGLVCFVVIRNTVTNYFIANLSAGDLLVTIICLPPTLVVDIMEGETMCKIIPYLQMVSVSVSVLTLCAIAVERWYAIVHTNARARTIICLIWVVSLSIMAPLIPMYIYGKIYHAAIVMVLFGVPIVLMMVSYCMIVWKLWSD-QVPDKTAENTVQSRRKVARMLVAVVVVFAICYIPLMILTFLKRVYDRSGLYAAFTVSHWLLYLNSAINPLIYNFMSEKFRSEFKASL

>B._bel_OX

YEWALIVGYILVFLLALIGNGLVCFVVARNTVTNYFIANLSAGDLLVTIICLPPTLVVDIMEGETMCKIIPYLQMVSVSVSVLTLCAIAVERWYAIVHTNARARTIICLIWVVSLSIMAPLVPMYIYGKIYHAAIVMVLFGIPIVLMMVSYCMIVWKLWSD-QVPDKTAENTVQSRRKVARMLVAVVVVFAICYIPLMILTFLKRVYNRQGVYAAFMVSHWLLYLNSAINPLIYNFMSEKFRSEFKASL

>S._pur_OX

HEYFLIAIYFIIFFVAIVGNSMVCIAILKNTVTNYYIMNLATTDIMIAVVCLPITITVDVSEGQTACYLIPYFQLVLVCASIYTLMMIAVDRYLAICHRASRTLLTIALVWVVSFFIALPVAVVNVWEKLYHTAFFLAVYIVPLAVIGVAYTRVCRRLWSG--IPSKSTEAQLKSRRKVASMLIVVVVTFAICFFPFQLLNVLKKHNQYNAVYIPYIIGHLMAFINSAINPIIYNFMSAKFRQAFKSMF

>S._kow_OX

HEWFLISVYALVFLMALVGNVLVCFAVLRNTVTNYYIVNLSFADILVSLICLPVTVTFETTEGDLACKIIPYVQVVSMSVSVLTLSAIAVDRYFAICQTAKRTLTIIFSIWLVSFIIPIPQAIVYIQQKIYHVALVLVIYVIPLLLIGIAYLFICRQLWAT--IPNRTTMSQLKSRRKVANMLIIVAILFALCYLPLHLLNIIRQFPDRHSFHIPFLVAHWLAFANSATNPVVYNFLSAKFRKEFKAAF

>M._sex_ATR

YEWVLIATHSIVFLTGLIGNALVCIAVYRNTVTNYFIVNLAVADFMVILFCLPATVLWDVTEGDVLCKMLLYFQSVSVTVSVLTLTFISVDRWYAICFTTSRAKTAILIIWILSLSFNSPDLVVLA-DPVWHILKVVFIYTIPLLLMTVAYLQIVRVLWHSDKIPAPAEQTQLRSRRKAAKMLVAVVVMFAVCYFPVHLLSVLRYTLQSDAITFLALVSHVMCYANSAVNPLIYNFMSGKFRREFRRAF

>S._gre_ATR

YEWILIAMHSLVFVAGLVGNALVCLAVYRNTVTNYFIVNLAVADFMVILFCLPPTVLWDVTEGTGLCKVVLYLQTVSVAVSVLTLTFISVDRWYAICFTTGRAKTAIAIIWLLALAFDIPELVVLS-EMVFHGAKSLLLYTLPLLFMSVAYFQIVRVLWRSDNIPNSTTEAQLRSRRKAAKMLVAVVAMFAICYLPVHLLNILRYTVQNDTTSAISMLSHWLCYANSAVNPVIYNFMSGKFRAEFRRLF

>A._aeg_ATR

AEWILIASHSVVFIMGLVGNALVCIAVYTNTVTNIFIVNLAVADFFVILFCLPPTVVWDVTEGKAMCKVVIYFQTVSVTVSVLTLTYISIDRWYAICFRPERAWRFIAVIWLIGFLSDLPEFLVLK-EKTFYIVKFVFLYSLPLLFMTIAYFQIVRVLWRSDTIPNTSTMGQLRARRKAAKMLVAVVVMFASCYFPVHMLNVARYTFQSDVVAVLSLFSHWLCYANSAVNPVIYNFMSGKFRREFKNAL

>F._occ_ATR

MEWVIIAMHCAVFIGGLVGNALVCLAVYRNTVTNYFIVNLAVADFLVILMCLPPTVLWDVTEGTALCKIVLYFQTVSVTVSVLTLTTISVDRWYAICFTTSRAKKAIIIIWLLALSFDVPELVVLS-ETTYHCVKTLFLFFLPLAFMTVTYVQIVKVLWSKTNIPSISASSQILSRRKAAKMLVVVVLMFFICYLPVHLLSILRYTMQTELMTVTAMFVHWLCYANSAVNPLIYNFMSGKFRGEFRLAF

>H._arm_ATR

YEWVLIGTHTLVFITGLVGNALVCVAVYRNTVTNYFIVNLAAADFMVILFCLPATVVWDVTEGDVLCKMLLYFQSVSVTVSVLTLTFISVDRWYAICFTTGRAKTAILIIWTLSLIFNAPELVVLS-DLVWHIIKVIFIYTLPLLLMTVAYYQIVKVLWRSEKIPAPAEQTQLRSRRKAAKMLVAVVIMFAVCYFPVHLLSVLRYTLQNDVITCLALISHVMIYANSAINPLIYNFMSGKFRREFRRAF

>D._ple_ATR

YEWVLIGVHTTVFVIGLIGNLLVCLAVYRNTVTNYFLVNLAVADFMVLLFCLPATVLWDVTEGDALCKILLYIQSVSVTVSVLTLTFISVDRWYAICFTINSAKTAILVIWALSLVFNTPELVVLS-DLIWHIIRIVFVYTVPLLLMTVAYHQIVRVLWSSQKIPASAEQIQLQSRRKAAKMLVAVVVMFAVCYFPVHLLSVLRY-LQNDMITCLALVSHVLCYVNSAINPLIYNFMSGKYRREFRRVF

>B._ter_ATR

WDWVLIASHSVVFVVGLVGNALVCIAVYRNTVTNYFIVNLAVADFLVLLLCLPFTVLWDITEGLTLCKAVPYLQTVSVTVSILTLTFISIDRWYAICFTTGRAKSAIIGIWAAALLFDIPDLVVLS-QVAFTIVKLIFLYTGPLIFMSVAYWQIVKVLWRS-NIPGGNPEVQLRSRRKAAKMLVTVVITFAICYFPVHLLSVLRYTTSNKWINAISLIAHGLCYFNSAVNPLIYNFMSGKFRKAFRRTF

>P._dum_ATR

FEWVLIVLYIQVFTIGLCGNLLVCFAVWRNTVTNYFIVNLAVADLLVIIICLPPTVLVDVSEGAVMCKVVHYMQGVSVSVSVLTLSCISVERWYAICHTTTRVRSIIVVTWVVALVILIPELIVLYNPMAYELFKMVALYFLPIILMSVTYGNIVICLWSN-AIPRTTAEAQLIARRKAAKMLIAVVVMFGVCYLPVHLTNILRYAKESENITFFPLVAHWLCYFNSAINPVIYNFMSARFRNEFKHAC

>C._int_OX_(X2)

AEWFVMSLYVLVFLISIIGNCLTIAFILRRTTINYFMLNLALADIMVTIICLPPTLMVDFMEGQFLCKFTPYLQMAVTSVSSLSLGAIAVNRWFVVCHSAKHALLTMTSIWLFSLITLCPIIFVTLHQAVFHIYYVTVCYALPLMVMAIAYTNVFRKLSYT-KIPCKIKRNLIQSRKRSGRIQVALVVVYFLCYSPAMVLDLIRRTSHRESTYFLFAIAHLLVYLNSALNPIIYNCFSVRVSGEILFVQ

>C._int_OX_(X1)

AEWFVMSLYVLVFLISIIGNCLTIAFILRRTTINYFMLNLALADIMVTIICLPPTLMVDFMEGQFLCKFTPYLQMAVTSVSSLSLGAIAVNRWFVVCHSAKHALLTMTSIWLFSLITLCPIIFVTLHQAVFHIYYVTVCYALPLMVMAIAYTNVFRKLSYT-KIPCKIKRNLIQSRKRSGRIQVALVVVYFLCYSPAMVLDLIRRTSHRESTYFLFAIAHLLVYLNSALNPIIYNCFSVQFRKEFRLTF

>C._sav_OX

TEWFVISLYAIVFLTSIVGNCLTIAFILRRTTINYFMLNLALADIMVTIICLPPTLMVDFMEGQFLCKFTPYLQMAVTSVSSLSLGAIALNRWFVVCHSAKNSLISMACIWLFSLITLCPVVFVTLHGAVFHVYFVTVCFALPLVVMAIAYTSVFRKLSRT-KVT---KPNLISSRKRSGRIQVALVIVYFLCYSPAMVLDVIRRTSHRESTYFMFAIAHLLMYLNSALNPIIYNCFSVQFRKEFRLTF

>C._gig_ATR

WEWGIIILYALTFIVGLSGNVLVCFAVWRNTVTNIFIVNLAIADLAVIIICLPPTLLSDVTEGFAMCKIALFLQTTSVAVSVFTLSAISVERWYAICYTKRRAKIIILVIWIIAFLLALPEVIVATNQVVYQSVIIVLMYLLPLVLMTVTYSMIAVVLWTG-KIPVNRAEEQLESRKKAAKMLITVVIGFAVCYFPVHLFNILRYADAPRMIQVLSMISHWLPYLNSSINPIIYNFMSAKFRKEFTAAC

>O._bim_ATR

GEWILVVIFIILFIVGLVGNFLVCYAVIKNTVTNLFIMNLAIADFMVILICLPSSLLVDVSEGEVMCKIFLYLQTVSVAVSVLTLSAISIERWYAICHTASRARNIILTIWLLSACVASPDLVTAS-QFIYQMFLFIALYFLPFCLMAFTYTRITLVLWRE-DIPNPNTNAQLQTRRKAAKMLITVVIVFGICNLPVHILNIVRYANNLKAISIFSLISRLLCYVNSAINPIIYNFMSAKFRKEFKSVC

>O._myk_OX2

YEWVLIVGYIIVFFVSLIGNTLVCFAVWKNTVTNCFIVNLSFADVLVTITCLPASLVVDITEGNTLCKILPYLQTISVSVSVLTLSCIALDRWYAICHTARRARKSILLIWGVSCIIMIPQAIVMVYPKVYHTCFFIVTYFAPLCLMVLAYIQICHKLWCQ-QIPVSAEIKQVRARRKTARMLMVVLFVFALCYLPISVLNIMKRVFSRETVYAWFTFSHWLIYANSAANPIIYNFLSGKFRAEFKAAF

>C._mil_OX2

YEWVLIAAYIAVFVVALVGNVLVCVAVWKNTVTNYFIVNLSFADVLVTIICLPASLVVDITEGQIFCKVIPYLQTVSVSVSVLTLSCIALDRWYAICHTAKRARNSIIIIWIVSCVLMTPQAIVMVYPKVYHICFFIVTYMGPLCLMILAYFQIFRKLWCR-QIPVAAEIKQIRARRKTARMLMVVLFFFAVCYLPISILNVLKRVFDRATVYAWFMFSHWLVYANSAANPIIYNFLSGKFREEFKAAF

>L._cha_OX2

YEWALIAGYIIVFIVALFGNILVCVAVWKNTVTNYFIVNLSLADVLVTITCLPASLVVDITEGQTLCKVIPYLQTVSVSVSVLTLSCIALDRWYAICHTAKRARNSIIIIWIVSCVIMIPQAIVMIYPKVYHICFFLVTYMAPLCLMILAYLQIFHKLWCR-QIPVAAEIKQIHARRKTARMLMVVLFIFALCYLPISILNILKRVFDRETVYAWFTFSHWLVYANSAANPIIYNFLSGKFREEFKAAF
